# Revision of the Plant Bug Genus *Tytthus* (Hemiptera, Heteroptera, Miridae, Phylinae)

**DOI:** 10.3897/zookeys.220.2178

**Published:** 2012-09-10

**Authors:** Thomas J. Henry

**Affiliations:** 1 Systematic Entomology Laboratory, Agricultural Research Service, United States Department of Agriculture, c/o National Museum of Natural History, MRC-168, Smithsonian Institution, Washington, D. C. 20013-7013

**Keywords:** Insecta, Hemiptera, Heteroptera, Miridae, revision, new species, hosts, distribution, predatory habits, phylogeny

## Abstract

The phyline plant bug genus *Tytthus* Fieber, previously containing 19 species, is revised. *Isoproba* Osborn and Drake, 1915, incorrectly placed in the subfamily Bryocorinae, tribe Dicyphini, is synonymized as a junior synonym of *Tytthus* Fieber, **syn. n.**; the only included species, *Isoproba picea* Osborn and Drake is transferred to *Tytthus*, **comb. n.,** as the senior synonym of *Tytthus hondurensis* Carvalho, **syn. n.**; and *Tytthus koreanus* Josifov and Kerzhner, 1972 is synonymized with *Tytthus chinensis* (Stål 1860), **syn. n.**; and a lectotype for *Tytthus parviceps* is designated. The six new species *Tytthus femoralis* from Cuba, Ecuador, Guatemala, Jamaica, Mexico, and Peru,*Tytthus fuscicornis* from New Mexico (USA), *Tytthus mexicanus* from Mexico, *Tytthus pallidus* from Brazil and Panama, *Tytthus uniformis* from Arizona and New Mexico (USA), and *Tytthus wheeleri* from the eastern United States are described, bringing the total number of species for the genus to 24. A color adult habitus illustration of *Tytthus wheeleri*, color photographs for each species (except *Tytthus juturnaiba* Carvalho and Wallerstein), illustrations of male genitalia, scanning electron photomicrographs of selected structures of certain species, and an identification key are provided to facilitate species recognition. A phylogenetic analysis is offered to help infer relationships.

## Introduction

Members of the phyline plant bug genus *Tytthus* Fieber (1864) specialize in feeding on delphacid planthopper eggs, making them of great importance on agricultural monocots, such as rice and sugarcane, where Delphacidae are especially destructive (Wheeler 2001). The introduction of the South-Pacific *Tytthus mundulus* (Breddin) into Hawaii (Muir 1920) that prevented the destruction of the Hawiian sugarcane crop by the sugarcane delphacid, *Perkinsiella saccaricida* Kirkaldy, represents one of the classic examples of successful biological control (Zimmerman 1948).

Prior to this study, 19 species of *Tytthus* were recognized worldwide. Of these, 16 occur in the New World, including two Holarctic and one circumtropical species (Afrotropical, Neotropical, and southern Oriental). Three additional species are known from eastern and southeastern Asia, Australia, and the Indo-Pacific. *Tytthus* was long confused with and considered a junior synonym of the remarkably similar-appearing orthotyline genus *Cyrtorhinus* Fieber, 1858 (Reuter 1875c), until Carvalho and Southwood (1955) showed that the genus belonged in the subfamily Phylinae based on pretarsal structures and male genitalia.

Schuh (1974) included *Tytthus* in his new tribe Leucophoropterini, a group of predominately African, Australian, and South-Pacific taxa, based on the hairlike parempodia, the U-shaped endosoma, the relative small size of the male genitalia, the shape of the right paramere, and the simple female posterior wall. He also noted that *Tytthus* was not ant mimetic as are most other leucophoropterines. Kerzhner and Josifov (1999), however, conservatively placed Leucophoropterini in synonymy under Phylini after indicating that they were following Linnavuouri (1993), who thought it premature to split the tribe. Schuh (1995), however, maintains Leucophoropterini in the online version of his world mirid catalog (http://research.amnh.org/pbi/catalog/), until more information on relationships becomes available. More recently, Menard (2011) and Menard and Schuh (2011) gave convincing molecular and morphological evidence supporting the monophyly of Leucophoropterini, but also showed that *Tytthus* consistently grouped outside the tribe. As a consequence, I follow Menard (2011) and Menard and Schuh (2011) in transferring *Tytthus* from Leucophoropterini to nominate tribe Phylini.

In this paper, I revise the genus *Tytthus*; give a diagnosis and new host and distribution records for previously described species; synonymize the genus *Isoproba* Osborn and Drake as a junior synonym of *Tytthus* and transfer its only included species to *Tytthus* as the senior synonym of *Tytthus hondurensis* Carvalho; and synonymize *Tytthus koreanus* Josifov and Kerzhner under *Tytthus parviceps* (Reuter). Six new New World species are described; 18 previously known species are redescribed; a revised identification key is provided to help distinguish the species of the genus; and a phylogenetic analysis is presented.

## Methods

The male genital capsule was dissected and placed in room-temperature, 10% KOH solution for one to two days or until softened and cleared, after which it was rinsed in water and placed in a depression slide containing glycerol. The endosoma, right and left parameres, and phallotheca were dissected and pencil sketched using a Nikon E400 compound microscrope and drawing tube. Final illustrations were digitally rendered using Adobe Photoshop CS4.

Photomicrographs were taken using either an AMRAY 1810 or a Zeiss EVO/MA15 scanning electron microscope. Specimens were glued to standard SEM stubs, sputter coated, and examined at 6–10 KV.

Color images were captured using an EntoVision Imaging Suite that included a JVC KY-75 3CCD digital camera mounted to a Leica M16 zoom lens via a Leica z-step microscope stand. Multiple focal planes were merged using Cartograph 5.6.0 (Microvision Instruments, France) software. Plates of color habitus images (not to scale), SEM photomicrographs, and male genitalia were created using Adobe Photoshop CS4 and striped and numbered in Adobe Illustrator CS4.

Matrix code labels were attached to more than 1,000 specimens examined. These codes, referred to as unique specimen identifers (USIs), are a way to uniquely identify specimens and are stored in a database developed for the NSF Planetary Biodiversity Project awarded to R. T. Schuh (American Museum of Natural History, New York, NY) and G. Cassis (University of New South Wales, Sydney, Australia). The full code contains the prefix “AMNH_PBI, an eight-digit number, and the specimen depository, for example (AMNH_PBI 00162206) (USNM).” USI codes are included in the specimen data listed at the end of each species treatment. To save space, the “AMNH_PBI” prefix has been omitted. Data for several hundred additional specimens also were recorded, but matrix code labels were not available for these collections (e.g., BMNH, NMW) at the time the data were captured and, thus, were not entered into the database and therefore lack USI numbers.

Specimen measurements were taken as follows: Length (dorsal length from apex of clypeus to posterior margin of hemelytral membrane); length to base of cuneus (dorsal length from apex of clypeus to base of cuneus); width across hemelytra (widest dorsal width across hemelytra, usually just above each cuneus); head length (lateral length from posterior margin of pronotum to apex of clypeus); head width (dorsal width across eyes); interocular width (greatest dorsal width between eyes); labium (length from a base of labrum to apex of segment IV); antennal segment length (self explanatory); pronotum length (dorsal median length); pronotum basal width (dorsal width across posterior margin). I follow Cassis (2008) in using the term “endosoma” rather than “vesica” for the male intromittent organ. Other terminolgy follows conventional use in the contemporary literature, as defined in Nichols (1989).

The phylogenetic analysis was performed using Winclada (Nixon 1999–2002), implementing the island hopping or the rachet function to run NONA (Goboloff 1999) and TNT (Goloboff et al. 2008) using the traditional and random addition sequence functions. All cladograms were generated using Winclada.

Plant names follow the International Plant Names Index (2011) and the National PLANTS Database (USDA, NRCS, 2011).

Abbreviations and curators for collections cited in the paper are as follows:

**AMNH** (American Museum of Natural History, New York; R. T. Schuh);

**BMNH** (The Natural History Museum, London; M. Webb);

**BPBM** (B. P. Bishop Museum, Honolulu, Hawaii; S. Myers);

**CAS** (California Academy of Sciences, San Francisco; N. Penny);

**CDFA** (California Department of Food & Agriculture, Sacramento; R. Garrison);

**CNC** (Canadian National Collection of Insects, Ottawa, Ontario; M. D. Schwartz and R. G. Foottit);

**DEBU** (University of Guelph Insect Collection, Guelph, Ontario; Steven Marshall);

**NMW** (National Museums and Galleries of Wales, Cardiff; M. Wilson);

**OSU** (Ohio State University, Columbus; C. A. Triplehorn and N. E. Johnson);

**SDNH** (San Diego Natural History Museum, San Diego; M. Wall);

**SNU** (Insect Biosystematics Laboratory, College of Agriculture and Life Science, Seoul National University, Seoul, South Korea; S. H. Lee and R. K. Duwal);

**UCB** (University of California, Berkelely; C. Barr);

**UCD** (University of California, Davis; L. Kimsey);

**UK** (University of Kansas, Lawrence, Kansas; Z. H. Falin);

**UTSU** (Utah State University, Logan; W. J. Hansen)

**USNM** (National Museum of Natural History, Washington, D. C.; T. J. Henry);

**VMNH** (Virginia Museum of Natural History, Martinsville; Richard L. Hoffman).

**WSU** (Washington State University, Pullman; Richard S. Zack).

Species are arranged alphabetically in the text.

## Taxonomy

### Tytthus

Fieber

http://species-id.net/wiki/Tytthus

Tytthus Fieber 1864: 82. Type species: *Capsus geminus* Flor, 1860. Designated by Kirkaldy 1906: 128.
Cylloceps Uhler, 1893: 711. Type species: *Cylloceps pellicia* Uhler, 1893. Monotypic. Synonymized by Carvalho and Southwood 1955: 17.
Periscopus Breddin 1896: 106. Type species: *Periscopus mundulus* Breddin, 1896. Monotypic. Preoccupied by *Periscopus* Fitzinger, 1843 (Reptilia); synonymized by Carvalho and Southwood 1955: 17.
Breddiniessa Kirkaldy 1903: 13. New name for *Periscopus* Breddin, 1896; synonymized by Carvalho and Southwood 1955: 17.
Isoproba Osborn and Drake 1915: 533. Type species: *Isoproba picea* Osborn and Drake, 1915. Monotypic. syn. n.

#### Diagnosis.

Species of *Tytthus* are characterized by the small size (lengths ranging from 1.08 mm in brachypterous males of *Tytthus wheeleri* to more than 3.60 mm in *Tytthus mundulus*), the relatively broad to nearly round head, usually with a pale yellow spot on the vertex bordering the inner margin of each eye; slightly protruding eyes not touching the anterior margin of the pronotum; smooth, shiny, trapeziform to campanulate pronotum, with lateral margins straight to weakly concave and moderately to strongly flared humeral angles; flat to weakly raised calli; subparallel hemelytra, often brachypterous or abbreviated, with the membrane and cuneus greatly reduced; slender claws with setiform parempodia; slender, tapered abdomen; small genital capsule; simple, C- to weakly S-shaped endosoma, lacking a secondary gonopore; mitt-shaped left paramere; and simple, round to elongate-oval right paramere.

#### Description.

Elongate subparallel to elongate oval species. Head shiny, impunctate, broader than long, sometimes becoming broadly rounded, especially in males, always slightly wider than anterior margin of pronotum; eyes prominent, more so in males, finely granulate, usually with scattered, fine, short setae; in dorsal view, frons and clypeus weakly rounded to prominent and pointed anteriorly; interocular space proportionately narrower in males (because of more prominent eyes) than females, nearly always with a small to large yellow or pale spot adjacent to inner margin of each eye; posterior margin nearly straight, with eyes nearly touching anterior margin of pronotum, to sometimes more narrowed behind eyes forming a necklike area more distinctly separating eyes from pronotum. Labium extending from bases of hind coxae to well onto abdomen near segment III or IV; segment I extending from base of head to bases of forecoxae. Antennal segment I shortest, stoutest; segment II longest; segment III longer than to subequal to segment IV. Pronotum shiny, impunctate, calli usually prominent, often with a glaucous sheen; subrectangular to trapeziform, especially in flightless brachypters, to strongly campanulate or bell-shaped in macropters. Mesoscutum broadly exposed in macropters; concealed by posterior margin of pronotum in brachypters. Scutellum well developed, equilateral. Hemelytra translucent, opaque white to bicolored with dark clouds, transverse bands, or extensive dark areas; macropterous or brachypterous, if only one sex brachypterous, always the female; fully macropterous hemelytra with each cuneus entire and membrane fully developed, extending well beyond apex of abdomen; brachyterous hemelytra (see discussion on brachyptery) abbreviated, ranging from a partially shortened membrane, extending only to apex of abdomen, to a strongly abbreviated membrane represented by only a remnant fringe on posterior edge of coleopteriform corium and clavus, with cuneus absent; in most extreme forms, only short hemelytral pads present, entirely lacking the cuneus and membrane, and extending only to abdominal terga III or IV. Lengths range in macropterous males from 2.14–3.42 mm; brachypterous males 1.08–1.28 mm; macropterous females 1.80–3.52 mm; and brachypterous females 1.44–1.68 mm. Ventral surface shiny, impunctate. Ostiolar evaporative area with a prominent auricle, curving posteriorly, gland opening large and distinct. Legs slender; femora unspotted, sometimes infuscated; tibiae slender, with or without distinct spines; tarsi slender, lengths of segment II and III subequal; claws elongate, slender, parempodia setiform.

Male genitalia: Endosoma relatively simple C-shaped to S-shaped, composed of a single, simple tube, often distally truncate or concave, lacking an apparent secondary gonopore. Left paramere mitt-shaped, with two arms and a narrow basal stem; right arm longest, widest, and most prominent, distally acute to rounded, gradually narrowing from base to apex, often broadened just before apex; left arm much shorter, distally acute. Right paramere elongate oval to nearly round, with a short basal stem. Phallotheca simple, sheathlike, exposed apex gradually narrowing from base to an acute apex.

#### Discussion.

Members of this genus are so superficially similar to species of the orthotyline genus *Cyrtorhinus* that Reuter (1875c) placed *Tytthus*, in synonymy under it, where it remained for the next 80 years. Even H. H. Knight (1923, 1925, 1931), North America’s most knowledgeable and prolific mirid specialist, failed to recognize the misplacement, and R. L. Usinger (1939), who treated several South Pacific species of *Tytthus* noted “An apparent structural anomaly in *Cyrtorhinus* which has not been given sufficient attention is the absence, in certain species, of arolia between the claws. The presence or absence and form of the arolia is usually a very reliable guide to relationships in Miridae.” Despite the character differences between these taxa, the species remained together under *Cyrtorhinus* until Carvalho and Southwood (1955) documented the obvious differences in male genitalia and pretarsal structure.

Another problematic genus, *Isoproba* Osborn and Drake (1915), has not been mentioned in the primary literature since its original description. Described to accommodate the only included species, *Isoproba picea* Osborn and Drake from Guatemala, it was said to be “readily separated from the [orthotyline] genus *Paraproba* Distant and allied genera by the more globose head and the peculiar shape of the thorax (Osborn and Drake 1915).” Carvalho (1952, 1958), however, without explanation, transferred it to the tribe Dicyphini (then placed it in the subfamily Phylinae), whose members also have generally rounded heads, as well as setiform parempodia. Cassis (1984) noted that he was unable to locate the holotype and, therefore, left it in Dicyphini with “considerable reservation.”

I have studied the holotype of *Isoproba picea* deposited in the Ohio State University collection and, like most species included in the genus *Tytthus*, it has an overall shiny, fuscous to black head, pronotum, and scutellum, pale translucent hemelytra, and slender legs and antennae. The male genitalia are of the same type as for other species of *Tytthus*. The left paramere is mitt-shaped, the right paramere is relatively small, elongate oval, and simple, and the endosoma is slender and C-shaped. *Isoproba picea* differs from other species of *Tytthus* onlyin having a more distinctly rounded or bulbous head that is narrowed posteriorly into a short neck, especially in males, and the shallowly convex eyes hardly protruding from the side of the head. In addition, I have discovered that *Tytthus hondurensis* Carvalho (1984) is a junior synonym of *Tytthus piceus*. As a consequence, *Isoproba* is placed as a junior synonym of *Tytthus*.

*Wing polymorphism*: Slater (1975) separated the various types of wing modifications in the family Lygaeidae (sensu lato) into seven main categories: 1) Aptery (wings entirely absent); 2) Microptery (wings reduced to widely separated pads; 3), Staphylinoidy (wings have the clavus and corium indistinguishably fused into a coriaceous pad, and the wings meet evenly along the midline for their entire length, and usually cover only the first three abdominal segments); 4, Coleoptery (wings may or may not be reduced, but the coriaceous portion is not reduced but lengthened, the clavus and corium are fused, and the wings meet along the midline but do not overlap); 5) Brachyptery (clavus and corium either distinctly separate or fused, but shorter than in macropters, with only the inner portion of the membrane overlapping; 6) Submacroptery (clavus and corium always separate, with membrane slightly shortened, leaving the last abdominal segment exposed); and 7) Macroptery (wings unmodified, fully developed). Of the species of *Tytthus* exhibiting wing polymorphism, two can be categorized as staphylinoid (*Tytthus alboornatus*, *Tytthus wheeleri*), two as brachypterous (*Tytthus montanus*, *Tytthus piceus*), and four (*Tytthus balli*, *Tytthus fuscicornis*, *Tytthus pubescens*, and *Tytthus uniformis*) as submacropterous. The remaining sixteen species are known only from macropterous individuals.

*Importance in biological control*: It has been documented that most, if not all, species of *Tytthus* are specialized delphacid and, to a lesser extent, leafhopper egg predators. The best documented species, *Tytthus mundulus*, provides a good example of successful classical biological control (Hagen and Franz 1973, van den Bosch and Messenger 1973, Rosen 1985, Wheeler 2001). Frederick Muir (1920) discovered while searching for predators of the sugarcane delphacid in Queensland, Australia, that nymphs and adults fed on delphacid eggs. As a consequence, he brought *Tytthus mundulus* to Hawaii for release into the sugarcane fields. As Usinger (1939) noted, “Muir’s discovery that *Tytthus* (as *Cyrtorhinus) mundulus* (Breddin) lives exclusively on the eggs of the sugar-cane leafhopper, *Perkinsiella saccaricida* Kirkaldy, led to one of the most outstanding successes in the field of biological control of injurious insects.” Zimmerman (1948) summed up the importance of this bug by saying “This one bug has saved the Hawaiian sugar industry and the Territory millions of dollars—its true worth can hardly be estimated.”

Other species also have shown considerable potential in biological control. In South Africa, both *Tytthus mundulus* and *Tytthus parviceps* (Reuter) have been investigated for control of a tropiduchid, *Numicia viridis* Muir, on sugarcane (Carnegie and Harris 1969). Although *Tytthus mundulus* was the better-known predator, *Tytthus parviceps* was more easily reared and showed the greatest potential for controlling *Numicia viridis*. Jhansi et al. (2002) studied the biology and prey preferences of *Tytthus parviceps* on planthoppers and leafhoppers on rice in India, including the brown planthopper, *Nilaparvata lugens* (Stål). The Holarctic species *Tytthus pubescens* (Knight) and *Tytthus pygmaeus* (Zetterstedt) are known to prey on leafhoppers and delphacids in England (Southwood and Leston 1959, Rothschild 1963, Wheeler and Henry 1992). In coastal eastern North America, Döbel and Denno (1994) considered *Tytthus alboornatus* (Knight) and *Tytthus vagus* (Knight) among the major predators of saltmarsh delphacids on two species of *Spartina* (Poaceae). For additional information on the hosts and habits of these predatory bugs, see the respective species within this revision.

**Figure 1.**
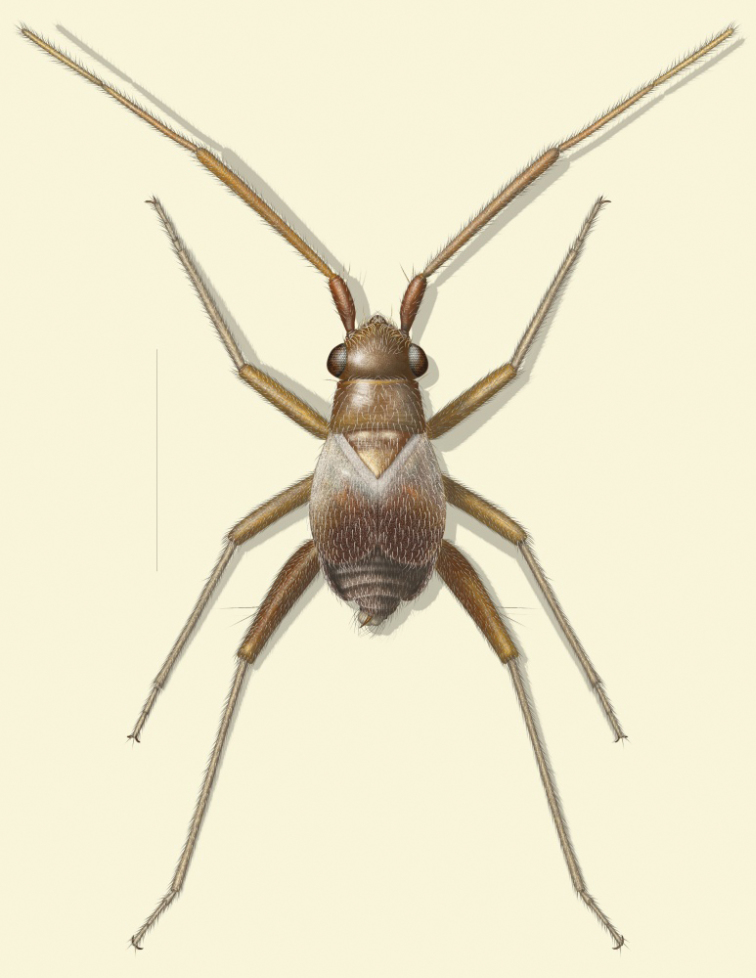
*Tytthus wheeleri*, sp. n., adult brachypterous ♂.

#### Key to Species of *Tytthus*

**Table:** 

1	Bases (or “knees”) of tibiae narrowly fuscous	2
–	Tibiae uniformly pale or dark, bases never fuscous	6
2	Apex and base of antennal segment I narrowly pale yellow; ventral surface of antennal segment II in male with a row of short, erect, stiff setae (Fig. 59), in addition to thickly set semierect or recumbent setae	3
–	Apical fourth of antennal segment I pale yellow, remainder of segment black; ventral surface of antennal segment II in male with only semierect or recumbent setae	4
3	Anterior half of pronotum mostly or entirely pale yellow; pale spot on vertex large and distinct (Figs 32, 34); distribution: circumtropical	*Tytthus parviceps* (Reuter)
–	Anterior half of pronotum uniformly dark or with only traces of pale yellow; pale spot on vertex small, indistinct (Figs 9, 10); distribution: southeastern Asia, Indo-Pacific, Australian	*Tytthus chinensis* (Stål)
4	Hind femur uniformly pale yellow; antennal segment II dark brown to fuscous, sometimes darker at base (Figs 19, 20); distribution: Mexico	*Tytthus mexicanus* Henry, sp. n.
–	Hind femur fuscous distally; antennal segment II dark or pale	5
5	Hind femur fuscous on distal two thirds, basal third and apex narrowly pale; antennal segment II pale, with a fuscous band at base (Figs 13, 14); distribution: Mexico to Ecuador, and the West Indies	*Tytthus femoralis* Henry, sp. n.
–	Hind femur fuscous on apical third; front and middle femora sometimes infuscated; antennal segment II uniformly fuscous (Fig. 12); distribution: Argentina	*Tytthus entrerianus* Carvalho & Carpintero
6	Antennal segment I mostly pale yellow to brownish yellow, at most narrowly dark at base	7
–	Antennal segment I mostly dark brown or black, at most pale at base and/or apex	14
7	Antennal segment I pale, with a dark ring at base, if presence of ring indistinct, hemelytra extensively dark brown or fuscous, with base of clavus and corium pale or whitish (Fig. 28)	8
–	Antennal segment I uniformly pale, without a dark ring at base, sometimes narrowly fuscous apically; hemelytra uniformly yellow, pale yellow invaded with dark brown areas, or dark smoky brown with costal margins paler (Figs 5, 24, 53)	12
8	Hemelytra largely fuscous to black, pale at base of clavus and corium only	9
–	Hemelytra uniformly pale or whitish, at most with dark smoky brown shading	10
9	Larger species, length greater than 2.65 mm in macropters; cuneus uniformly dark brown (Figs 21–23); distribution: Arizona, Montana, and Utah	*Tytthus montanus* Carvalho & Southwood
–	Smaller species, length less than 2.50 mm in macropters; cuneus pale or whitish, cuneus lacking in brachypters, apex of each hemelytron pale or white (Figs 2–4); distribution: coastal eastern North America	*Tytthus alboornatus* (Knight)
10	Antennal segments II–IV intermixed with long, erect and semierect setae nearly as long as diameter of segment; posterior angles of pronotum often pale; hemelytra uniformly pale or translucent white (Figs 38, 39); distribution: Holarctic	*Tytthus pubescens* (Knight)
–	Antennal segment II-IV with only short, recumbent setae much shorter than diameter of segment; pronotum always uniformly fuscous to black; hemelytra often clouded with smoky brown	11
11	Head bulbous in both sexes; length of antennal segment I subequal to width of interocular space; pronotal calli prominent, with a distinct glaucous sheen (Figs 35–37); distribution: Mexico to Colombia, and Maryland to Florida in the eastern United States	*Tytthus piceus* (Osborn & Drake)
–	Head not bulbous, much broader than long; length of antennal segment I greater than width of interocular space; pronotal calli less prominent and without obvious glaucous sheen (Figs 30, 31); distribution: Brazil and Panama	*Tytthus pallidus* Henry, sp. n.
12	Head and pronotum black; antennal segment II fuscous or black (Figs 24, 25); distribution: Indo-Pacific, Australia, introduced into Hawaii	*Tytthus mundulus* (Breddin)
–	Head and pronotum entirely or extensively pale; antennal segment II pale yellowish brown	13
13	Entirely pale or brownish yellow, including appendages (Figs 53, 53); distribution: central Pacific Region	*Tytthus zwaluwenbergi* (Usinger)
–	Extensively pale or brownish yellow, but with brown mottling and marks on head, pronotum around calli, scutellum, cloud on apical area of corium, and undersurface of thorax and margins of abdomen (Figs 5, 6); distribution: Brazil and Peru	*Tytthus amazonicus* Carvalho
14	Antennal segment I fuscous to black, with apex and/or base pale or yellowish	15
–	Antennal segment I entirely fuscous to black	19
15	Apex and basal one third of antennal segment I pale, leaving a broad fuscous or black band through middle (Figs 26, 27); distribution: widespread through Neotropics, and Florida in the United States	*Tytthus neotropicalis* (Carvalho)
–	Only apex of antennal segment I pale, remainder dark	16
16	Apical one fourth of antennal segment I pale (Figs 41–44); distribution: Holarctic	*Tytthus pygmaeus* (Zetterstedt)
–	Apical one fifth or less of antennal segment I pale	17
17	Antennal segment II pale yellowish brown; hemelytra uniformly pale; hind femur uniformly pale; distribution: Panama	*Tytthus panamensis* Carvalho & Southwood
–	Antennal segment II fuscous; hemelytra dark smoky brown, especially through middle; hind femora variable	18
18	Hind femur infuscated on apical third (Figs 48, 49); distribution: coastal eastern North America	*Tytthus vagus* (Knight)
–	Hind femur uniformly pale yellow; distribution: Rio de Janeiro, Brazil	*juturnaiba* Carvalho & Wallerstein
19	Head black, contrasting with largely pale orange pronotum and scutellum, pronotum sometimes becoming infuscated	20
–	Head, pronotum, and scutellum uniformly fuscous to black	22
20	Tibiae, femora, and pronotum uniformly pale orange (Figs 45–47); distribution: Arizona and New Mexico	*Tytthus uniformis* Henry, sp. n.
–	Tibiae fuscous to black; femora orange to orange brown, often infuscated or streaked with red; pronotum pale orange to brown, usually invaded with fuscous or dark brown	21
21	Pronotum brown to orange brown, calli darker brown, with a narrow, transverse, pale or white fascia across anterior margin; hemelytron uniformly pale, smoky brown (Figs 17, 18); distribution: Arizona	*Tytthus insperatus* (Knight)
–	Pronotum orange to orange brown, but lacking a narrow, transverse, pale fascia across anterior margin; hemelytron pale with inner half of clavus, apical half of corium, and apex of cuneus brown (Figs 7, 8); distribution: Florida to Texas	*Tytthus balli* (Knight)
22	Hemelytra pale or translucent white; hind femora pale yellow (Figs 15, 16); distribution: Arizona	*Tytthus fuscicornis* Henry, sp. n.
–	Hemelytra largely dark brown to fuscous; hind femora fuscous to black	23
23	Hemelytra with basal third of corium, basal half of clavus, and most of cuneus pale or white; antennal segment I black; all femora fuscous to black; only macropterous male known (Fig. 11); distribution: Colombia	*Tytthus columbiensis* Carvalho
–	Hemelytra with basal third of corium and basal half of clavus pale; cuneus, when present, uniformly dark brown; antennal segment I pale yellow; only hind femur fuscous, front and middle femora yellowish; brachypters common (Figs 50–52); distribution: eastern United States	*Tytthus wheeleri* Henry, sp. n.

### Tytthus alboornatus

(Knight)

http://species-id.net/wiki/Tytthus_alboornatus

Figs 2–4 109–112

Cyrtorhinus alboornatus Knight 1931: 172 (orig. descrip.).
Tytthus alboornatus : Carvalho and Southwood 1955: 27 (descrip., n. comb.); Carvalho 1958: 156 (cat.); Henry and Wheeler 1988: 457 (cat.); Schuh 1995 (cat.); Denno 1983: 702 (note); Hoffman 2000: 24 (note, distr.); Hines et al. 2005: 264 (note).

#### Diagnosis.

This species is distinguished by the small size, usually brachypterous hemelytra, overall dark brown coloration, with the basal third to half of the corium and clavus, and cuneus (or in brachypters the posterior margin of the corium) pale or white, the pale yellowish brown antennal segment I, the mostly dark brown femora and pale yellowish-brown tibiae and tarsi. Macropterous and brachypterous forms are known for both sexes.

This species is most similar in size and coloration to *Tytthus wheeleri*, sp. n. In *Tytthus alboornatus*, antennal segment I is pale yellowish brown and the posterior margin of each hemelytron in brachypters or the cuneus in macropters is pale or white, whereas in *Tytthus wheeleri*, antennal segment I is dark brown and the posterior margin of each hemelytron in brachypters and or cuneus in macropters is uniformly dark brown.

#### Description.

**Figures 2–12.**
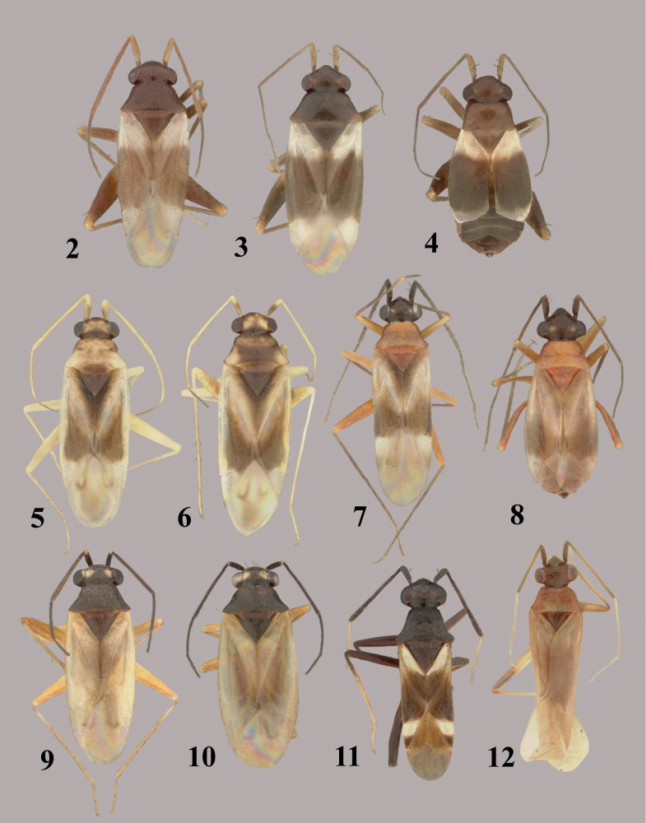
*Tytthus* spp. **2** *Tytthus alboornatus*, macropterous ♂ (holotype: USA, Jacksonville, Florida, 23 July 1926, E. D. Ball, USNM) **3** *Tytthus alboornatus*, macropterous ♀ (USA, South Carolina, Colleton Co., 1.1 km W of Bennetts Point, 3 May 2003, A. G. Wheeler, Jr., USNM) **4** *Tytthus alboornatus*, brachypterous ♀ (USA, South Carolina, Colleton Co., Bear Island Wildlife Management Area, 3 May 2003, A. G. Wheeler, Jr., USNM) **5** *Tytthus amazonicus*, macropterous ♂ (Brazil, Amazonas, Reserva Ducke, 25 km NNE of Manaus, 26 July 1973, R. T. Schuh, AMNH) **6** *Tytthus amazonicus*, macropterous ♀ (Brazil, Amazonas, Reserva Ducke, 25 km NNE of Manaus, 26 July 1973, R. T. Schuh, AMNH) **7** *Tytthus balli*, macropterous ♂ (USA, Texas, Refugio Co., 6 mi. N Bayside, 20 Apr. 1983, T. J. Henry & A. G. Wheeler, Jr., USNM) **8** *Tytthus balli*, brachypterous ♀ (USA, Florida, Jacksonville, 23 July 1926, E. D. Ball, USNM) **9** *Tytthus chinensis*, macropterous ♂ (Guam, Asan Village, Asan River at Rt. 1, 8 June 2008, R. S. Zack, USNM) **10** *Tytthus chinensis*, macropterous ♀ (Guam, Asan Village, Asan River at Rt. 1, 8 June 2008, R. S. Zack, USNM) **11** *Tytthus columbiensis*, macropterous ♂ (holotype: Colombia, Valle de l Cauca, Palmira, 25 Oct. 1958, G. Bravo, USNM) **12** *Tytthus entrerianus*, macropterous ♂ (paratype: Argentina: Entre Rios, Concordia, Apr. 1939, L Carpintero, USNM).

*Macropterous male* (n = 1, plus holotype in parentheses) (Fig. 2): Length to apex of hemelytron 2.21 mm (2.24 mm), length to base of cuneus 1.57 mm (1.66 mm), width across hemelytra 0.72 mm (0.77 mm). *Head*: Length 0.72 mm (0.26 mm), width across eyes 0.51 mm (0.54 mm), interocular width 0.30 mm (0.30 mm). *Labium*: Length [embedded in glue] (0.94 mm). *Antenna*: Segment I length 0.29 mm (0.37 mm), II 0.88 mm (1.06 mm), III 0.59 mm (0.72 mm), IV 0.48 mm (missing). *Pronotum*: Length 0.32 mm (0.35 mm), basal width 0.45 (0.72 mm).

*Brachypterous male* (n = 5): Length to apex of abdomen 1.34–1.57 mm, length to base of hemelytron (cuneus and membrane absent) 1.15–1.41 mm, width across hemelytra 0.58–0.62 mm. *Head*: Length 0.22–0.26 mm, width across eyes 0.50–0.59 mm, interocular width 0.29–0.30 mm. *Labium*: Length 0.83–0.86 mm. *Antenna*: Segment I length 0.29–0.30 mm, II 0.80–0.86 mm, III 0.53–0.64 mm, IV 0.45–0.61 mm. *Pronotum*: Length 0.24–0.29 mm, basal width 0.54–0.56 mm.

*Coloration*: *Head*: Brown to dark brown, with a small, vague, pale spot near inner margin of each eye; eyes dark brown to reddish brown. *Labium*: Yellowish brown, apex of segment IV darker brown. *Antenna*: Segment I pale yellowish brown; segments II–IV yellowish brown, sometimes becoming slightly darker brown. *Pronotum*: Uniformly shiny brown to very dark brown or fuscous. *Mesoscutum*: Hidden under base of pronotum in brachypters, narrowly exposed in macropters. *Scutellum*: Brown to dark brown, with apex pale. *Hemelytron*: Broadly dark brown, with basal one fourth and narrow apex or cuneus (in macropter) pale or white; membrane on only macropter fully developed, smokey brown. *Ventral surface*: Thoracic pleural areas brown to dark brown, ventral surface sometime paler yellowish brown; abdomen dark brown to fuscous, especially laterally, ventral area sometimes paler yellowish brown. *Ostiolar evaporative area*: Dark brown. *Legs*: Coxae pale yellowish brown to whitish, with bases dark brown; femora dark brown, pale yellowish brown at bases and apices; tibiae, tarsi, and claws pale yellowish brown.

*Structure, texture, and vestiture*: *Head*: Shiny, impunctate; broader than long, rounded anteriorly, truncate basally; set with short, recumbent, nearly bristlelike setae on vertex and frons. *Labium*: Extending beyond metacoxae to second or third abdominal segment. *Pronotum*: Shiny, impunctate, nearly rectangular, wider than long, anterior angles rounded, base truncate, calli indistinct, not differentiated from discal surface, only slightly raised and rounded; set with scattered, recumbent, brown to nearly black setae. *Scutellum*: Equilateral, impunctate, with a few scattered, short, recumbent setae. *Hemelytron*: Macropter with fully developed cuneus and membrane, including two closed cells or areoles; all other specimens (except one macropter) brachypterous (staphylinoid), with clavus fused (and claval suture absent) with corium, cuneal fracture and cuneus absent, and membrane absent or rarely with only a remnant narrow strip along truncate posterior margin, extending from about abdominal tergite IV to nearly to apex of abdomen; set with evenly scattered, short, recumbent brown setae.

*Male genitalia*: *Left paramere* (Fig. 109): Mitt-shaped; right arm long, stout; left arm short, apically acute. *Right paramere* (Fig. 110): Oval. *Endosoma* (Fig. 111): Slender, S-shaped, apex pointed. *Phallotheca* (Fig. 112): Relatively slender, apically acute.

*Macropterous female* (n = 1) (Fig. 3): Length to apex of hemelytron 2.45 mm, length to base of cuneus 1.85 mm, width across hemelytra 0.93 mm. *Head*: Length 0.32 mm, width across eyes 0.58 mm, interocular width 0.32 mm. *Labium*: Length 0.99 mm. *Antenna*: Segment I length 0.32 mm, II 0.91 mm, III 0.69 mm, IV 0.67 mm. *Pronotum*: Length 0.37 mm, basal width 0.86 mm.

*Brachypterous “minor” female* [see discussion below] (n = 10): Length to apex of abdomen 1.44–1.79 mm, length to apex of hemelytra 1.34–1.60 mm, width across hemelytra 0.58–0.80 mm. *Head*: Length 0.26 mm, width across eyes 0.43–0.53 mm, interocular width 0.29–0.30 mm. *Labium*: Length 0.80–0.91 mm. *Antenna*: Segment I length 0.26–0.27 mm, II 0.74–0.80 mm, III 0.53–0.59 mm, IV 0.48–0.61 mm. *Pronotum*: Length 0.27–0.29 mm, basal width 0.50–0.56 mm.

*Brachypterous “major” female* (n = 2) (Fig. 4): Length to apex of abdomen 2.02–2.30 mm, length to apex of hemelyra 1.60–1.86 mm, width across hemelytra 0.93-0.99 mm. *Head*: Length 0.27–0.30 mm, width across eyes 0.56–0.61 mm, interocular width 0.34–0.35 mm. *Labium*: Length 0.91–0.96 mm. *Antenna*: Segment I 0.30–0.32 length mm, II 0.90–0.96 mm, III 0.64 mm, IV 0.50 mm. *Pronotum*: Length 0.29–0.34 mm, basal width 0.59–0.64 mm.

**Figures 13–23.**
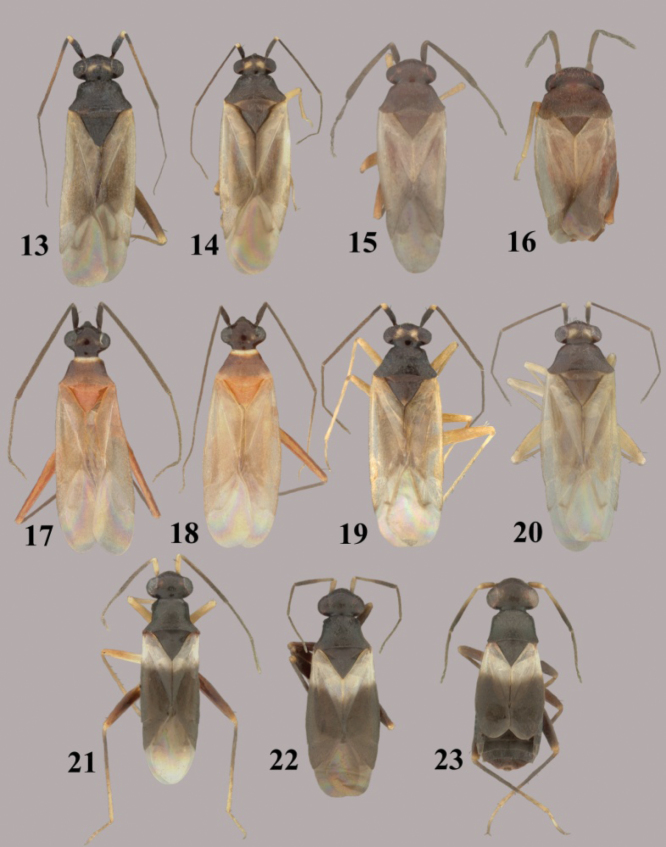
*Tytthus* spp. **13** *Tytthus femoralis*, macropterous ♂ (holotype, Ecuador, Man., Bahiade Caraquez, 10 May 1975, A. B. Gurney, USNM) **14** *Tytthus femoralis*, macropterous ♀ (Peru, Huan. Tingo Maria, 19-24 April 1969, P. & P. Spangler, USNM) **15** *Tytthus fuscicornis*, macropterous ♂ (holotype, USA, New Mexico, Grant Co., Gila Natl. Forest, N of Silver City, 12 May 2008, A. G. Wheeler, Jr., USNM) **16** *Tytthus fuscicornis*, macropterous ♀ (paratype, USA, New Mexico, Grant Co., Gila Natl. Forest, N of Silver City, 12 May 2008, A. G. Wheeler, Jr., USNM) **17** *Tytthus insperatus*, macropterous ♂ (holotype, USA, Arizona, Tuscon, 7 June 1924, A. A. Nichol, USNM) **18** *Tytthus insperatus*, macropterous ♀ **19** *Tytthus mexicanus*, macropterous, ♂ (Mexico, Sinaloa, Choix, 5 Aug. 1968, I. A. Sears, R. C. Gardner, & C. S., UCD) **20** *Tytthus mexicanus*, macropterous ♀ (Mexico, Baja Calif. Sur, 12.2 mi. SE of San Perdito, 8 Oct 1981, F. Andrews & D. Faulkner, SDNM) **21** *Tytthus montanus*, macropterous ♂ (Utah, Cache Co., Rt. 89 nr Franklin Basin Rd., 17-18 July 2001, T.J.Henry & A.G. Wheeler, Jr., USNM) **22** *Tytthus montanus*, macropterous ♀ (Big Lake, Arizona, Apache Natl. For., 12-14 Aug. 1967, L. A. Kelton, CNC) **23** *Tytthus montanus*, brachypterous ♀ (Big Lake, Arizona, Apache Natl. For., 12-14 Aug. 1967, L. A. Kelton, CNC).

**Figures 24–34.**
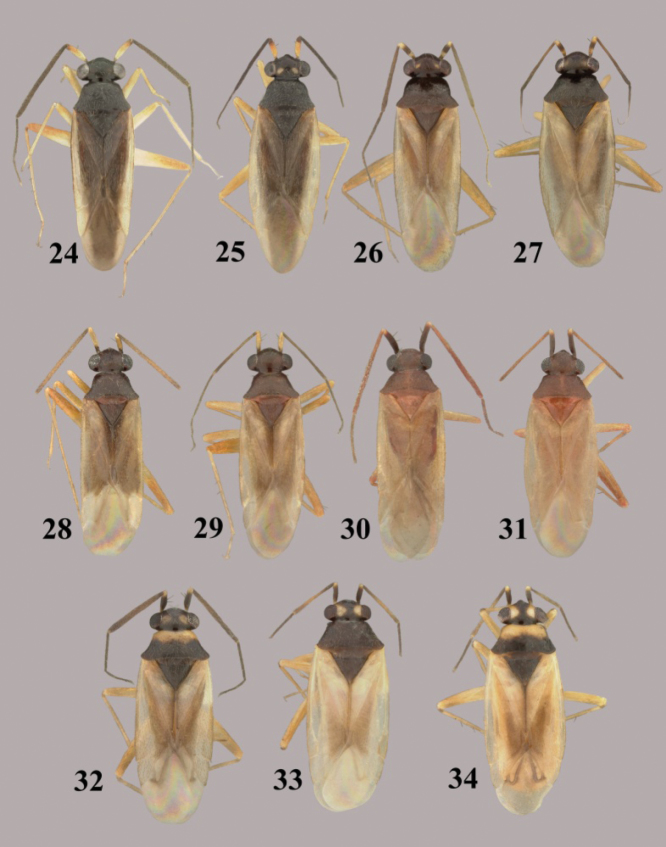
*Tytthus* spp. **24** *Tytthus mundulus*, macropterous ♂ (Hawaii, Oahu, Ewa Beach, 22 Nov. 1982, no coll. data, USNM) **25** *Tytthus mundulus*, macropterous ♀ (Hawaii, Oahu, Barbers Point, Mar. 1960, E. J. Ford, USNM) **26** *Tytthus neotropicalis*, macropterous ♂ (Brazil, Minas Gerais, Viçosa, 13 Oct.-1 Nov. 1985, T.J. Henry & P.S.F. Fiuza, USNM) **27** *Tytthus neotropicalis*, macropterous ♀ (Brazil, Minas Gerais, Viçosa, 13 Oct.-1 Nov. 1985, T.J. Henry & P.S.F. Fiuza, USNM) **28** *Tytthus pallidus*, macropterous ♂ (holotype, Panama, El Real, 19 Mar. 1953, F. S. Blanton, USNM) **29** *Tytthus pallidus* macropterous ♀ (Brazil, Amazonas, Reserva Ducke, 25 km NNE Manaus, 26 July 1973, R. T. Schuh, AMNH) **30** *Tytthus panamensis*, macropterous ♂ (Paratype: Panama, Canal Zone, Corozal, 14 Apr12, A. Busch, USNM) **31** *Tytthus panamensis*, macropterous ♀ (Panama, Canal Zone, Ft. Gulick, 21 Aug. 1952, F. S. Blanton, USNM) **32** *Tytthus parviceps*, macropterous ♂ (pale pronotum) (Bermuda, Paget Par., Paget Marsh, 14-22 July 1988, M. R. Wilson & D. J. Hilburn, USNM) **33** *Tytthus parviceps*, macropterous ♀ (dark pronotum) (Florida, Sebring, 25-31 July, C. T. Parsons, USNM) **34** *Tytthus parviceps*, macropterous ♀ (pale pronotum) (Florida, Deerfield, 26 July 1948, R. H. Beamer, KU).

**Figures 35–44.**
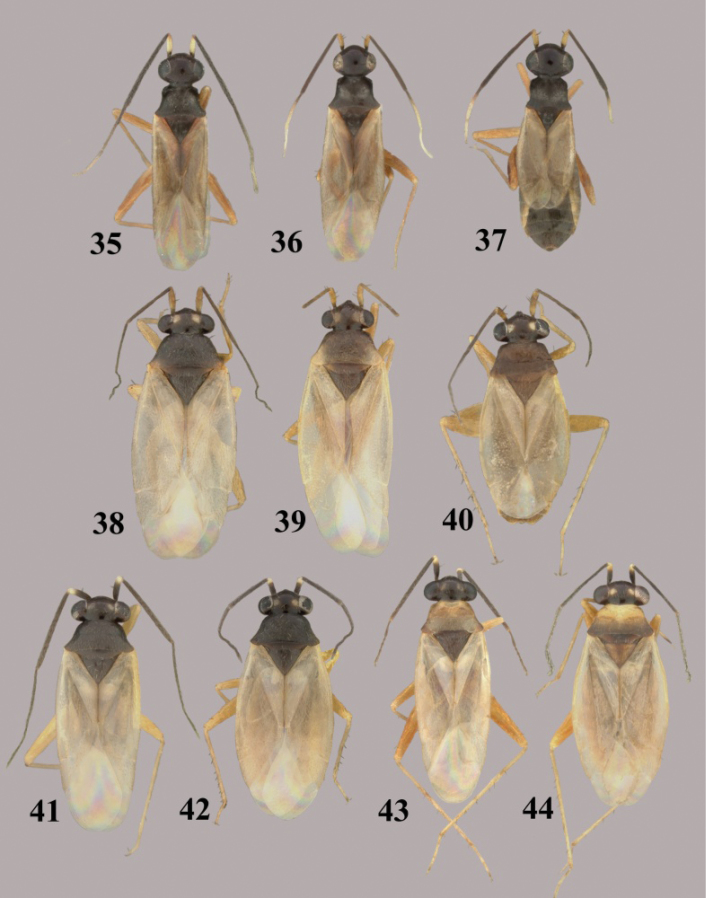
*Tytthus* spp. **35** *Tytthus piceus*, macropterous ♂ (South Carolina, Pickens Co., SC Botanical Gardens, Clemson, 28 Aug. 2004, A. G. Wheeler, Jr., USNM) **36** *Tytthus piceus*, macropterous ♀ (South Carolina, Pickens Co., SC Botanical Garden, Clemson, 28 Aug. 2004, A. G. Wheeler, Jr., USNM) **37** *Tytthus piceus*, brachypterous ♀ (South Carolina, Pickens Co., SC Botanical Gardens, Clemson, 28 Aug. 2004, A. G. Wheeler, Jr., USNM) **38** *Tytthus pubescens*, macropterous ♂ (British Colombia, Pouce Coupe, 18 Aug 1982, L. A. Kelton, CNC) **39** *Tytthus pubescens*, macropterous ♀ (Colorado, [2024], Spicer’s North Park, 18 July 1896, C. F. Baker, USNM) **40** *Tytthus pubescens*, brachpterous ♀ (Alberta, Banff-Jasper Hwy., Jasper Natl. Pk, 26 and 28 Aug 1970, L. A. Kelton, CNC) **41** *Tytthus pygmaeus*, macropterous ♂ (dark pronotum) (Alberta, McMurray, 11 July 1993, W. J. Brown, CNC) **42** *Tytthus pygmaeus*, macropterous ♀ (dark pronotum) (To be added) **43** *Tytthus pygmaeus*, macropterous ♂ (pale pronotum) (England, Wolvercote, Oxford, 14 July 1968, G. G. E Scudder, CNC) **44** *Tytthus pygmaeus* ♀ (pale pronotum) (same locality as for ♂).

**Figures 45–54.**
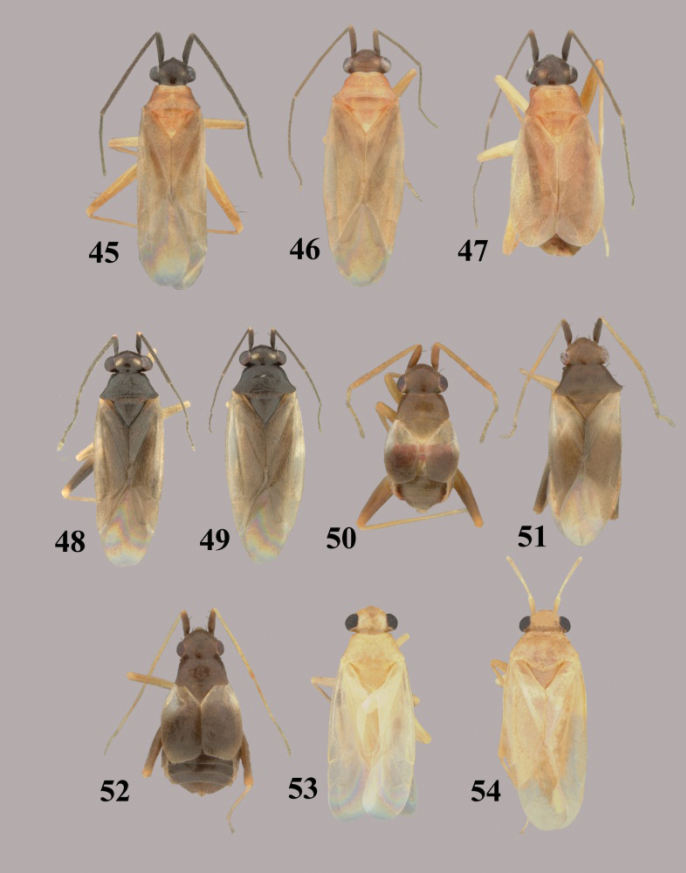
*Tytthus* spp. **45** *Tytthus uniformis*, macropterous ♂ (Arizona, Santa Cruz Co., Audubon Res. Ranch, SE of Elgin, 12-14 May 2004, A. G. Wheeler, Jr., USNM) **46** *Tytthus uniformis*, macropterous ♀ (Arizona, Cochise Co., Huachuca Mtns., Ash Canyon Rd., 0.5 mi. W of Hwy 92, 17 Aug. 1992, N. McFarland, USNM) **47** *Tytthus uniformis*, brachypterous ♀ (Arizona, Santa Cruz Co., Audubon Res. Ranch, SE of Elgin, 12-14 May 2004, A. G. Wheeler, Jr., USNM) **48** *Tytthus vagus*, macropterous ♂ (Florida, Duval Co., 1 km W of Dunn Creek, S. of Eastport, 3 Apr. 2004, A. G. Wheeler, Jr., USNM) **49** *Tytthus vagus*, macropterous ♀ (USA: Florida, Duval Co., south of Eastport, 3 Apr. 2004, A. G. Wheeler, Jr., USNM) **50** *Tytthus wheeleri*, staphylinoid ♂ (Texas, Gillespie Co., Rt16, 15 km NE of Fredericksburg, 27 May 2001, A. G. Wheeler, Jr., USNM) **51** *Tytthus wheeleri*, macropterous ♀ **52** *Tytthus wheeleri*, staphylinoid ♀ (South Carolina, Pickens Co., 2 mi. W of Chiefland, 16 Mar. 1999, A. G. Wheeler, Jr., USNM) **53** *Tytthus zwaluwenburgi*, macropterous ♂ (Canton Island, 20 Nov. 1940, R. Danner, BPBM) **54** *Tytthus zwaluwneburgi*, macropterous ♀ (Baker Island, 18 April 1935, E. H. Bryan, BPBM).

#### Hosts.

Taken by A. G. Wheeler on inland saltgrass, *Distichlis spicata* (L.) Green [Poeaceae]; saltmeadow cordgrass, *Spartina patens* (Ait.) Muhl.; and sand cordgrass, *Spartina bakeri* Merr. [Poeaceae]. Denno (in litt, 2005) informed me that *Tytthus alboornatus* was extremely abundant only in *Spartina patens* in Tuckerton, New Jersey, but not in *Spartina alterniflora* where *Tytthus vagus* was restricted. Denno (1983) and Hines et al. (2005) reported that *Tytthus alboornatus* preys on eggs of the delphacids *Tumidagena minuta* McDermott and possibly *Delphacodes detecta* (Van Duzee) in *Spartina patens*.

#### Distribution.

Previously known only from Florida, New Jersey, New York, and Virginia (Henry and Wheeler 1988, Hoffman 2000). New U. S. state records are Connecticut, Louisiana, South Carolina, and Texas. This distribution indicates that *Tytthus alboornatus* should occur in all coastal states from at least New England to Texas.

#### Discussion.

*Tytthus alboornatus* (Figs 2–4) is one of the smallest species in the genus, second in size only to the similar-appearing *Tytthus wheeleri* (Figs 50–52). Like *Tytthus wheeleri*, macropterous forms of this species are extremely uncommon or rare. Of the more than 50 specimens studied, I have found only two macropterous males, including the holotype (from Florida) and three macropterous females (from Connecticut and South Carolina).

In addition, populations include what I call “minor” and “major” females. Along the Connecticut and New Jersey coasts, specimens are much smaller (shorter and more slender), whereas farther south in South Carolina and Florida several specimens are considerably larger, with more well-developed pronota and hemelytra. Two macropterous females from Connecticut also were smaller than the macropter from South Carolina. This size difference may simply be due to more harsh or crowded conditions, with a more limited food supply versus smaller populations with more plentiful prey available, rather than their north/south distributions. More work is needed to better understand the factors that influence size.

#### Type material examined.

**Holotype** ♂ (00162206) (USNM): **UNITED STATES: Florida: *Duval Co*.:** Jacksonville, 30.33194°N, 81.65583°W, 23 Jul 1926, E. D. Ball.

#### Other specimens examined.

**UNITED STATES:** **Connecticut:** ***New London Co*.:** Stonington Township, Barn Island, 41.335°N, 71.906°W, 11 Aug 1971, R.T. and J. C. Schuh, 9 ♂♂ (00095349, 00165938 - 00165945), 11 ♀♀ (00095350, 00165946 - 00165955) (AMNH), 1 ♂ ( 00138679) (USNM) (all brachypterous, except two macropterous ♀♀). ***Tolland Co*.:** Mansfield Center, 41.76528°N, 72.19861°W, 84 m, 01 Aug 1956, J. A. Slater, 1 ♀ (00166063) (AMNH) **(brachypterous)**. **Florida:** ***Brevard Co*.:** Titusville, 28.61194°N, 80.80778°W, 29 Apr 1952, collector unknown, 1 ♂ (00161887) (USNM) (macropterous). ***Duval Co*.:** CR-105, 1 km W. of Dunn Creek, S. of Eastport, 03 Apr 2004, A. G. Wheeler, Jr., ex *Spartina patens* (Poaceae), 1 ♀ (00161882) (USNM) (brachypterous). ***Miami-Dade Co*.:** Biscayne Bay, 25.5747°N, 80.3112°W, 1700, Mrs. A.T. Slosson, 2 ♂♂ (00165956, 00165957) (AMNH) (brachypterous)**. Louisiana:** Tangipahoa Parish: Hammond, 22 June 1948, E. L. Todd, 1 ♂, 1 ♀ (AMNH) (macropterous). **New Jersey:** ***Ocean Co*.:** 2 mi. E. of Manahawkin off Stafford Ave., 20 Jul 1976, collector unknown, *Distichlis spicata* (Poaceae), 5 ♂♂ (00161870, 00161877 - 00161878, 00161880, 00161886), 2 ♀♀ (00161879, 00161881) (USNM) (all brachypterous). Tuckerton, 39.60305°N, 74.34027°W, 4 m, 01 Aug 2002, R.F. Denno & D. Lewis, *Spartina patens* (Poaceae), 7 ♂♂ (00161865 - 00161868, 00161872, 00161874 - 00161875), 4 ♀♀ (00161869, 00161871, 00161873, 00161876) (USNM) (all brachypterous). **South Carolina:** ***Colleton Co*.:** Bear Island Wildlife Management Area, near Mary’s House Pond, 03 May 2003, A. G. Wheeler, Jr., ex *Spartina patens* (Poaceae), 1 ♀ (00161883) (USNM (brachypterous). CR-26, 0.3 km N. of Ashepoo River, N. of Bennetts Pt., 03 May 2003, A. G. Wheeler, Jr., ex *Spartina bakeri* (Poaceae), 1 ♀ (00161885) (USNM) (brachypterous). CR-26, 1.1 km W. of Bennetts Point, 04 May 2003, A. G. Wheeler, Jr., *Spartina bakeri* (Poaceae), 1 ♂ (macropterous), 1 ♀ (00161889) (brachypterous) (USNM). **Texas:** ***Jefferson Co*.:** J.D. Murphree Wildlife Management Area, near Lost Lake, 29.78111°N, 93.97333°W, 26 May 1994, R. Vogtsberger, taken by mosquito dipper cup, 1 ♂ (00161888) (USNM) (brachypterous). **Virginia**: ***Mecklenburg Co*.**: Elm Hill WMA, Clyde’s Pond, 11-29 May 1995, VMNH survey, 4 ♂♂ (VMNH). ***City of Virginia Beach***: Dam Neck Naval Base, dune DF site, 25 June 1991, K. A. Buhlmann, 1 ♂ (VMNH).

### Tytthus amazonicus

Carvalho

http://species-id.net/wiki/Tytthus_amazonicus

Figs 5, 6 113–116

Tytthus amazonicus Carvalho 1983: 191 (orig. descrip.); Schuh 1995: 248 (cat.).

#### Diagnosis.

This species, distinguished by the pale antennae and legs, mostly pale dorsum, with only the frons and basal margin of the head, scutellum, inner margin of the clavus, and the distal third of the corium brown, cannot be easily confused with any other species of the genus. Only macropters are known.

*Tytthus amazonicus* keys to *Tytthus zwaluwenbergi* because of the pale tibiae, antennae, and head but its relationship with this central Pacific species almost certainly is only superficial. *Tytthus amazonicus* is readily separated by the pale head and pronotum invaded with dark brown, the largely pale hemelytra with the inner half of each clavus and the apical half of each corium dark brown, whereas *Tytthus zwaluwenbergi* is uniformly pale yellowish brown.

#### Description.

*Male* (n = 5) (Fig. 5): Length to apex of hemelytron 2.83–2.93 mm, length to base of cuneus 2.00–2.10 mm, width across hemelytra 0.86–0.93 mm. *Head*: Length 0.34–0.37 mm, width across eyes 0.62–0.64 mm, interocular width 0.29–0.30 mm. *Labium*: Length 1.22–1.28 mm. *Antenna*: Segment I length 0.37–0.38 mm, II 1.00–1.08 mm, III 0.56–0.66 mm, IV 0.48–0.53 mm. *Pronotum*: Length 0.35–0.38 mm, basal width 0.83–0.88 mm.

*Coloration*: *Head*: Pale yellowish brown dorsally, dark brown ventrally, on frons, and narrowly across basal margin; eyes dark brown to reddish brown. *Labium*: Pale yellowish brown. *Antenna*: Segments I–III uniformly pale yellow to yellowish brown, segment I sometimes darker brown through middle with apex and base pale, segment IV slightly darker brown. *Pronotum*: Mostly pale yellowish brown, collar and narrow posterior margin around calli darker brown, entire discal area darker brown on some specimens. *Mesoscutum*: Pale yellowish brown, tinged with darker brown through middle. *Scutellum*: Brown. *Hemelytron*: Predominantly pale or pale yellowish brown, with inner margin of clavus and apical half of corium darker brown; cuneus uniformly pale or pale yellowish brown; membrane translucent brown, veins darker brown. *Ostiolar evaporative area*: Dark brown. *Ventral surface*: Thoracic area dark brown; abdomen brown to yellowish brown, darker brown along lateral margins and genital capsule. Legs: Coxae pale yellow, meso- and metacoxae brown at bases; remainder of legs pale yellow.

*Structure, texture, and vestiture*: *Head*: Wider than long, impunctate, frons with a glaucus sheen; buccula relatively wide; set with short recumbent setae on frons and a few longer, more erect setae on vertex. *Labium*: Extending beyond metacoxae to base of abdomen; segment I extending to middle of procoxae. *Pronotum*: Impunctate, shiny; trapeziform, anterior angles rounded, lateral margins weakly concave, basal angles flared wider, basal margin concave; calli weakly swollen, delimited posteriorly by a shallow impressed line; set with evenly scattered, short, recumbent setae. *Mesoscutum*: Broadly exposed. *Scutellum*: Impunctate, equilateral, with scattered, short, recumbent setae. *Hemelytron*: Macropterous, impunctate, shiny lateral margins subparallel, cuneus longer than wide at base, membrane with two areoles, extending well beyond abdomen.

*Male genitalia*: *Left paramere* (Fig. 113): Mitt-shaped, with a long, broad right arm and shorter, more slender left arm. *Right paramere* (Fig. 114): Elongate oval. *Endosoma* (Fig. 115): S-shaped, with apex rounded. *Phallotheca* (Fig. 116): Relatively slender, apically acute.

*Female* (n = 4) (Fig. 6): Length to apex of hemelytron 2.98–3.14 mm, length to base of cuneus 2.18–2.30 mm, width across hemelytra 0.91–1.07 mm. *Head*: Length 0.34–0.35 mm, width across eyes 0.58–0.59 mm, interocular width 0.30-0.32 mm. *Labium*: Length 1.23–1.33 mm. *Antenna*: Segment I 0.29–0.30 length mm, II 0.85–0.91 mm, III 0.56–0.61 mm, IV mm. *Pronotum*: Length 0.42–0.43 mm, basal width 0.83–0.96 mm.

#### Host.

Unknown. Most specimens taken at light.

#### Distribution.

Described and previously known only from Amazonas, Brazil. Peru is a new country record.

#### Specimens examined.

**BRAZIL:** **Amazonas:** Reserva Ducke, 25 km NNE of Manaus, 120 m, 26 Jul 1973, R.T. Schuh, 2 ♂♂ (00165859, 00165860), 2 ♀♀ (00165857, 00165858) (AMNH), 1 ♂ (00162159), 1 ♀ (00162158) (USNM). On Amazon River, Jul 1900, Boquaert, 1 ♂ (00161884) (USNM). R. Madeira to St. Antonio, May 74, 3 ♀♀ (BNHM). **Para:** ***Santarem Co*.:** Taperinha, 11 Jun 1927 - 20 Jun 1927, Zerny, 1 ♂ (00161615) (USNM). **PERU:** **Junin:** Satipo, 11.2667°S, 74.6833°W, Jul 1940 - Aug 1940, P. Paprzycki, 1 ♀ (00161613) (USNM). **Loreto:** Lake Yarinacocha, 10 km NW of Pucallpa, 150 m, 10 Dec 1971, R. T. Schuh, 1 ♂ (00165855), 1 ♀ (00165856) (USNM).

### Tytthus balli

(Knight)

http://species-id.net/wiki/Tytthus_balli

Figs 7, 8 117–120

Cyrtorhinus balli Knight 1931: 171 (orig. descrip.).
Tytthus balli : Carvalho and Southwood 1955: 30 (descrip., n. comb.); Carvalho 1958: 157 (cat.); Henry and Wheeler 1988: 457 (cat.); Schuh 1995: 248 (cat.).

#### Diagnosis.

This species is distinguished by the black head and antennae, orange-brown pronotum often infuscated laterally, orange-brown scutellum, pallid hemelytra infuscated along inner margin of clavus and distal third of corium, orange-brown femora, and dark brown to fuscous tibiae. All known males are macropterous; both macropterous and brachypterous females occur.

*Tytthus balli* is very similar to *Tytthus insperatus* and *Tytthus uniformis* based on the black head and antennal segments I and II, and the orange to brownish-orange pronotum and femora. From *Tytthus insperatus*, it is distinguished by the uniformly browish-orange pronotum lacking a pale anterior margin and the infuscated apical half of each corium. From *Tytthus uniformis* it is distinguished by the fuscous tibiae and infuscated hemelytra.

#### Description.

*Macropterous male* (n = 5, plus holotype in parentheses) (Fig. 7): Length to apex of hemelytron 2.58–2.73 mm (2.68 mm), length to base of cuneus 1.85–1.88 mm (1.96 mm), width across hemelytra 0.70–0.83 mm (0.80 mm). *Head*: Length 0.34–0.35 mm (0.37 mm), width across eye 0.58–0.59 mm (0.59 mm), interocular width 0.30–0.32 (0.30 mm). *Labium*: Length 1.05–1.07 mm (1.02 mm). *Antenna*: Segment I length 0.30–0.32 mm (0.29 mm), II 0.98–1.07 mm (1.02 mm), III 0.69–0.85 mm (0.80 mm), IV 0.48–0.51 mm (missing). *Pronotum*: Length 0.30-0.32 mm (0.34 mm), basal width 0.72–0.74 mm (0.69 mm).

*Coloration*: *Head*: Uniformly shiny black, with a distinct, yellow spot on inner interocular area bordering each eye. *Labium*: Yellowish brown, apex of segment IV fuscous. *Antenna*: Uniformly black. *Pronotum*: Uniformly orange to darker brownish orange, often becoming infuscated laterally and around calli. *Mesoscutum*: Orange to brownish orange. *Scutellum*: Dark orange or brownish orange to dark brown. *Hemelytron*: Pale or whitish on basal half and narrow apical margin of corium, narrow outer margin of clavus, and basal half of cuneus, dark brown on most of clavus, apical half of corium, and apex of cuneus; membrane translucent brown, veins slightly dark brown. *Ostiolar evaporative area*: Brownish orange to fuscous. *Ventral surface*: Thoracic area orange to dark brownish orange; abdomen brownish orange to dark brown, often becoming fuscous laterally, genital capsule dark brown to fuscous. *Legs*: Coxae pale or whitish, orange to dark brownish orange basally; femora orange to brownish orange; tibiae brown on palest specimens, especially protibia, to dark brown or black on darker individuals; tarsi and claws brown to dark brown.

*Structure, texture, and vestiture*: *Head*: Broader than long, shiny, impunctate; sparsely set with short, recumbent setae; buccula relative wide, tapering posteriorly. *Labium*: Extending to apices of metacoxae or base of abdomen; segment I extending only to prosternum. Pronotum: Impunctate, shiny; trapeziform, anterior angles rounded, lateral margins weakly concave, basal angles flared wider, basal margin concave; calli weakly swollen, delimited posteriorly by a shallow impressed line; set with evenly scattered, short, recumbent setae. *Mesoscutum*: Broadly exposed. *Scutellum*: Impunctate, equilateral, width a few scattered, recumbent setae. *Hemelytron*: Macropterous, impunctate, shiny, lateral margins subparallel, cuneus longer than wide at base, membrane with two areoles, extending well beyond abdomen.

*Male genitalia*: *Left paramere* (Fig. 117): Mitt-shaped; right arm long, broad; left arm shorter, more slender. *Right paramere* (Fig. 118): Oval. *Endosoma* (Fig. 119): C-shaped. *Phallotheca* (Fig. 120): Slender, relatively straight, apically acute.

*Macropterous female* (n = 2): Length to apex of hemelytron 2.90–3.07 mm, length to base of cuneus 2.08–2.28 mm, width across hemelytra 0.83–0.93 mm. *Head*: Length 0.34–0.35 mm, width across eyes 0.58–0.61 mm, interocular width 0.32–0.34 mm. *Labium*: Length 1.17–1.22 mm. *Antenna*: Segment I length 0.27–0.32 mm, II 1.02–1.1.18 mm, III 0.77–0.80 mm, IV 0.48–0.54 mm. *Pronotum*: Length 0.32–0.34 mm, basal width 0.74–0.78 mm. Similar to males in color and shape.

*Brachypterous female* (n = 4) (Fig. 8): Length to apex of abdomen 2.20–2.38 mm, length to base of cuneus 1.88–1.93 mm, width across hemelytra 0.86–0.90 mm. *Head*: Length 0.37–0.40 mm, width across eyes 0.58–0.59 mm, interocular width 0.34–0.35 mm. *Labium*: Length 1.17-1.20 mm. *Antenna*: Segment I length 0.27–0.29 mm, II 1.01–1.02 mm, III 0.77–0.80 mm, IV 0.48–0.50 mm. *Pronotum*: Length 0.30–0.32 mm, basal width 0.67–0.69 mm. Similar to males in color, differing in having the cuneus reduced (basal width subequal to length) and the membrane shortened (with the veins absent or indistinct), extending only to the middle of the last abdominal tergite.

#### Hosts.

Previously recorded only from imported roses (Carvalho and Southwood 1955), which undoubtedly is an incidental record. A. G. Wheeler and I have swept specimens from a salt marsh area containing mixed herbaceous vegetation and *Spartina* sp. [Poaceae].

#### Distribution.

This species was described from Jacksonville, Florida (Knight 1931), and later reported from an unspecified Mexican locality based on a specimen intercepted at Brownsville, Texas (Carvalho and Southwood 1955). The specimens listed below from Clay, Hidalgo, and Refugio counties represent the first authentic Texas records.

#### Type material examined.

**Holotype** ♂ (00162202) (USNM) (macropterous): **UNITED STATES: *Duval Co*.:** Jacksonville, 30.33194°N, 81.65583°W, 23 Jul 1926, E. D. Ball. **Paratypes**: **UNITED STATES: Texas: *Duval Co*.:** Jacksonville, 30.33194°N, 81.65583°W, 23 Jul 1926, E. D. Ball, 1 ♂ (00161903) (USNM), 2 ♀♀ (00161912, 00161913), 1 nymph (00161910), 2 ♀♀ (00161914, 00161915) (USNM).**Texas: *Presidio Co*.:** Presidio, 29.56056°N, 104.37167°W, 28 Sep 1929, W.L. Owens, 1 ♂ (00167070) (CNC) (macropterous), 1 ♀ (00161911) (USNM) (brachypterous).

#### Other specimens examined.

**UNITED STATES:** **Florida:** **Texas:** ***Cameron Co*.:** Brownsville, 25.90139°N, 97.49722°W, 28 Mar 1945, collector unknown, 1 ♀ (00161917) (USNM) (macropterous). Brownsville, Veteran’s Bridge (Cargo Lot), 27 Jul 2011, S. Guzman, 1 ♂ (USNM). ***Chambers Co*.**: Anahuac, 29.77278°N, 94.6825°W, 08 Oct 1918, H. S. Barber, 1 ♂ (00161904) (USNM). ***Clay Co*.:** 6 mi NE of Bexar at Red River (at state line), 34.13611°N, 98.13055°W, 20 Sep 2009, G. F. and J. F. Hevel, 1 ♂ (00161909) (USNM) (macropterous). ***Hidalgo Co*.:** Hidalgo County, 26.1°N, 98.26278°W, 19 May 1930, J. C. Gaines, Paratype, 1 ♀ (00161916) (USNM). ***Refugio Co*.:** 6 mi. N. Bayside, Rt. 136, 20 Apr 1983, T. J. Henry and A. G. Wheeler, Jr, 4 ♂♂ (00161905 - 00161908) (USNM) (macropterous).

### Tytthus chinensis

(Stål)

http://species-id.net/wiki/Tytthus_chinensis

Figs 9, 10 55–62 121–124

Capsus chinensis Stål 1860: 258 (orig. descrip.); Atkinson 1890: 107 (cat.).
Cyrtorhinus chinensis : Reuter 1903: 22 (descrip.); Hsiao 1942: 255 (key).
Cyrtorhinus annulicollis Poppius 1915: 65 (orig. descrip.). Synonymized by Carvalho and Southwood 1955: 20.
Cyrtorhinus elongatus Poppius 1915: 65 (orig. descrip.). Synonymized by Carvalho and Southwood 1955: 20.
Cyrtorhinus riveti Cheesman 1927: 94 (orig. descrip.); Knight 1935: 204 (list); Usinger 1939: 270 (key, host), 1946: 79 (note), 1951: 4 (key). Synonymized by Carvalho and Southwood 1955: 20.
Tytthus chinensis : Carvalho and Southwood 1955: 19 (key, descrip.) Carvalho 1958: 157 (cat.); Tomokuni et al. 1993: 307 (note, photo); Schuh 1995: 248 (cat.); Cassis and Gross 1995: 204 (cat.); Kerzhner and Josifov 1999: 441 (cat.); Yasunaga et al. 2001: 182 (note, photo).
Tytthus koreanus Josifov and Kerzhner 1972: 171 (orig. descrip.); Kerzhner 1988: 840 (key); Schuh 1995: 249 (cat.); Kerzhner and Josifov 1999: 441 (cat.); Kwon et al. 2001: 184 (cat.). syn. n.

#### Diagnosis.

This species is very similar to *Tytthus parviceps* in general size and in sharing the dark base of each tibia, dark antennae with the apex and base of segment I pale, and short, erect, brushlike setae on antennal segment II. *Tytthus chinensis* almost always has a uniformly black pronotum (Figs 9, 10) or the pronotum with only weak indications of yellow around the calli, and the endosoma (Fig. 123) is C-shaped, whereas *Tytthus parviceps* has the anterior one third to half of the pronotum around the calli almost always extensively pale yellow (Figs 32, 34) and the endosoma appears more distinctly S-shaped (Fig. 170). All known specimens of both species are macropterous.

Three other exclusively New World species, *Tytthus entrerianus*, *Tytthus femoralis*, and *Tytthus mexicanus*, also have fuscous “knees,” a character that distinguishes them from all other species of the genus, except. *Tytthus chinensis* and *Tytthus parviceps* as noted above and in the key. All three, however, lack the brushlike setae on antennal segment II and the apical one third to two thirds of the hind femora of these species are infuscated.

#### Description.

*Male* (n = 15) (Fig. 9): Length to apex of hemelytron 2.18–2.60 mm, length to base of cuneus 1.65–1.88 mm, width across hemelytra 0.79–0.93 mm. *Head*: Length 0.27–0.29 mm, width across eyes 0.56–0.58 mm, interocular width 0.29–0.30 mm. *Labium*: Length 0.72–0.94 mm. *Antenna*: Segment I length 0.24–0.26 mm, II 0.78–0.82 mm, III 0.43–0.51 mm, IV 0.30–0.32 mm. *Pronotum*: Length 0.29–0.32 mm, basal width 0.69–0.80 mm.

*Coloration*: Uniformly fuscous to black, with a large, yellow, interocular spot touching inner margin of each eye, spots nearly contiguous in some individuals; eyes fuscous to dark reddish brown. *Labium*: Uniformly pale yellow, except of brown apical half of segment IV. *Antenna*: Segment I black, with base and apex narrowly pale yellow; segments II–IV uniformly fuscous to black. *Pronotum*: Usually uniformly black, anterior third around calli frequently weakly tinged with yellow, and less often entire anterior third yellow; posterior half uniformly fuscous to black. *Mesoscutum*: Uniformly yellowish brown to fuscous. *Scutellum*: Uniformly fuscous to black. *Hemelytron*: Uniformly translucent yellow to whitish. *Ostiolar evaporative area*: Yellowish, with central area of auricle invaded with fuscous, sometimes entirely fuscous. *Ventral surface*: Anterior half of proacetabula often yellow, propleura, pro- and mesosterna, and metapleura fuscous; abdomen varying from largely yellowish, with only genital capsule fuscous or black to largely fuscous with only ventral area pale. *Legs*: Uniformly generally yellow; procoxae uniformly yellow, meso-and metacoxae dark brown at bases, yellow beyond; femora yellow, often tinged with orange; tibiae yellow, bases broadly fuscous; tarsi and claws yellowish.

*Structure, texture, and vestiture*: *Head*: Weakly shiny, impunctate; buccula slender, extending posteriorly, ending near level with hind margin of eye; thickly set with short to relatively long semierect setae, especially on frons. *Labium*: Extending to apices of meso- or bases of metacoxae; segment I extending beyond base of head to xyphyus just before procoxae. *Antenna*: Segment I set with short, recumbent setae and two, long, subapical, bristlelike setae; segment II thickly set with short, recumbent setae, intermixed with row of longer, erect setae (Fig. 59) along ventral surface. *Pronotum*: Anterior angles rounded; lateral margins weakly concave, gradually widening to rounded posterior angles; posterior margin weakly sinuate. *Mesoscutum*: Weakly shiny, impunctate; set with a few scattered, semierect setae. *Scutellum*: Weakly shiny, impunctate; equilateral; set with a few scattered recumbent and semierect setae. *Hemelytron*: Macropterous, cuneus and membrane fully developed, extending posteriorly well beyond apex of abdomen; evenly set with relatively long, recumbent setae.

*Male genitalia*: *Left paramere* (Fig. 121): Mitt-shaped; right arm long, brown; left arm short, spinelike. *Right paramere* (Fig. 122): Oval. *Endosoma* (Fig. 123): C-shaped, apex blunt. *Phallotheca* (Fig. 124): Relatively slender, strongly bent (C-shaped), apically acute.

*Female* (n = 15) (Fig. 10): Length to apex of hemelytron 2.50–2.78 mm, length to base of cuneus 1.86–2.00 mm, width across hemelytra 0.90–0.96 mm. *Head*: Length 0.29–0.30 mm, width across eyes 0.60–0.62 mm, interocular width 0.30–0.32 mm. *Labium*: Length 0.90–0.94 mm. *Antenna*: Segment I length 0.26–0.27 mm, II 0.45–0.46 mm, III 0.26–0.27 mm, IV 0.29–0.30 mm. *Pronotum*: Length 0.29–0.30 mm, basal width 0.80–0.85 mm.

#### Hosts.

Usinger (1939) reported *Tytthus chinensis* (as *Cyrtorhinus riveti*) feeding on the eggs of *Sogata ochrias* (Kirkaldy) [Delphacidae] on *Sporobolus virginicus* (L.) Kunth [Poaceae] and *Nilaparvata lugens* (Stål) [Delphacidae] on rice (*Oryza* sp.) in Guam. He also recorded this species from Bermudagrass, *Cynodon dactylon* (L.) Pers., in Samoa, and *Tradescantia* sp. [Commelinaceae] in Tahiti.

**Figures 55–62.**
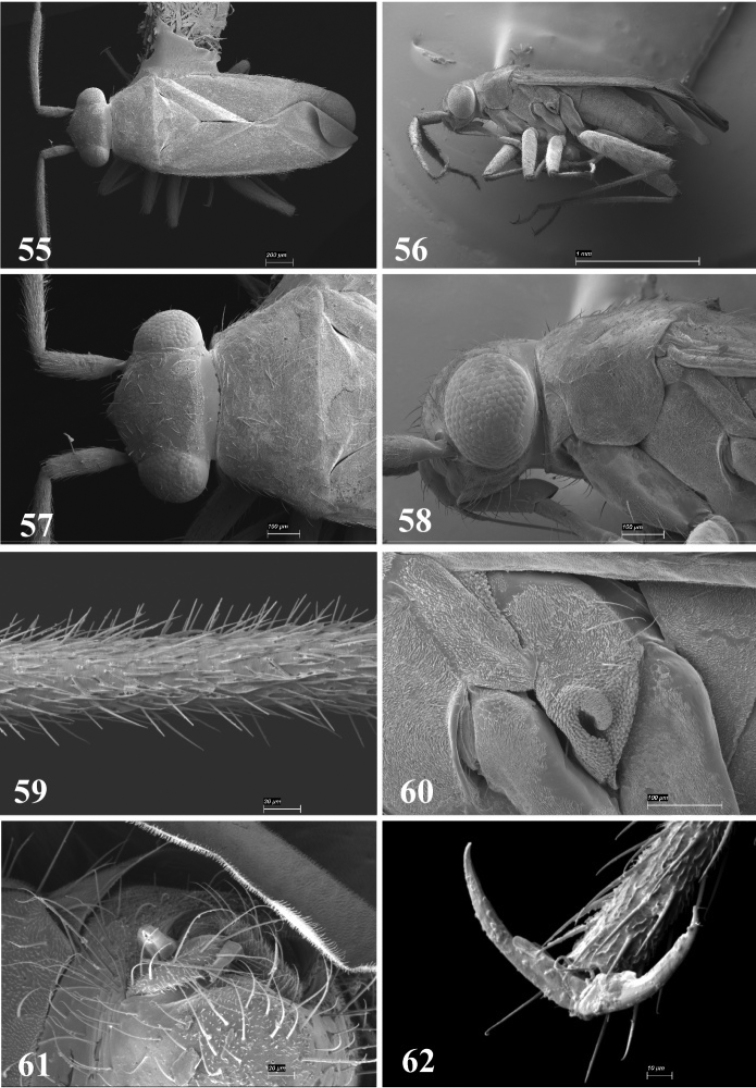
SEM photomicrographs of *Tytthus chinensis*, male **55** dorsal habitus **56** lateral habitus **57** head and pronotum, dorsal aspect **58** head and pronotum, lateral aspect **59** antennal segment II **60** ostiolar evaporative area **61** genital capsule, caudal aspect **62** claw.

**Figures 63–70.**
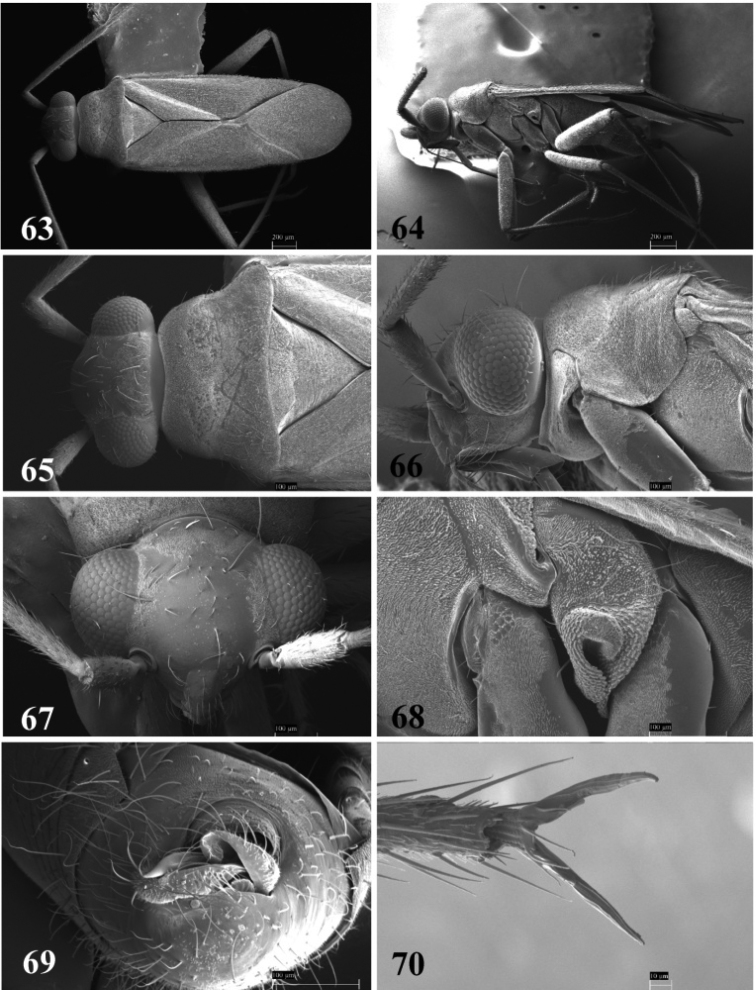
SEM photomicrographs of *Tytthus neotropicalis*, male **63** dorsal habitus **64** lateral habitus **65** head and pronotum, dorsal aspect **66** head and pronotum, lateral aspect **67** head, frontal aspect **68** ostiolar evaporative area **69** genital capsule, caudal aspect **70** claw.

**Figures 71–78.**
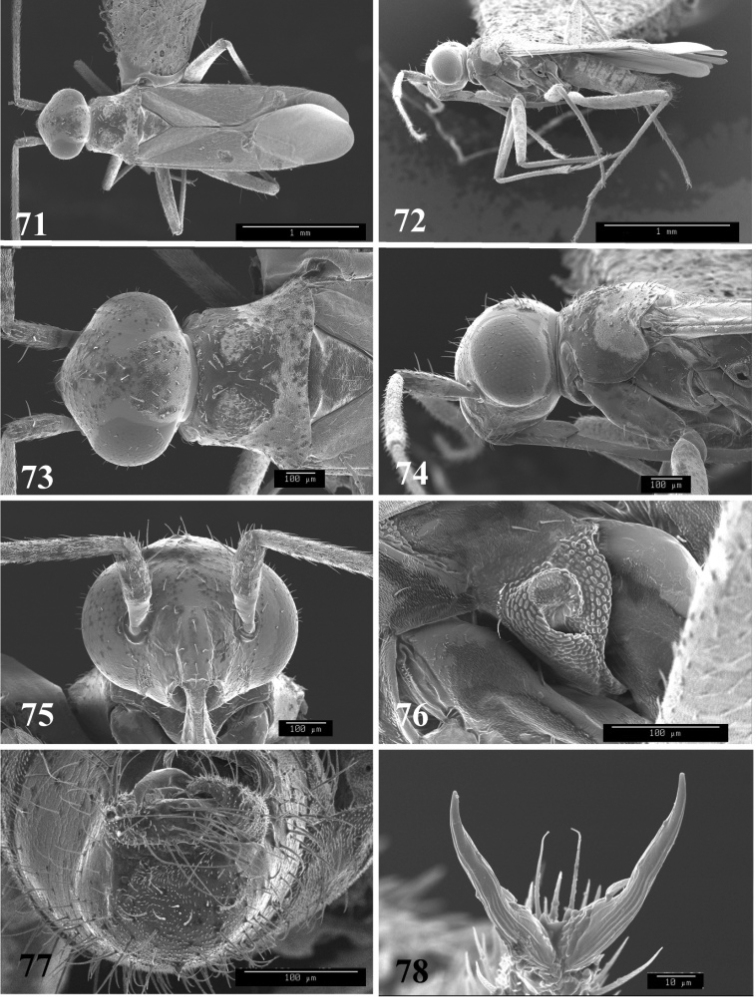
SEM photomicrographs of *Tytthus piceus*, male **71** dorsal habitus **72** lateral habitus **73** head and pronotum, dorsal aspect **74** head and pronotum, lateral aspect **75** head, anterior aspect **76** ostiolar evaporative area **77** genital capsule, caudal aspect **78** claw.

**Figures 79–86.**
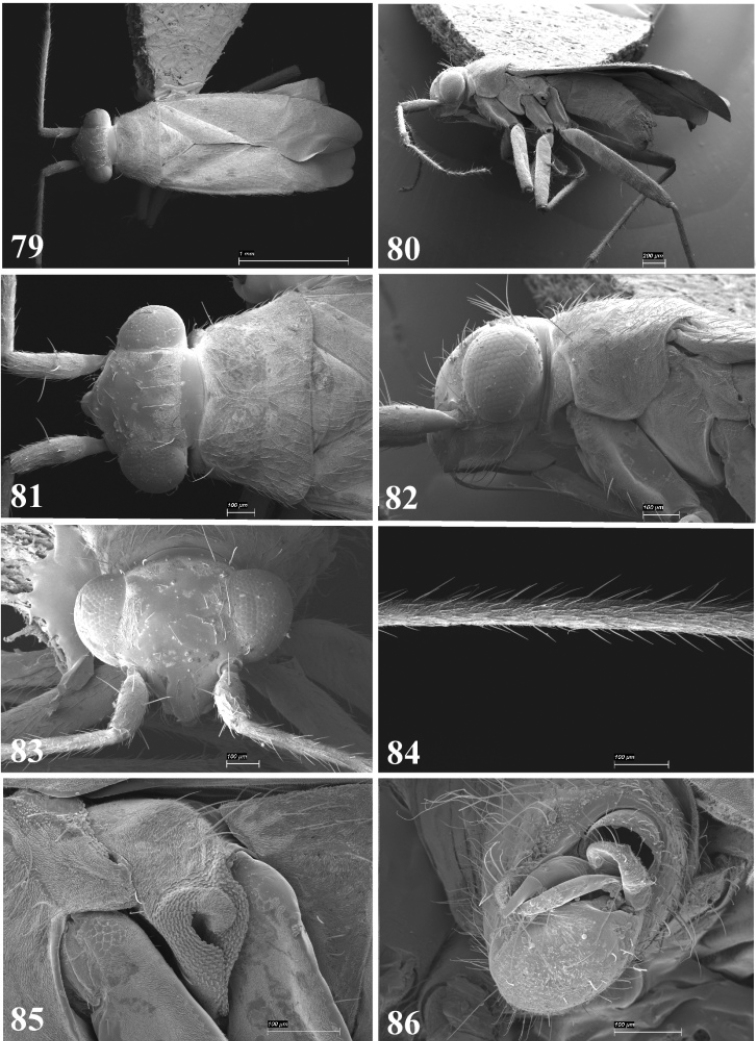
SEM photomicrographs of *Tytthus pubescens*, male **79** dorsal habitus **80** lateral habitus **81** head and pronotum, dorsal aspect **82** head and pronotum, lateral aspect **83** head, anterior aspect **84** antennal segment II **85** ostiolar evaporative area **86** genital capsule, caudal aspect.

**Figures 87–94.**
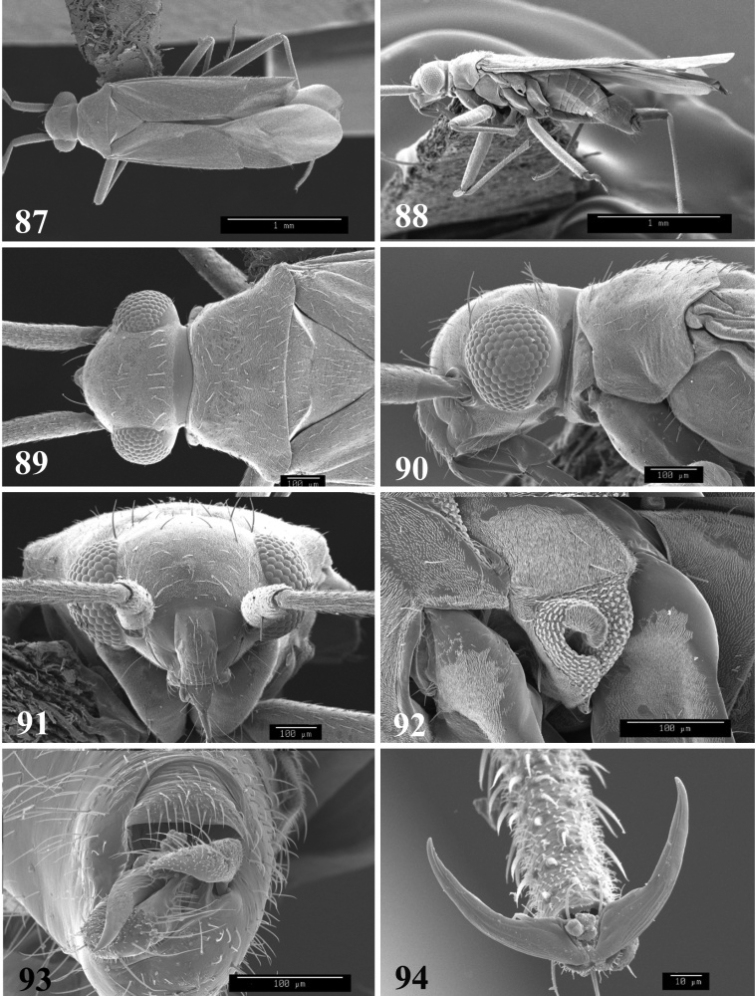
SEM photomicrographs of *Tytthus uniformis*, male **87** dorsal habitus **88** lateral habitus **89** head and pronotum, dorsal aspect **90** head and pronotum, lateral aspect **91** head, anterior aspect **92** ostiolar evaporative area **93** genital capsule, caudal aspect **94** claw.

**Figures 95–100.**
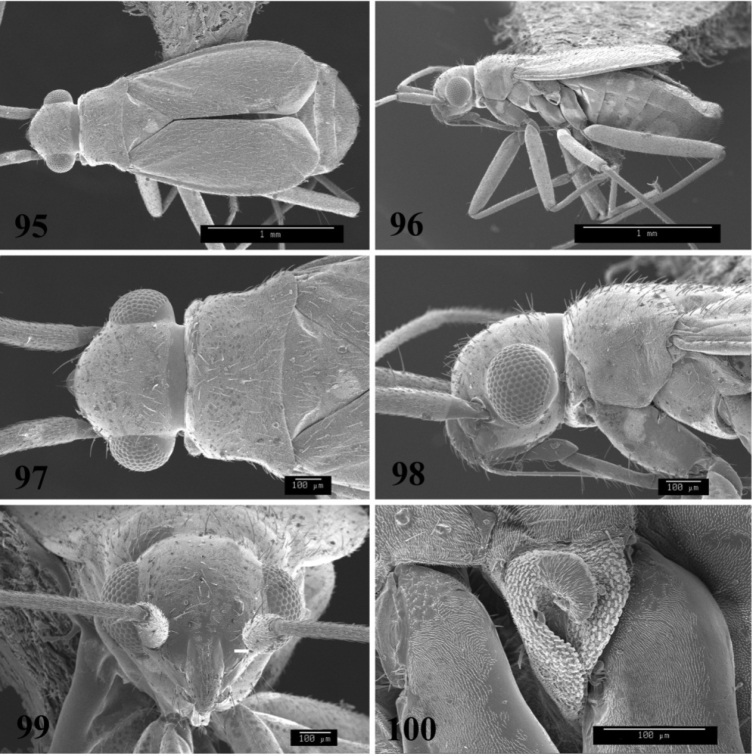
SEM photomicrographs of *Tytthus uniformis*, female **95** dorsal habitus **96** lateral habitus **97** head and pronotum, dorsal aspect **98** head and pronotum, lateral aspect **99** head, anterior aspect **100** ostiolar evaporative area.

**Figures 101–108.**
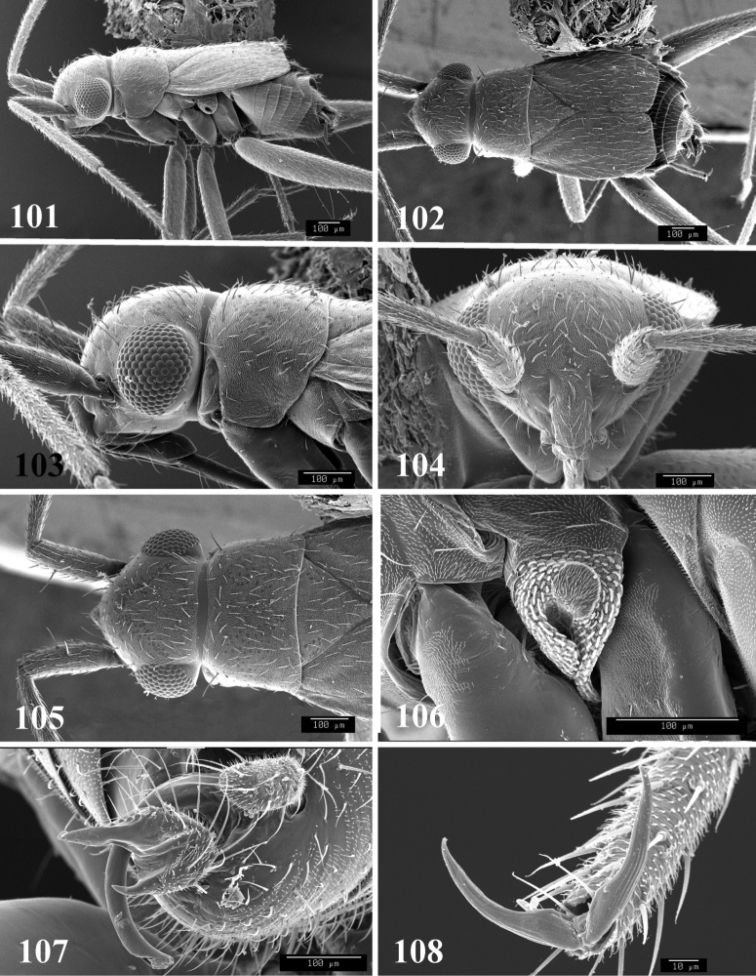
SEM photomicrographs of *Tytthus wheeleri*, male **101** dorsal habitus **102** lateral habitus **103** head and pronotum, dorsal aspect **104** head and pronotum, lateral aspect **105** head, anterior aspect **106** ostiolar evaporative area **107** genital capsule, caudal aspect **108** claw.

**Figures 109–116.**
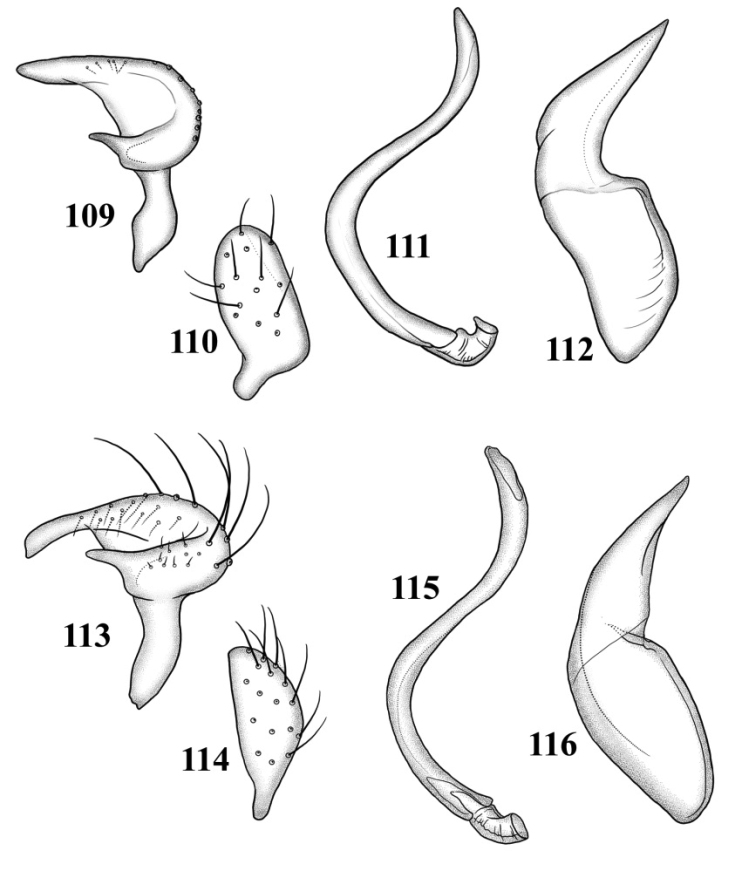
Male genitalia **109–112** *Tytthus alboornatus* **109** left paramere **110** right paramere **111** endosoma **112** phallotheca 113–116 *Tytthus amazonicus* **113** left paramere **114** right paramere **115** endosoma **116** phallotheca.

**Figures 117–124.**
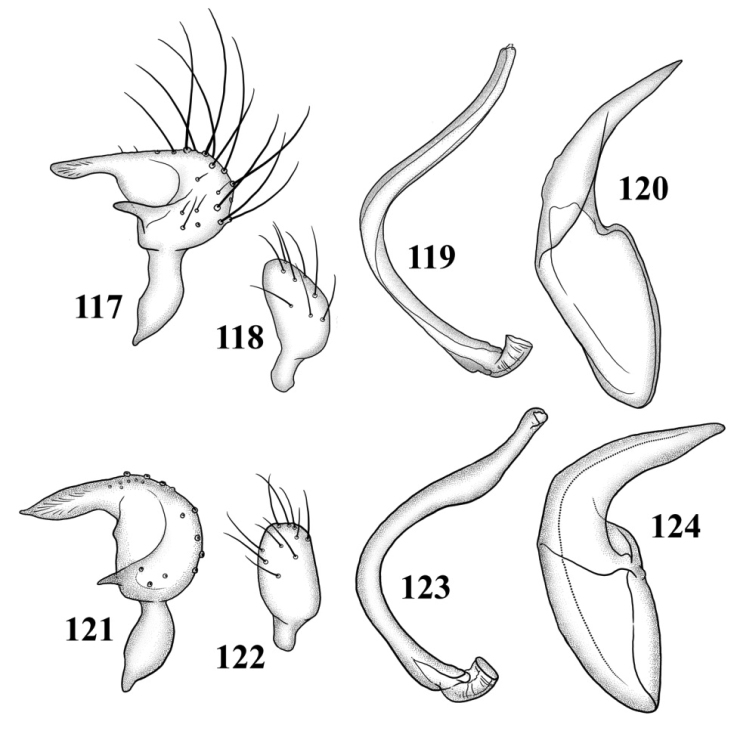
Male genitalia **117–120** *Tytthus balli* **117** left paramere **118** right paramere **119** endosoma **120** phallotheca **121–124** *Tytthus chinensis* **121** left paramere **122** right paramere **123** endosoma **124** phallotheca.

#### Distribution.

This species has been reported from central and southeastern China, Japan, Taiwan, and Australia, India, and the Indo-Pacific Region (Caroline Islands, Childers Island, Cook Islands, Fiji, Gilbert Island, Indonesia, Mariana Islands, Marshall Islands, New Caledonia, New Hebrides, Papua New Guinea, Philippine Islands, Pitcairn Island, Rapa Island, Ryuku Islands, Samoa, Society Islands, Solomon Islands, South Korea, Swain Islands, and Tonga Islands) (Schuh 1984, 1995; Kerzhner and Josifov 1995; Cassis and Gross 1995). Based on specimens in the USNM, most or all records reported by Schuh (1984) from India and Sri Lanka should be applied to *Tytthus parviceps*; Schuh’s (1984) record from Hawaii is based on two specimens intercepted on international commerce at Honolulu (without origin indicated).

Based on specimens examined, I now also have records of this species from Cambodia, Guadalcanal, Guam, Saipan, and Tinian Island.

#### Discussion.

Carvalho and Southwood (1955), after consulting with D. R. Malaise at the Swedish Museum of Natural History in Stockholm, reported that the type of *Capsus chinensis* Stål (1859) must be considered lost. Based on Stål’s original description and study of other types, they concluded that *Cyrtorhinus elongatus* Poppius and *Capsus annulicornis* Poppius (both in Deutsches Entomologisches Institut) and *Capsus riveti* Cheesman (BMNH) are junior synonyms. They characterized *Tytthus chinensis* as “the smallest species of *Tytthus* and is distinguished by its black pronotum and scutellum, the dark bases of the tibia and the small size.” I have studied the type of *Tytthus riveti* and agree that it follows the concept of *Tytthus chinensis* outlined by Carvalho and Southwood (1955) and Carvalho (1956), including a uniformly dark pronotum.

In addition, Kerzhner and Josifov (1995) suggested that *Tytthus koreanus* Josifov and Kerzhner (1972) could be a junior synonym of *Tytthus chinensis*. Although I have not examined type material of *Tytthus koreanus*, the description and figures presented in the original description indicate that this species is based on pale specimens having the calli or anterior third of the pronotum yellow to brownish yellow. Josifov and Kerzhner (1972) did not indicate if they observed specimens with entirely dark pronota. I also have studied a male and two females from South Korea, lent by Dr. Seunghwan Lee (SNU), that agree with the pale color form of *Tytthus chinensis* having only the calli and mesoscutum tinged with dull brownish yellow. As a consequence, I consider T.* koreanus* and the material from Dr. Lee conspecific with that of *Tytthus chinensis*.

Although most material of what I consider to be *Tytthus chinensis* has a uniformly fuscous to black pronotum, varying degrees of yellow are present on the calli and anterior third of the pronotum on some specimens from eastern Asia, throughout the Indo-Pacific, and Australia, which may cause confusion with *Tytthus parviceps* that also has yellow on the anterior area of the pronotum. Nevertheless, I consider all material from eastern Asia (including Korea and Japan), Indonesia, Malaysia, Australia, New Guinea, and the South Pacific Islands *Tytthus chinensis* based on the dark pronotum in most specimens and C-shaped endosoma. However, because of the strong morphological similarity between *T*. “*chinensis*” and *T*. “*parviceps*,” an effort will be made to accumulate fresh material worldwide for conducting a phylogeographic analysis of species limits within this complex using mitochondrial cytochrome oxidase 1 (COI) sequence data in cooperation with colleagues S. Scheffer and M. Lewis (Systematic Entomology Laboratory, ARS, USDA, Beltsville, Maryland, USA).

For the time being, I am restricting the distribution of *Tytthus parviceps* that has the anterior third of the pronotum broadly yellow (but certain specimens within populations can have a uniformly dark pronotum) to southern Asia (India, Srilanka, Pakistan, Vietnam, Thailand), the Middle East, Afrotropical and Neotropical regions, and subtropical United States. A few specimens from the Indo-Pacific (e.g., Guam), Australia, Cambodia, and other eastern Asian countries are externally indistinguishable from typical specimens of *Tytthus parviceps* from Africa and the New World. As noted above, the endosoma of typical *Tytthus chinensis* is C-shaped, whereas this very simple structure in African and New World specimens of *Tytthus parviceps* is usually S-shaped. Until additional collections can be made and additional DNA sequencing can be conducted on these populations, the strongly yellow phenotypes within populations of *Tytthus chinensis* must regarded convergent color forms.

#### Type material examined.

To ensure nomenclatural stability, I designate the following female as the lectotype of *Cyrtorhinus riveti*: Label 1 (circular label with orange ring) “Type”; label 2, “Tahiti: Nr. Papeete[,] Mar.-Apr.[,] Miss Cheesman”; label 3 (handwritten), “ Cyrtorhinus riveti Cheesman”; label 4, “St. George Exp.[,] BM 1925- 235”; label 5 (00085518 (BMNH).”

#### Other specimens examined.

**AUSTRALIA**: **New South Wales:** Deewhy Beach dunes, 28 Jan 1957, W. W. Wirth, 1 ♂ (00162347), 1 ♀ (00167399) (USNM). Narrabri, 25 Jan 1960, M. Nikitin, by light, 1 ♀ (BMNH). **Northern Territory:** Katherina River, 14.5°S, 132.25°E, 12 Nov 1979, Zaytsev, 1 ♂ (00235059) (ZISP). Katherine, 14.467°S, 132.267°E, 17 Nov 1990, W. F. Chamberlain, Light Trap, 1 ♂ (00167391) (USNM). **Queensland:** ***Cape York Islands Co*.:** Banaga, N. Cape York, gum forest, Jan 1958, P.F. Darlington, 1 ♂ (00409566) (KU). Gracemere, 05 Nov 1990, W. F. Chamberlain, Light Trap, 1 ♂ (00167392), 1 ♀ (00167398) (USNM). Mossman, 16.47°S, 145.37172°E, 36 m, 11 Nov 1990, W. F. Chamberlain, 1♀ (00167400), Light Trap, 2 ♂♂ (00167389, 00167393) (USNM). Mt. Berryman Rd., Laidley, 05 Dec 1990, W. F. Chamberlain, Light Trap, 2 ♂♂ (00167395, 00167396) (USNM); 06 Dec 1990, W. F. Chamberlain, Light Trap, 1 ♂ (00167397) (USNM); 07 Dec 1990, W. F. Chamberlain, Light Trap, 1 ♂ (00167394) (USNM). Mt. Isa, 15 Nov 1990, W. F. Chamberlain, Light Trap, 1 ♂ (00167390), 1 ♀ (00167402) (USNM). Tozer Range, Cape York Peninsula, 12.7833°S, 143.2167°E, 122 m, 01 Jul 1948 - 05 Jul 1948, G. M. Tate, 1♀ (00167403) (USNM). **BISMARCK ARCHIPELIGO**: **New Britain**, Keravat, 17 Nov 1957, J. Smart, 1 ♂ (BMNH). **CAMBODIA**: Kampong Seila Dist. National Road, Pk 135, Boeung Trach Village, Picnic Resort, 11–12 Nov 2010, L. T. & R. K. Duval, S. H. Lee, W. Lee, & S. Kim, 1 ♂, 1 ♀ (SNU). **FIJI:** **Viti Levu:** Suva, 02 Mar 1985, Koebele Collection, 1 ♂ (00162346) (USNM). none, 13 Apr 1959, Haw, 2 ♀♀ (00162356, 00162357) (USNM). **GUAM:** **Mariana Is.:** Agana, port, 15 Aug 1945, H. S. Dybas, 1 ♀ (00162351) (USNM). Asan Village, Asan River at Rt. 1, 13.47278°N, 144.7135°E, 08 Jun 2008, R. S. Zack, 1 ♂ (00410393) (WSU). Asan Village, Rt. 1, Asan River outlet at Nino Perdito church, 13.47286°N, 144.71655°E, 13 Mar 2011, R. S. Zack, 38 ♂♂ (00410329 - 00410366), 26 ♀♀ (00410367 - 00410392) (WSU). Mangilao Village, University of Guam campus, Marine Biology Lab area, 13.42856°N, 144.79855°E, 03 Aug 2005, R. S. Zack, 1 ♂ (00410396) (WSU). Mangilao Village, University of Guam campus near Marine Lab dormitory field, 13.42888°N, 144.80083°E, 30 May 2008, R. S. Zack, 1 ♂ (00410395) (WSU). Mongmong-Toto-Maite Village, Pipeline Rd. off of Rt. 33, 13.45678°N, 144.76826°E, 06 Jun 2008, R. S. Zack, 1 ♂ (00410394) (WSU). Pt. Oca, 13.503°N, 144.771°E, 23 Jun 1945, G.E. Bohart and J.L. Gressit, 1 ♀ (00162354) (USNM); 16 Jul 1945, G.E. Bohart and J.L. Gressit, 1 ♂ (00162342), 1 ♀ (00162350) (USNM); 20 Dec 1945, J. L. Gressitt, Light Trap, 1 ♂ (00095351) (AMNH), 1 ♂ (00162341), 2 ♀♀ (00162352, 00162353), Light Trap, 1 ♀ (00162355) (USNM), 2 ♂♂ (00410279, 00410280), 2 ♀♀ (00410277, 00410278) (WSU). **INDONESIA**: Sulawesi: Utara, Dumoga-Bone N. P., 2 ♂♂ (BMNH). **MICRONESIA:** **Ngulu Atoll:** Ngulu Island, 03 Oct 1952, N. L. H. Krauss, 2 ♀♀ (00162358, 00162359) (USNM). **NEW HEBRIDES**: Erromana, Jul 1930, L. E. Cheesman, B. M. 1930–477, 2 ♂♂ (BMNH). Malekula, Malua Bay, May 1929, Miss L. E. Cheesman, B. M. 1929–410, 1 ♀ (BMNH). **NORTHERN MARIANA ISLANDS:** **Rota Island:** (Luta), “Josen Cristina Country” Park, 14.11836°N, 145.1857°E, 14 May 2004, R. S. Zack, 2 ♂♂ (00410401, 00410402) (WSU). (Luta), farm plots/stream area, 14.11851°N, 145.17846°E, 14 May 2004, R. S. Zack, 1 ♂ (00410403) (WSU). **Saipan:** 1 - 2 mi. E. of Tanapag, 10 Jan 1945, H. S. Dybas, 1 ♀ (00162349) (USNM). Saipan: Garapan, sweeping beach, 15.19458°N, 145.71678°E, 20 Jun 2006, R. S. Zack, 3;m (00410398 - 00410400) (WSU). Saipan; Kagman, Kagman Exp. Farm, Northern Marianas College, 15.17503°N, 145.77166°E, 19 Jun 2006, R. S. Zack, 1 ♂ (00410397) (WSU). **Tinian:** Tinian Island, 15.02333°N, 145.63305°E, 11 Jun 1946, H. K. Townes, 3 ♂♂ (00162343 - 00162345) (USNM). **SAMOA:** **Tutuila Island:** Pago Pago, 04 Sep 1923, Swezey and Wilder, 1 ♂ (00162340) (USNM); 20 Sep 1923, Swezey and Wilder, 1 ♀ (00162348) (USNM). Tutuila, 1930, Swezey & Wilder, 1 ♂, 1 ♀ (BMNH). Upolu, Apia, 9 Dec 1923, Swezey & Wilder, on Bermuda grass, 2 ♂♂ (BMNH). **SOLOMON ISLANDS:** **Guadalcanal:** Guadalcanal, 29 Mar 1944, L. J. Lipovsky, 1 ♀ (00409562) (KU); 04 Apr 1944, L. J. Lipovsky, 1 ♀ (00409563) (KU); 19 Apr 1944, L. J. Lipovsky, 1 ♂ (00409564) (KU). Ilu, 8 Apr 1963, M. Mquillan, 2 ♂♂ (BMNH). Kukun, 17 Dec 1962, P. Greenslade, 1 ♂ (BMNH). Nr. Honiara, 25027 Jun 1965, Roy. Soc. Exped. B.M. 1966-1, at light and sweeping around pond, 2 ♀♀ (BMNH). **SOUTH KOREA**: Gyeongsangbuk-do, Is. Ulleung, 30–31 Aug. 2010, L. T. & R. K. Duwal, 1 ♂, 2 ♀♀ (SNU). **THAILAND:** **Bangkok:** Bangkapi at light, 15 Dec 1951, M. E. Griffith, 1 ♂ (00409565) (KU).

### Tytthus columbiensis

Carvalho

http://species-id.net/wiki/Tytthus_columbiensis

Fig. 11

Tytthus columbiensis Carvalho 1984: 202 (orig. descrip.); Schuh 1995: 248 (cat.)

#### Diagnosis.

This distinct species is recognized by the overall shiny fuscous to black body, antennae, and femora, contrasting with the pale or white cuneus, basal area of corium and clavus, antennal segments III and IV, and tibiae.

It is similar to the Nearctic *Tytthus montanus* in the overall dark brown to fuscous dorsum and femora and the basally pale corium, but differs in having most of the cuneus, antennal segments III and IV pale (segments I and II uniformly black) and most of the tibiae (except bases) pale or whitish, whereas in *Tytthus montanus* the cuneus and antennal segment III and IV are uniformly black (segments I pale and II pale on basal third to half) and the tibiae are pale only on the distal halves.

#### Description.

*Holotype male* (Fig. 11): Length to apex of hemelytron ca 3.40 mm (wing separated from body), length to base of cuneus 2.33 mm (wing separated from body), width across hemelytra 1.12 mm. *Head*: Length 0.43 mm, width across eyes 0.64 mm, interocular width 0.32 mm. *Labium*: Length 1.12 mm. *Antenna*: Segment I length 0.32 mm, II 1.04 mm, III 0.64 mm, IV ca 0.51 mm (curled and in glue). *Pronotum*: Length 0.42 mm, basal width 0.82 mm.

*Coloration*: *Head*: Uniformly black, with a vague pale spot on interocular space adjacent to each eye; eyes dark reddish brown. *Labium*: Segments I, II, and apex of IV dark brown; segment III, apex of II, and basal two thirds of IV pale or whitish. *Antenna*: Segments I and II fuscous to black; segment III and IV pale or whitish. *Pronotum*: Uniformly black. *Hemelytron*: Fuscous to black, with basal one fourth of corium and clavus and cuneus, except for apex, pale or white; membrane translucent brown. *Ostiolar evaporative area*: Fuscous to black. *Ventral surface*: Propleura black, ventral areas of thorax dark to reddish brown; abdomen dark reddish brown. *Legs*: Procoxae reddish brown, mesocoxa reddish brown with only apex pale, metacoxa uniformly pale to whitish; femora uniformly black; tibiae pale yellow to whitish, with only basal one fourth of each fuscous to black; tarsi and claws pale yellow to whitish.

*Structure, texture, and vestiture*: *Head*: Shiny, impunctate, width subequal to length, shiny; buccula slender (less than half the width of labial segment I), tapering posteriorly; sparsely set with scattered, short, semierect, dark brown setae on vertex and frons and a few longer, erect setae along posterior margin. *Labium*: Extending to bases of mesocoxae; segment I extending only to base of head. *Pronotum*: Shiny, impunctate; anterior angles rounded, lateral margins weakly concave, posterior anterior angles strongly flared, posterior margin concave; sparsely set with scattered, short, erect and semierect, dark brown setae. *Mesoscutum*: Broadly exposed, impunctate, sparsely set with short, erect, dark brown setae. *Scutellum*: Equilateral, impunctate, sparsely set with short, erect, dark brown setae. *Hemelytron*: Macropterous, impunctate, basal width of cuneus about two thirds the length, membrane fully developed with two areoles; evenly set with short, semierect dark brown setae (pale setae on pale or white areas).

*Male genitalia*: The unique holotype was not dissected.

*Female*: Unknown.

#### Host.

The holotype was taken on *Oryza* sp. (Poaceae).

#### Distribution.

Described and known only from the holotype collected in Colombia.

#### Type material examined.

**Holotype** ♂ (00162207) (USNM): **COLOMBIA:** **Valle del Cauca:** Palmira, 25 Oct. 1958, G. Bravo, Arroz, *Oryza* sp. (Poaceae).

#### Discussion.

The left hemelytron of the holotype is missing, and the right one is glued to the point next to the specimen. Otherwise, the specimen is in reasonably good condition. Carvalho (1984) illustrated the holotype, apparently before the specimen was damaged. Figure 11 depicts the original condition, using Adobe Photoshop to reconstruct the position of both hemelytra.

### Tytthus entrerianus

Carvalho & Carpintero

http://species-id.net/wiki/Tytthus_entrerianus

Figs 12 125–127

Tytthus entrerianus Carvalho and Carpintero 1986: 624 (orig. descrip.); Schuh 1995: 248 (cat.).

#### Diagnosis.

This species is recognized by the dark brown head and antennal segments I and II, the pale brown pronotum with the anterior half darker brown or reddish brown, the translucent, smoky-brown hemelytra, and the mostly pale legs with only the distal thirds of the femora fuscous. All known specimens of this species are macropterous.

This species keys to *Tytthus femoralis* based on the pale tibiae with fuscous knee spots and the apically fuscous hind femora. *Tytthus entrerianus* can be distinguished from *Tytthus femoralis* by the dark brown or fuscous antennal segment II and having only apical third of the hind femur infuscated.

#### Description.

*Male* (n = 1, holotype in parentheses) (composite description based on Carvalho and Carpintero, 1986, and one USNM paratype) (Fig. 12): Length to apex of hemelytron 2.65 mm (3.00 mm), length to base of cuneus 1.80 mm (not given), width across hemelytra 0.74 mm (0.90 mm). *Head*: Length 0.40 mm (0.30 mm), width across eyes 0.54 mm (0.50 mm), interocular width 0.29 mm (0.30 mm). *Labium*: Not visible, imbedded in glue (not given). *Antenna*: Segment I length 0.34 mm (0.30 mm), II 0.94 mm (1.00 mm), III 0.74 mm (0.70 mm), IV 0.56 mm (0.50 mm). *Pronotum*: Length 0.27 mm (0.20 mm), basal width 0.67 mm (0.70 mm).

*Coloration*: *Head*: Uniformly dark brown; pale spot near inner margin of eye absent. *Labium*: Not visible. *Antenna*: Segments I and II dark brown; segment III and IV brownish yellow. *Pronotum*: Anterior half dark brown; posterior half yellowish brown. *Mesoscutum*: Reddish brown. *Scutellum*: Reddish brown. *Hemelytron*: Brown, with basal one fourth and cuneus paler yellowish brown; membrane pale translucent brown. *Ostiolar evaporative area*: Brown. *Ventral surface*: Thorax and abdomen brown to reddish brown, genital capsule dark brown. *Legs*: Pale brownish yellow, apical third of each femur and bases of tibiae (knees) dark brown.

*Structure, texture, and vestiture*: Shiny, impunctate (buccula imbedded in glue); sparsely set with short, semierect setae. *Labium*: Not visible. *Antenna*: Segment I with a few scattered, short, recumbent setae and two long, erect, bristlelike setae at apex; segment II with relatively few, short, recumbent setae. *Pronotum*: Shiny, impunctate; anterior angles rounded; lateral margins straight, gradually widening to posterior angles; posterior margin weaky concave. *Mesoscutum*: Shiny, impunctate; set with only a few semierect setae. *Scutellum*: Shiny, impunctate; set with a few semierect setae. *Hemelytron*: Macropterous, impunctate, subparallel; length of cuneus two times length; membrane entire with two areoles; set with sparsely scattered, recumbent, brown setae.

**Figures 125–131.**
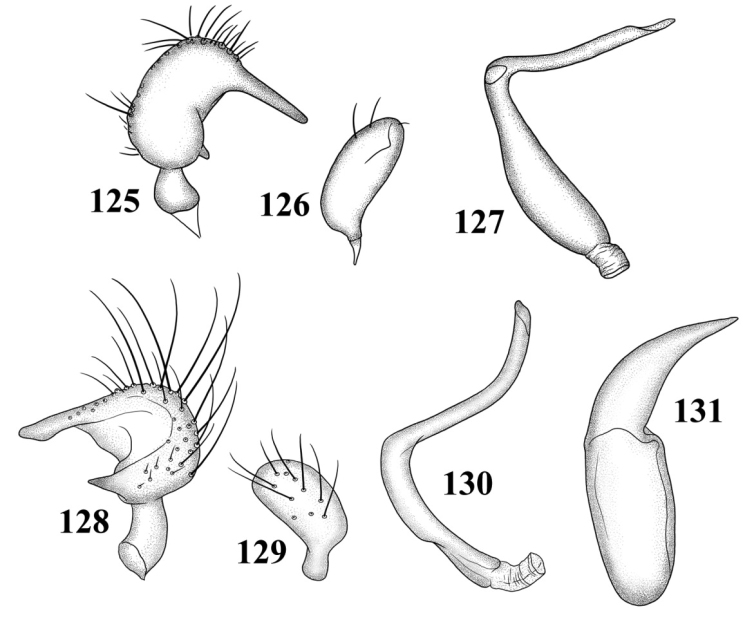
Male genitalia **125–128** *Tytthus entrerianus* **125** left paramere **126** right paramere **127** endosoma **128–131** *Tytthus femoralis* **128** left paramere **129** right paramere **130** endosoma **131** phallotheca.

*Male genitalia* (based on Carvalho and Carpintero’s 1986, figures): *Left paramere* (Fig. 125): Mitt-shaped; right arm long, slender; left arm shorter, spinelike. *Right paramere* (Fig. 126): Oval. *Endosoma* (Fig. 127): C-shaped, with apparent secondary gonopore at middle. *Phallotheca*: Not figured.

*Female*: None examined; described from 5 paratype ♀♀. Carvalho and Carpintero (1986) indicated that the general aspect and dimensions of females were similar to males.

#### Host.

Unknown.

#### Distribution.

Described and known only from Entre Rios Province, Argentina.

#### Type material examined.

**Paratype: ARGENTINA:** **Entre Rios:** Concordia, 31.4°S, 58.033°W, 21 m, Apr 1939 [L. Carpintero], ♂ (00138710) (USNM).

#### Other specimen examined.

**ARGENTINA: Entre Rios:** Concordia, 31.4°S, 58.033°W, Jan 1979, collector unknown, 1 ♂ (00138710) (USNM).

### Tytthus femoralis

Henry sp. n.

urn:lsid:zoobank.org:act:B264F11F-69AD-4E03-8C44-70DE7060E461

http://species-id.net/wiki/Tytthus_femoralis

Figs 13, 14 128–131

#### Diagnosis.

This species is distinguished by the combination of a black head, pronotum, and scutellum; translucent hemelytra usually marked with dark brown through middle of the corium; the fuscous antennal segment I, with only the apex pale; the pale antennal segment II having a fuscous basal band; the fuscous hind femur contrasting with the pale yellow pro- and mesofemora; and the pale tibiae with the bases or “knees” fuscous. All known specimens of this species are macropterous.

*Tytthus femoralis* keys to *Tytthus entrerianus* based on the pale tibiae with knee spots and the apically infuscated hind femora. It can be distinguished from *Tytthus entrerianus* by the pale antennal segment II and the more extensively infuscated hind femur.

#### Description.

*Male* (n =10, plus holotype in parentheses) (Fig. 13): Length to apex of hemelytron 2.56–3.42 mm (2.83 mm), length to base of cuneus 1.90–2.25 mm (2.08 mm), width across hemelytra 0.80–0.99 mm (0.88 mm). *Head*: Length 0.30–0.34 mm (0.35 mm), width across eyes 0.61–0.67 mm (0.63 mm), interocular width 0.27–0.29 mm (0.29 mm). *Labium*: Length 1.20–1.38 mm (1.28 mm). *Antenna*: Segment I length 0.38–0.50 mm (0.38 mm), II 0.94–1.21 mm (1.10 mm), III 0.58–0.72 mm (0.66 mm), IV 0.40–0.59 mm (0.58 mm). *Pronotum*: Length 0.34–0.38 mm (0.35 mm), basal width 0.75–0.90 mm (0.78 mm).

*Coloration*: *Head*: Uniformly fuscous to black, with a large, yellow, interocular spot near inner margin of each eye, spots nearly converging on some specimens; eyes fuscous to dark reddish brown. *Labium*: Pale yellow, with apical half of segment IV brown. *Antenna*: Segment I fuscous to black, with apical one quarter pale or yellowish; segment II pale yellowish brown, with a distinct fuscous band at base and sometimes with apex infuscated; segments III and IV black. *Pronotum*: Uniformly black. *Mesoscutum*: Uniformly black. *Scutellum*: Uniformly black. *Hemelytron*: Translucent, with a dark brown cloud or patch on apical half of cor- ium and inner apical half of clavus ranging from somewhat indistinct to a definite dark pattern (Figs 13, 14); membrane pale translucent brown. *Ostiolar evaporative area*: Dark brown to fuscous, with a yellow patch on posterior edge. *Ventral surface*: Thorax and abdomen uniformly fuscous to black. *Legs*: Coxae pale yellow, with basal thirds to halves dark brown; pro- and meso femora pale yellow, metafemur dark brown to fuscous, with basal third and apex pale yellow; tibiae pale, each with a fuscous base or “knee”; tarsi, and claws pale yellow.

*Structure, texture, and vestiture*: *Head*: Shiny, impunctate; buccula relatively broad, tapering posteriorly, ending near level with middle of eye; set with scattered, semierect setae. *Labium*: Extending to metacoxe or base of abdomen; segment I extending beyond base of head onto xyphus to bases of procoxae. *Antenna*: Segment I sparsely set with recumbent setae and two erect, subapical bristlelike setae; segment II set with only very short, recumbent setae. *Pronotum*: Shiny, impunctate; anterior angles rounded; lateral margins weakly concave, widening at posterior angles; posterior margin weakly sinuate; calli weakly swollen; set with relatively long, recumbent setae. *Mesoscutum*: Weakly shining, impunctate, broadly exposed; set with a few scattered semierect setae. *Scutellum*: Shiny, impunctate; equilateral; set with scattered erect and semierect setae. *Hemelytron*: Macropterous, with cuneus and membrane fully developed, extending well beyond apex of abdomen; evenly set with relatively long, recumbent setae.

*Male genitalia*: *Left paramere* (Fig. 128): Mitt-shaped; right arm long, broad, acute apically; left arm short, apically acute. *Right paramere* (Fig. 129): *Endosoma* (Fig. 130): S-shaped. *Phallotheca* (Fig. 131): Slender, apically acute.

*Female* (n =5) (Fig. 14): Length to apex of hemelytron 2.82–3.52 mm mm, length to base of cuneus 2.05–2.50 mm, width across hemelytra 1.00–1.14 mm. *Head*: Length 0.30–0.35 mm, width across eyes 0.62–0.69 mm, interocular width 0.30–0.32 mm. *Labium*: Length 1.12–1.50 mm. *Antenna*: Segment I length 0.32–0.50 mm, II 0.85–1.17 mm, III 0.53–0.75 mm, IV 0.54–0.59 mm.

*Pronotum*: Length 0.40–0.41 mm, basal width 0.91–0.99 mm.

Similar to males in general appearance and coloration, differing primarily in the overall broader form. The one color exception is that the ventral area of the abdomen in many females is pale yellow, whereas in males the abdomen is always uniformly fuscous to black.

#### Etymology.

The specific epithet A "femoralis" denotes the dark brown to fuscous hind femur in contrast to the uniformly pale pro- and mesofemora.

#### Hosts.

Four specimens from San Carlos, Ecuador, with the label a "Host-Eggs of *Perkinsiella* spp. " Three specimens intercepted on *Musa* sp. certainly represent incidental or sitting records.

#### Distribution.

So far recorded from Bolivia, Brazil, Colombia, Costa Rica, Cuba, Ecuador, Guatemala, Honduras, Jamaica, Mexico, Panama, and Peru.

#### Type material.

**Holotype** ♂ (00161890) (USNM)**: ECUADOR: Manabi:** Bahia de Caraquez (35 kms SE), 10 May 1975, Ashley B. Gurney. **Paratypes: BOLIVIA:** **El Beni:** Capivara on Rio Itenez, approx. 20 km E. of Versalles, 22 Jul 1964, J. K. Bouseman, J. Lussenhop, 1 ♂ (00165937) (AMNH). **BRAZIL:** **Amazonas:** Reserva Ducke, 25 km NNE Manaus, 2.9136°S, 59.9464°W, 120 m, 26 Jul 1973, R. T. Schuh, 1 ♀ (00165831) (AMNH). **COLOMBIA:** **Valle del Cauca:** Jamundi, 3.25833°N, 76.54°W, 13 Jul 1985, F. Garcia, on *Jameo arroz* (Poaceae), 6 ♂♂ (00161854 - 00161859), 2 ♀♀ (00161860, 00161861) (USNM). **COSTA RICA:** **San Jose:** San Jose, 9.9333°N, 84.0833°W, 1147 m, 1932, H. Schmidt, 1 ♂ (00165935), 1 ♀ (00165936) (AMNH). **CUBA:** Origin unknown, intercepted at Baltimore, Maryland by APHIS/PPQ, 26 Nov 1925, in “ship’s light socket”), 1 ♀ (00161862) (USNM). **ECUADOR:** **Chimborazo:** Huigra, 15 Jun 1914, H. S. Parish, 1 ♂ (00161899) (USNM). **El Oro:** Victoria-Arenillas, 150 m, 18 Aug 1977 - 19 Aug 1977, L. Pena G., 1 ♀ (00161931) (USNM). **Guayas:** Duran, 91 m, 23 Jun 1914, H. S. Parish, 2 ♂♂ (00161900, 00161901) (USNM), 2 ♂♂ (CU). Ingenio San Carlos, 07 May 1982, Robert Morey, host–eggs of *Perkinsiella* spp., 1 ♂ (00161898), 3 ♀♀ (00161930, 00161936 - 00161937) (USNM). **Los Rios:** Babahoyo, 21 Jun 1975, Cohen, Langley & Monnig, at blacklight, 1 ♂ (00161934) (USNM). Chone, 0.6833°S, 80.1°W, 110 m, 09 May 1975, Ashley B. Gurney, 2 ♂ (00161929, 00161935) (USNM). **Manabi:** Chone, 0.6833°S, 80.1°W, 110 m, 09 May 1975, Ashley B. Gurney, 2 ♂♂ (00161929, 00161935) (USNM). **Napo:** Baeza (72 Km E), 1280 m, 14 Apr 1977, Elaine R. Hodges, near cut trees and bamboo, 1 ♂ (00161933) (USNM). Origin unknown, intercepted at Long Beach, California, by APHIS/PPQ, 12 May 1997, on *Musa* sp., 1 ♂ (00161891), 1 ♀ (00161932) (USNM). Origin unknown, intercepted at San Diego, California by APHIS/PPQ, 32.71528°N, 117.15639°W, 20 May 2008, on *Musa* sp. (fruit), 1 ♂ (00161902) (USNM). **GUATEMALA:** Locality unknown, 13 Sep 2007, P. Perez, *Musa* sp. (Musaceae), 1 ♀ (00161864) (USNM). **HONDURAS:** **Atlantida:** Lancetilla, 1900 - 1900, M. Bates, Paratype, 2 ♂♂ (00165933, 00165934) (AMNH). **JAMAICA:** **St. Ann Parish:** 5 mi S of St Anns Bay, 05 Jul 1971, J. A. Slater & R. M. Baranowski & J. E. Harrington, 1 ♂ (00165830) (AMNH). **MEXICO:** Origin unknown, 03 Nov 1988, intercepted at Elpaso, Texas, by APHIS/PPQ, on *Musa* sp. (Musaceae), 1 ♀ (00161863) (USNM). **PANAMA:** ***Pinogana*:** El Real, 08 Aug 1952, F. S. Blanton, 4 ♀♀ (00161850 - 00161853), 1 ♀ (00161849) (USNM). **PERU:** ***Huanuco*:** ***Leoncio Prado Co*.:** Tingo Maria, 671 m, 19 Apr 1969–24 Apr 1969, P. & P. Spangler, 1 ♂ (00161938), 1 ♀ (00161939) (USNM).

### Tytthus fuscicornis

Henry sp. n.

urn:lsid:zoobank.org:act:208FF6B9-938A-4489-AE46-5E4AE9FD5624

http://species-id.net/wiki/Tytthus_fuscicornis

Figs 15, 16 132–135

#### Diagnosis.

This new species is distinguished by the small size, the uniformly dark brown head and pronotum, fuscous to black antennae with segment II thickened and subequal to apical diameter of segment I, the pale or whitish hemelytra, and the brownish-yellow legs. The only known male (holotype) of this species is macropterous and the only known female is brachypterous with an abbreviated membrane.

It is superficically similar to several species, such as *Tytthus mexicanus* and *Tytthus panamensis*, based on the dark head, pronotum and scutellum and pale hemelytra. It is distinguished from these and all other species by the pale tibiae lacking knee spots, uniformly fuscous antennae, the thickened antennal segment II, pale femora, and relatively small size.

#### Description.

*Holotype male* (Fig. 15): Length to apex of hemelytron 2.14 mm, length to base of cuneus 1.54 mm, width across hemelytra 0.69 mm. *Head*: Length 0.29 mm, width across eyes 0.51 mm, interocular width 0.29 mm. *Labium*: Length 0.69 mm. *Antenna*: Segment I length 0.22 mm, II 0.75 mm, III 0.43 mm, IV 0.29 mm. *Pronotum*: Length 0.27 mm, basal width 0.67 mm.

*Coloration*: *Head*: Uniformly dark brown, with a somewhat indistinct, small, yellow interocular spot near inner margin of eye; eyes dark brown to reddish brown, especially around margins. *Labium*: Uniformly brownish yellow, with only apex of segment IV dark brown or fuscous. *Antenna*: Uniformly fuscous to black. *Pronotum*: Uniformly dark brown. *Mesoscutum*: Dark brown. *Scutellum*: Dark brown. *Hemelytron*: Pale or whitish, evenly tinged with pale brown; membrane pale translucent brown. *Ostiolar evaporative area*: Pale brownish yellow. *Ventral surface*: Propleura dark brown; ventral areas of thorax and abdomen reddish brown, genital capsule darker brown to nearly black. *Legs*: Coxae pale to pale brownish yellow, with bases reddish brown; femora brownish yellow, tinged with orange or brownish orange; tibiae, tarsi, and claws pale browish yellow.

*Structure, texture, and vestiture*: *Head*: Shiny, impunctate; buccula slender, ending posteriorly about level with middle of eye; sparsely set with very short, recumbent and semierect, setae. *Labium*: Extending to apices of meso- or bases of metacoxae; segment I short, extending only slightly beyond base of head. *Antenna*: Segment I with only a few short, recumbent setae and two erect, subapical, bristlelike setae; segment I densely set with short, recumbent setae; segment II gradually thickened to apex, apical width equal to or greater than diameter of segment I. *Pronotum*: Shiny, impunctate; calli weakly swollen; anterior angles rounded; lateral margins nearly straight, gradually widening to posterior angles; basal margin weakly concave; sparsely set with only short, recumbent setae. *Mesoscutum*: Narrowly exposed; sparsely set with short, recumbent setae. *Scutellum*: Impunctate, weakly shiny; sparsely set with short, recumbent setae. *Hemelytron*: Entire, suparallel; length of cuneus about two times basal width; membrane with two areoles, extending well beyond apex of abdomen.

**Figures 132–139.**
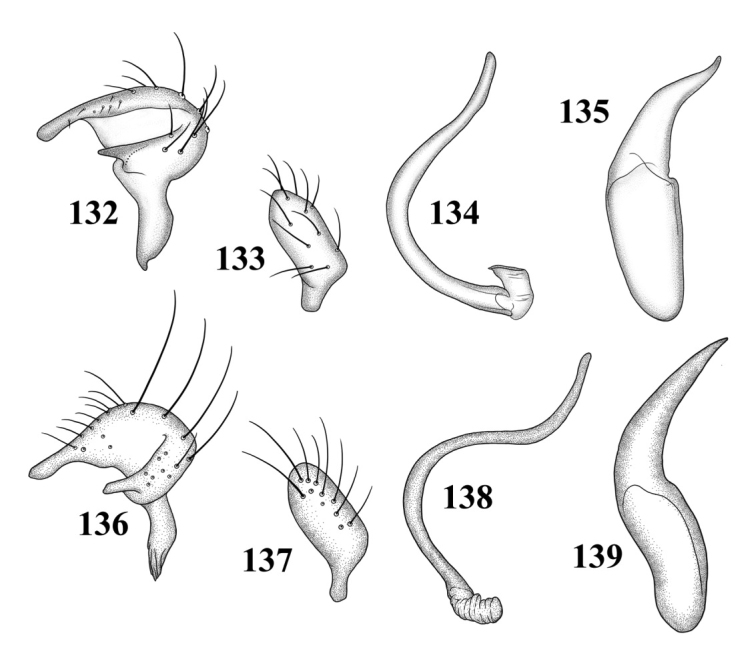
Male genitalia **132–135** *Tytthus fuscicornis* **132** left paramere **133** right paramere **134** endosoma **135** phallotheca **136–139** T. *insperatus* **136** left paramere **137** right paramere **138** endosoma **139** phallotheca.

*Male genitalia*: *Left paramere* (Fig. 132): Mitt-shaped; right arm long, evenly slender, and rounded apically; left arm short, apically acute. *Right paramere* (Fig. 133): elongate oval. *Endosoma* (Fig. 134): C-shaped or weakly S-shaped. *Phallotheca* (Fig. 135): Relatively slender, apically acute.

*Brachypterous female* (n = 1; somewhat teneral) (Fig. 16): Length to apex of hemelytron 1.57 mm, length to base of cuneus 1.36 mm, width across hemelytra 0.70 mm. *Head*: Length 0.27 mm, width across eyes 0.53 mm, interocular width 0.32 mm. *Labium*: Length 0.64 mm. *Antenna*: Segment I length 0.19 mm, II 0.61 mm, III 0.40 mm, IV 0.27 mm. *Pronotum*: Length 0.27 mm, basal width 0.67 mm.

Similar to male in general coloration, but differing in the broader, more oval form, the slightly more slender antennal segment II (but with apex still nearly equal to apical diameter of segment I), and abbreviated hemelytron, with the length of the cuneus subequal to the basal width and the membrane shortened and extending only to the apex of the abdomen.

**Host.** Beaten by A. G. Wheeler from crowns of *Muhlenbergia rigens* (Benth) Hitchc. [Poaceae].

#### Distribution.

Known only from New Mexico, USA.

#### Type material.

**Holotype** ♂ (00161896) (USNM): **UNITED STATES:** **New Mexico:** ***Grant Co*.:** Little Walnut Road, Gila National Forest, North of Silver City, 12 May 2008, A. G. Wheeler, Jr. **Paratype:** Same data as for holotype, 1 ♀ (00161895) (USNM).

### Tytthus insperatus

(Knight)

http://species-id.net/wiki/Tytthus_insperatus

Figs 17, 18 136–139

Cyrtorhinus insperatus Knight 1925: 42 (orig. descrip.).
Tytthus insperatus : Carvalho and Southwood 1955: 31 (descrip., n. comb.); Carvalho 1958: 157 (cat.); Henry and Wheeler 1988: 458 (cat.); Schuh 1995: 248 (cat.).

#### Diagnosis.

This species is distinguished by the black head and antennae, strongly infuscated or brown pronotum with the anterior collarlike margin narrowly whitish, the translucent smoky-brown hemelytra, and the orange-brown legs with a slender, dorsal and anterior red line on each femur, and a posterior red line on the metafemur. All known specimens of this species are fully macropterous.

*Tytthus insperatus* is similar to *Tytthus balli* and *Tytthus uniformis* in overall size, body shape, and general coloration. It differs from both species in having an imbrowned pronotum with a pale or white collar and distinct red lines on the femora.

#### Description.

*Holotype male* (Fig. 17): Length to apex of hemelytron 2.98 mm, length to base of cuneus 2.06 mm, width across hemelytra 0.77 mm. *Head*: Length 0.45 mm, width across eyes 0.59 mm, interocular width 0.27 mm. *Labium*: Length 1.09 mm. *Antenna*: Segment I length 0.35 mm, II 1.44 mm, III 0.91 mm, IV 0.54 mm. *Pronotum*: Length 0.34 mm, basal width 0.70 mm.

*Coloration*: *Head*: Uniformly fuscous to nearly black, with only a small, indistinct pale, interocular spot near inner margin of each eye; eyes fuscous. *Labium*: Pale brownish yellow, apical half of segment IV darker brown. *Antenna*: Uniformly black. *Pronotum*: Dark brown, fading to paler orange brown posteriorly, narrow anterior collar white. *Mesoscutum*: Pale brownish orange. *Scutellum*: Pale brownish orange. *Hemelytron*: Uniformly pale translucent brown, with pale orange highlights; membrane clear or translucent. *Ostiolar evaporative area*: Pale brownish orange. *Ventral surface*: Thoracic area pale brownish orange; abdomen pale with green and orange highlights; genital capsule fuscous. *Legs*: Coxae pale brownish orange; femora pale brownish orange with a slender dorsal and anterior red stripe on pro- and mesofemora and a dorsal, anterior, and posterior red stripe on metafemur; tibiae, tarsi, and claws fuscous to black.

*Structure, texture, and vestiture*: *Head*: Shiny, impunctate, width subequal to length; nearly glabrous with only a few scattered erect and semierect setae; buccula narrow, tapering posteriorly ending near level with middle of eye. *Labium*: Extending to mesocoxae; segment I not extending beyond base of head. *Antenna*: Segment I only slightly thicker than segment II, sparsely set with short, recumbent setae and two erect, subapical, bristlelike setae; segment II densely set with short, recumbent setae. *Pronotum*: Shiny, impunctate; calli weakly swollen; anterior angles angulate; lateral margins convex, flaring at posterior angles; posterior margin weakly concave; nearly glabrous, with only a few scattered, recumbent setae. *Mesoscutum*: Shiny, impunctate, with a few scattered, recumbent setae. *Scutellum*: Shiny, impunctate, with a few scattered, recumbent setae. *Hemelytron*: Macropterous, subparallel, cuneus and membrane fully developed, extending well beyond apex of abdomen; evenly set with short, recumbent setae.

*Male genitalia*: *Left paramere* (Fig. 136): Mitt-shaped; right arm, long, stout, bluntly rounded apically; left arm short, apically blunt. *Right paramere* (Fig. 137): Elongate oval. *Endosoma* (Fig. 138): Strongly S-shaped. *Phallotheca* (Fig. 139): Slender, apically acute.

*Female* (n = 3) (Fig. 18): Length to apex of hemelytron 3.14–3.33 mm, length to base of cuneus 2.33–2.43 mm, width across hemelytra 0.96–1.06 mm. *Head*: Length 0.40–0.43 mm, width across eyes 0.61–0.66 mm, interocular width 0.32–0.34 mm. *Labium*: Length 1.12–1.15 mm. *Antenna*: Segment I length 0.32–0.34 mm, II 1.12–1.47 mm, III 0.96–1.01 mm, IV 0.53–0.54 mm. *Pronotum*: Length 0.34–0.37 mm, basal width 0.77–0.82 mm.

Similar to male in overall coloration and body form.

#### Host.

Unknown. The Maricopa specimen below from “on cotton” almost certainly represents an incidental or sitting record.

#### Distribution.

Described from Tucson (Pima County), Arizona, and later reported from Buckeye (Maricopa County), Arizona, and Calexico (Imperial County), California (Carvalho and Southwood 1955).

#### Type material examined.

**Holotype** ♂ (00162203) (USNM): **UNITED STATES: Arizona: *Pima Co*.:** Tucson, 32.22167°N, 110.92583°W, 07 Jun 1924, A. A. Nichol,. **Paratypes:** Same data as for holotype, 1 ♀ (allotype) (00161894) (USNM), 1 ♀ (00167071) (CNC).

#### Other specimens examined.

**UNITED STATES:** **Arizona: *Cochise Co*.:** W Hereford, Rio San Pedro, 4150’, 22 July 1974, E. R. Hoebeke, 1 ♂ (CU). ***Maricopa Co*.:** Buckeye, 33.37028°N, 112.58306°W, 06 Jun 1935, H. G. Johnston, on cotton, 1 ♂ (00138719) (USNM).

### Tytthus juturnaiba

Carvalho & Wallerstein

http://species-id.net/wiki/Tytthus_juturnaiba

Figs 140–143

Tytthus juturnaiba Carvalho and Wallerstein 1978: 256 (orig. descrip.); Schuh 1995: 249 (cat.).

#### Diagnosis.

Based on the original description and adult habitus illustration, this species can be distinguished by the overall dark color with the cuneus, embolium, and the narrow outer edge of the corium pale yellow, the medially fuscous membrane, the dark antennal segment I with only the apex pale, and the pale antennal segment II with the apex fuscous. This combination of characters places *Tytthus juturnaiba* near the North American *Tytthus vagus*, but differs in having an apparent pale yellow antennal segment II, with only the apex black, and uniformly pale yellow legs, whereas *Tytthus vagus* has a uniformly black antennal segment II, and the hind femur is fuscous on the apical two thirds.

I have, however, studied a photograph of the holotype of *Tytthus juturnaiba* stored in the PBI Heteroptera Species Database (http://research.amnh.org/pbi/heteropteraspeciespage/popupimage.php?imagename=AMNH_PBI 00174988.jpg) that appears to indicate that antennal segment I actually is pale at the base and apex (not just pale at the apex) and segment II is uniformly dark (not yellow, with the apex fuscous). Based on this photograph, which contradicts the antennal characters described in the original description, *Tytthus juturnaiba* almost certainly is conspecific with *Tytthus neotropicalis* and would run to this species in my key. Nevertheless, I refrain from making a formal synonymy until I can examine the holotype (which was unvailable at the time of this study) deposited in the Museu Nacional, Rio de Janeiro, Brazil.

#### Description.

*Holotype male* (based Carvalho and Wallerstein 1978): Length to apex of hemelytron 2.90 mm, width across hemelytra 0.90 mm. *Head*: Length 0.20 mm, width across eyes 0.60 mm, interocular width 0.28 mm. *Antenna*: Segment I length 0.30 mm, II 0.80 mm, III 0.50 mm, IV missing. *Pronotum*: Length 0.40 mm, basal width 0.80 mm. *Cuneus*: Length 0.40 mm, basal width 0.28 mm.

*Coloration* (based on Carvalho and Wallerstein 1978): General coloration black, outer margin of exocorium, embolium, and cuneus pale yellow; middle of membrane fuscous; antenna pale yellow, segment I (except extreme apex) and apex of segment II black. Undersurface black. Legs pale yellow.

*Structure, texture, and vestiture* (based on Carvalho and Wallerstein 1978): Information not given.

**Figures 140–147.**
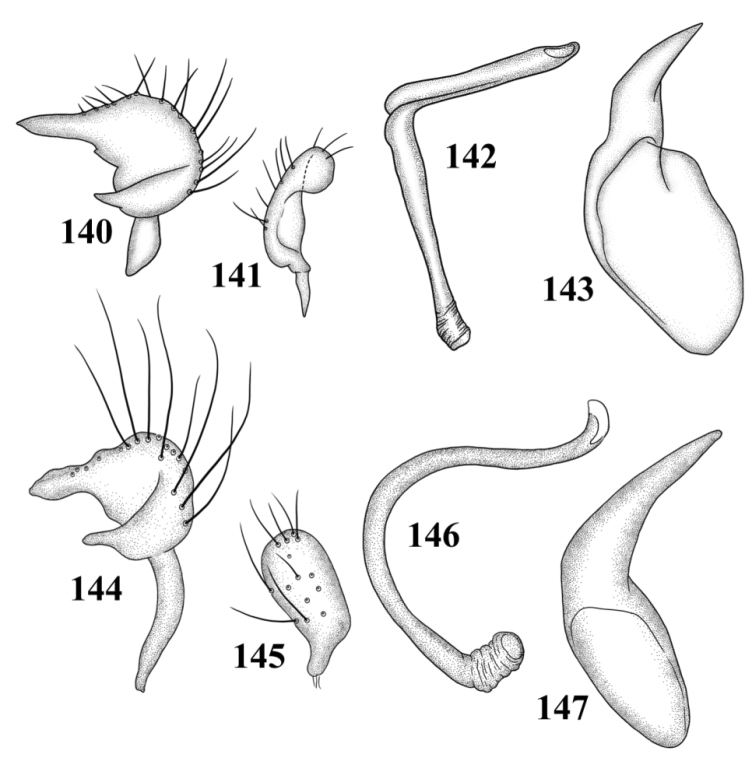
Male genitalia **140–143** *Tytthus juturnaiba* **140** left paramere **141** right paramere **142** endosoma **143** phallotheca **144–147** *Tytthus mexicanus* **144** left paramere **145** right paramere **146** endosoma **147** phallotheca.

*Male genitalia* (based on Carvalho and Wallerstein 1978): *Left paramere* (Fig. 140): Mitt-shaped; right arm longest; left arm shorter, spinelike. *Right paramere* (Fig. 141): Elongate oval. *Endosoma* (Fig. 142): Strongly C-shaped, bent at middle. *Phallotheca* (Fig. 143): Broad, apically acute.

*Female* (based on Carvalho and Wallerstein 1978): Length to apex of hemelytron 3.20 mm, width across hemelytra 1.00 mm. *Head*: Length 0.20 mm, width across eyes 0.50 mm, interocular width 0.28 mm. *Antenna*: Segment I length 0.20 mm, II 0.80 mm, III and IV missing. *Pronotum*: Length 0.40 mm, basal width 0.90 mm. *Cuneus*: Length 0.44 mm, basal width 0.28 mm.

#### Host.

Unknown.

#### Distribution.

Described and known only from the holotype and allotype taken at Logoa Juturnaiba, Araruama, Estado do Rio de Janeiro, Brazil, Nov. 1976 (Carvalho and Wallerstein 1978).

#### Specimens examined.

None.

### Tytthus mexicanus

Henry sp. n.

urn:lsid:zoobank.org:act:12D9E8C9-F915-463E-A1F4-22F8D2E97CB0

http://species-id.net/wiki/Tytthus_mexicanus

Figs 19, 20 144–147

#### Diagnosis.

This species is distinguished by the black antennae, except for the pale apex of segment I; the black pronotum; the brown mesoscutum and scutellum; the smoky brown to dirty white hemelytra; and the pale yellowish legs, with distinct black spines and knee spots at the bases of the tibiae.

*Tytthus mexicanus* is most similar to *Tytthus femoralis* and *Tytthus entrerianus* based on the pale tibiae with knee spots and antennal segment II lacking erect, bristlelike setae. It can be distinguished from both species by the uniformly pale hind femora and dark brown to fuscous antennal segment II.

#### Description.

*Male* (n = 4; plus holotype in parentheses) (Fig. 19): Length to apex of hemelytron 2.75–2.58 mm (2.55 mm), length to base of cuneus 2.90–2.05 mm, width across hemelytra 0.88-0.93 mm (0.86 mm). *Head*: Length 0.32–0.34 mm (0.30 mm), width across eyes 0.59–0.61 mm (0.59 mm), interocular width 00.27–0.29 mm (0.27 mm). *Labium*: Length 1.12–1.14 mm (1.12 mm). *Antenna*: Segment I length 0.30–0.34 mm (0.32 mm), II 0.91–1.07 (1.02 mm), III 0.54–0.64 mm (0.58 mm), IV 0.51–0.64 mm (missing). *Pronotum*: Length 0.34–0.35 mm (0.32 mm), basal width 0.78–0.82 mm (0.77 mm).

*Coloration*: *Head*: Uniformly fuscous to black, with a large, distinct, pale yellow, interocular spot near inner margin of each eye, spots nearly contiguous in some specimens; eyes reddish brown, often fading to silver with a reddish tinge. *Labium*: Uniformly pale yellow; apex of segment IV dark brown to fuscous. *Antenna*: Segment I fuscous to black, with apical one fourth yellow (length of yellow area equal to diameter of segment) and narrowed basal one fourth shiny; segment II dark brown to black (basal area darker on “paler” dark brown segments); segments III and IV dark brown to fuscous. *Pronotum*: Uniformly shiny fuscous, weakly swollen calli sometimes very slightly paler brown. *Mesoscutum*: Dark brown to fuscous, lateral angles pale yellow on some specimens. *Scutellum*: Dark brown to fuscous, slightly paler apically. *Hemelytron*: Pale translucent smoky brown to dirty white; veins brown and often narrow inner margin of clavus brown. *Ostiolar evaporative area*: Dark brown to fuscous, often invaded with pale areas posteriorly. *Ventral surface*: Thorax uniformly dark brown to fuscous; abdomen dark brown to fuscous laterally, pale ventrally, genital capsule black. *Legs*: Coxae pale brownish yellow, with bases brown; femora uniformly pale yellow to whitish; tibia pale yellow to whitish with spines and bases, or knees, dark brown to fuscous; tarsi and claws pale yellow.

*Structure, texture, and vestiture*: *Head*: Shiny, impunctate, wider than long; buccula relatively narrow, ending posteriorly near level with middle of eye; sparsely set with long, erect and suberect, pale brown to brown setae. *Labium*: Extending to bases of hind coxae or just onto abdominal segment II; segment I extending to bases of fore coxae. *Antenna*: Segment I with short, recument setae and two long, black, bristlelike setae near apex before pale area; segment II thickly set with short, recumbent pale brown setae much shorter than diameter of segment. *Pronotum*: Shiny, impunctate; calli weakly swollen but distinct; anterior rounded; lateral margins concave, strongly flaring at posterior angles; posterior margin weakly sinuate; set with short, erect to semierect setae. *Mesoscutum*: Shiny, impunctate, broadly exposed; with numerous semierect and recumbent, pale brown setae. *Scutellum*: Shiny, impunctate; thickly set with semierect and recumbent pale brown setae. *Hemelytron*: Macropterous, subparallel when paired, with fully developed cuneus and membrane, extending well beyond apex of abdomen; evenly scattered with short, recumbent, pale brown setae.

*Male genitalia*: (Fig. 144): Mitt-shaped; right arm longest, stout, slightly constricted basally, weakly pointed apically; left arm short, apically acute. *Right paramere* (Fig. 145): Elongate oval, strongly rounded apically. *Endosoma* (Fig. 146): C-shaped, nearly S-shaped with apical quarter curving slightly upward. *Phallotheca* (Fig. 147): moderately stout, apically acute.

*Female*:(n = 2) (Fig. 20): Length to apex of hemelytron 2.88–2.93 mm, length to base of cuneus 2.10–2.23 mm, width across hemelytra 0.99–1.04 mm. *Head*: Length 0.32–0.37 mm, width across eyes 0.61–0.62 mm, interocular width 0.29–0.30 mm. *Labium*: Length 1.20–1.22 mm. *Antenna*: Segment I length 0.29–0.30 mm, II 0.88–0.91 mm, III 0.61–0.64 mm, IV 0.61–0.62 mm. *Pronotum*: Length 0.35–0.37 mm, basal width 0.86–0.88 mm.

Similar to male in general color and pubescence, differing primarily in the slightly broader form.

#### Etymology.

Named for the country in which it was collected, Mexico.

#### Host.

Unknown.

#### Distributon.

Known only from Baja California Sur, Nayarit, and Sinaloa, Mexico.

#### Type material.

**Holotype** ♂ (00166142) (USNM): **MEXICO:** **Sinaloa:** ***Culiacan Co*.:** Camino Real Tres Rios, 3 km N Mex. 15 at toll bridge gate, 08 Aug 1981, S. Nichols. **Paratypes**: **MEXICO:** **Nayarit:** San Blas, 16 Oct 1973, S.C. Williams, K.B. Blair, & C.L. Mullinex, 1 ♂ (00409874), 1♀ (00409875) (CAS). **Sinaloa:** ***Culiacan Co*.:** Camino Real Tres Rios, 3 km N Mex. 15 at toll bridge gate, 08 Aug 1981, S. Nichols, 2 ♂ (00163423, 00166141) (USNM). **Other Paratypes**: **MEXICO:** **Baja Calif. Sur:** San Javier 11 Oct 1981, D. Faulkner & F. Andrews, at blacklight, 1 ♂ (CDFA). 112.2 mi SE San Perdito near Rancho Saucito, 8 Oct 1981, F. Andrews & D. Faulkner, 1 ♀ (SDNH). ***Nayarit***: Choix, 5 Aug 1968, A. Sears, R. C. Gardner, & C. S. Glaser, 1 ♂ (UCD). 5.5 mi NW Choix, 14 Jul 1968, A. Sears, R. C. Gardner, & C. S. Glaser, 1 ♀ (UCD). Mazatlan, 27 Mar 1979, L. D. French, 1 ♂ (UCD).

### Tytthus montanus

Carvalho & Southwood

http://species-id.net/wiki/Tytthus_montanus

Figs 21–23 148–151

Tytthus montanus Carvalho and Southwood 1955: 32 (orig. descrip.); Carvalho 1958: 157 (cat.); Henry and Wheeler 1988: 458 (cat.); Schuh 1995: 249 (cat.).

#### Diagnosis.

This species is distinguished by the overall dark brown to nearly black coloration, with the basal thirds of the corium and clavus white, the pale yellow antennal segment I and black segment II, the dark brown metafemur, and pale yellow tibiae. It is known from only two macropterous males (Fig. 21), one macropterous female (Fig. 22), and numerous brachypterous females (Fig. 23).

*Tytthus montanus* is similar to *Tytthus wheeleri* in having mostly dark brown hemelytra with only the base pale or white but is distinguished from that species by the larger size (2.70 mm vs. less than 2.00 mm in *Tytthus wheeleri*), pale yellow antennal segment I, red-streaked pro- and mesofemora, and dark brown metafemur (versus entirely pale yellow legs in *Tytthus wheeleri*). It is also similar to *Tytthus alboornatus* but is distinguished by the distally dark hemelytron (versus distally white or pale cuneus or pale area across posterior margin of hemelytra in brachypters).

#### Description.

*Macropterous male* (n=1; holotype in parentheses) (Fig. 21): Length to apex of hemelytron 2.83 mm (2.75 mm), length to base of cuneus 1.98 mm (1.75 mm), width across hemelytra mm. *Head*: Length 0.38 mm (0.37 mm), width across eyes 0.64 mm (0.64 mm), interocular width 0.34 mm (0.32 mm). *Labium*: Length 0.98 mm (0.93 mm). *Antenna*: Segment I length 0.27 mm (0.30 mm), II 0.93 mm (0.93 mm), III 0.56 mm (missing), IV mm (missing). *Pronotum*: Length 0.45 mm (0.43 mm), basal width 0.72 mm (0.74 mm).

*Coloration*: *Head*: Dark reddish brown (holotype) to black; pale yellow interocular spot near inner margin of eye indistinct; eyes dark brown to nearly black. *Labium*: Mostly pale yellow, with segment I and apex of segment IV dark brown. *Antenna*: Segment I pale yellow, narrowly fuscous at base; segments II mostly fuscous to black, with only base pale yellow; segments III–IV nearly black (segments II–IV yellowish brown in holotype). *Pronotum*: Uniformly dark brown (holotype) to black. *Mesoscutum*: Uniformly dark brown (holotype) to black. *Scutellum*: Uniformly dark brown (holotype) to black. *Hemelytron*: Largely dark brown (holotype) to black, with only basal third of corium and clavus white; membrane translucent brown to smoky black. *Ostiolar evaporative area*: Dark reddish brown (holotype) to black. *Ventral surface*: Uniformly dark reddish brown (holotype) to black. *Legs*: Coxa pale yellow, reddish brown or black at bases; pro- and mesofemora pale yellow, lightly tinged with pale orange to more reddish brown with bases pale (holotype), metafemur dark reddish brown (holotype) to dark brown, with basal one third and apex pale yellow; pro- and mesotibiae pale yellow to more reddish brown with bases pale, metatibia yellow (holotype) to dark brown; tarsi and claws pale yellow.

*Structure, texture, and vestiture*: *Head*: Shiny, impunctate, width subequal to length; buccula very narrow, tapering posteriorly, ending at level before middle of eye. *Labium*: Extending to bases of mesocoxae; segment I not extending to base of head. *Antenna*: Segment I with only very short, fine, recumbent setae, with two erect, subapical, bristlelike setae; segment II thickly set with short, recumbent setae, intermixed with a few more erect, short setae on distal half. *Pronotum*: Shiny, with a glaucous sheen over weakly defined calli, impunctate; anterior angles rounded, lateral margins concave, strongly flaring at posterior angles; posterior margin weakly sinuate; sparsely set with short, fine, recumbent setae. *Mesoscutum*: Shiny, impunctate, with a few scattered, recumbent setae. *Scutellum*: Shiny, impunctate, with a few short, fine, recumbent setae. *Hemelytron*: Macropterous, cuneus and membrane fully developed, extending well beyond apex of abdomen; evenly set with scattered, short, fine, recumbent setae.

**Figures 148–155.**
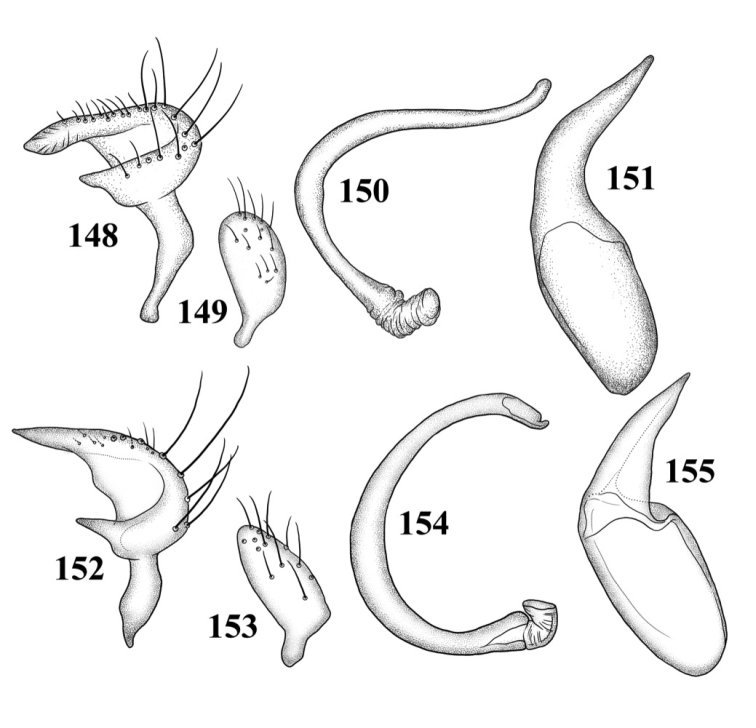
Male genitalia **148–151** *Tytthus montanus* **148** left paramere **149** right paramere **150** endosoma **151** phallotheca **152–155** *Tytthus mundulus* **152** left paramere **153** right paramere **154** endosoma **155** phallotheca.

*Male genitalia*: *Left paramere* (Fig. 148): Mitt-shaped; right arm longest, stout, distally blunt; left arm short, blunt. *Right paramere* (Fig. 149): Oval. *Endosoma* (Fig. 150): C-shaped to weakly S-shaped. *Phallotheca* (Fig. 151): Slender, apically acute.

*Macropterous female* (n = 1) (Fig. 22): Length to apex of hemelytron 2.65 mm, length to base of cuneus 1.87 mm, width across hemelytra 0.94 mm. *Head*: Length 0.37 mm, width across eyes mm, interocular width 0.32 mm. *Labium*: Length 0.91 mm. *Antenna*: Segment I length 0.22 mm, II 0.59 mm, III 0.40 mm, IV 0.40 mm. *Pronotum*: Length 0.42 mm, basal width 0.72 mm.

Very similar to the two macropterous males in overall shape and structure, differing in the significantly shorter antennal segments and in having all femora and the basal halves of the tibiae dark brown to fuscous.

*Brachypterous female* (n = 5) (Fig. 23): Length to apex of hemelytron 1.85–2.20 mm, length to base of cuneus 1.53–1.75 mm, width across hemelytra 0.77-0.86 mm. *Head*: Length 0.38–0.42 mm, width across eyes 0.61–0.62 mm, interocular width 0.32–0.34 mm. *Labium*: Length 0.88-0.91 mm. *Antenna*: Segment I length 0.22-0.24 mm, II 0.64–0.78 mm, III 0.35–0.42 mm, IV 0.34–0.35 mm. *Pronotum*: Length 0.37–0.40 mm, basal width 0.56–0.58 mm. Similar to macropters in overall color pattern, but like the macropterous female, differing from males in the shorter antennal segment and darkened femora.

Similar in color to the macropterous males and the one macropterous female, but differing especially in the nearly quadrate pronotum that has only weakly flared posterior margins and the brachypterous hemelytron with a greatly shortened cuneus (fracture still visible on most specimens) and a much abbreviated membrane lacking any trace of areoles (Fig. 23).

#### Host.

No specific host. Specimens from Arizona were swept from a dry grass and flower meadow in eastern Arizona; the one male from Utah was beaten from the bases of bunch grasses.

#### Distribution.

Described and previously known only from Montana. Arizona and Utah represent new state records.

#### Discussion.

Although I have considered the series of 18 females collected in Arizona by Leonard Kelton (CNC) conspecific with the holotype male and one other male taken in northern Utah, I note that all femora of this southern population are uniformly dark except at the bases, whereas the Montana and Utah males have pale front and middle femora and only the hind femora dark. In addition, the actual measurements of the antennal segments are considerably smaller in these females than males, a variation not observed in other species.

#### Type material examined.

**Holotype** ♂ (macropterous) (00162198) (USNM): **UNITED STATES:** **Montana:** ***Granite Co*.:** Drummond, July 10, 1935, Oman.

#### Other specimens examined.

**UNITED STATES:** **Arizona:** ***Apache Co*.:** Big Lake, Apache National Forest, 33.88667°N, 109.41667°W, 2774 m, 12 Aug 1967 - 14 Aug 1967, L. A. Kelton, 18 ♀♀ (00112047 - 00112061, 00112063, 00161925 - 00161926) (CNC). **Utah:** ***Cache Co*.:** 0.4 mi off Rt 89 along Franklin Basin Rd, 41.93555°N, 111.57222°W, 2004 m, 17 Jul 2001 - 18 Jul 2001, T. J. Henry and A. G. Wheeler, Jr., 1 ♂ (00161897) (USNM).

### Tytthus mundulus

(Breddin)

http://species-id.net/wiki/Tytthus_mundulus

Figs 24, 25 152–155

Periscopus mundulus Breddin 1896: 106 (orig. descrip).
Cyrtorhinus mundulus : Reuter 1902: 178 (descrip.); Kirkaldy 1908: 378 (list); Poppius 1914: 168 (list); Muir 1920: 125 (notes); Williams 1931: 103 (notes); Swezey 1936: 79 (notes, history); Usinger 1939: 271 (key, host); Zimmerman 1948: 206 (note); Usinger 1951: 4 (descrip.).
Tytthus mundulus : Carvalho and Southwood 1955: 26 (key, descrip.); Carvalho 1958: 158 (cat.); Nguyen et al.1984: 265 (note); Henry and Wheeler 1988: 458 (cat.); Schuh 1995: 249 (cat.); Cassis and Gross 1995: 204 (cat., hosts); Kerzhner and Josifov 1999: 441 (cat.); Wheeler 2000: 665 (hosts, biol., distr.), 2001: 278 (hosts, biol., distr.); Yasunaga et al. 2001: 182 (note, photo).

#### Diagnosis.

*Tytthus mundulus* is distinguished by the fuscous to black head, pronotum, and scutellum; the translucent hemelytra with the clavus, most of the corium, and membrane tinged with brown; and the yellow legs and antennal segment I (apex sometimes narrowly infuscated), with contrasting fuscous to black antennal segments II–IV. Antennal segment II has short erect setae along the entire dorsal and ventral surface (most evident ventrally), similar to those found on in *Tytthus chinensis* and *Tytthus parviceps*; *Tytthus mundulus*, however, lacks the fuscous knee spots found in these two species. All known specimens of this species are macropterous.

#### Description.

*Male* (n =5) (Fig. 24): Length to apex of hemelytron 3.07–3.26 mm, length to base of cuneus 2.21–2.30 mm, width across hemelytra 0.93–1.01 mm. *Head*: Length 0.34–0.35 mm, width across eyes 0.70–0.72 mm, interocular width 0.34–0.35 mm. *Labium*: Length 1.04–1.15 mm. *Antenna*: Segment I length 0.38–0.40 mm, II 1.23–1.26 mm, III 0.66–0.67 mm, IV 0.54–0.56 mm. *Pronotum*: Length 0.38–0.40 mm, basal width 0.83–0.86 mm.

*Coloration*: *Head*: Uniformly black, with an indistinct, pale yellow, interocular spot near inner margin of each eye; eyes dark reddish brown. *Labium*: Pale yellow, with only apical half of segment IV brown. *Antenna*: Segment I pale yellow, sometimes tinged with pale brownish orange, apex sometimes narrowly dark brown; segments II–IV dark brown to fuscous. *Pronotum*: Uniformly shiny, black. *Mesoscutum*: Shiny black. *Scutellum*: Shiny black. *Hemelytron*: Clavus, inner two thirds of corium and most of membrane tinged with brown to dark brown, leaving only outer margin of corium clear. *Ostiolar evaporative area*: Dark brown to fuscous. *Ventral surface*: Uniformly dark brown to fuscous. *Legs*: Coxae yellow, with bases dark brown to fuscous; femora, tibiae, tarsi, and claws uniformly yellow.

*Structure, texture, and vestiture*: *Head*: Shiny, impunctate, with a glaucous patch along inner margin of each eye; wider than long; buccula relatively broad, ending near level with posterior margin of eye; sparsely set with short, recumbent setae, more so on glaucous patches, and with a few longer, erect setae along posterior margin. *Labium*: Extending to bases of mesocoxae; segment I extending beyond base of head to anterior edge of prosternum. *Antennae*: Segment I sparsely set with short, fine, recumbent setae and two erect, subapical, bristlelike setae; segment II thickly set dorsally and ventrally with short, erect and semierect, somewhat bristlelike setae, forming a “bottlebrush” appearance (similar to *Tytthus chinensis*). *Pronotum*: Shiny, impunctate; calli weakly swollen; anterior angles rounded; lateral margins weakly concave, moderately flaring at posterior angles; posterior margin sinuate; thickly set with semierect and recumbent setae. *Mesoscutum*: Shiny, impunctate, broadly exposed; with semierect and recumbent setae. *Scutellum*: Shiny, impunctate, equilateral; thickly set with semierect and recumbent setae. *Hemelytron*: Macropterous, subparallel when paired, with fully developed cuneus and membrane, extending well beyond apex of abdomen.

*Male genitalia*: *Left paramere* (Fig. 152): Mitt-shaped; right arm longest, gradually tapering to a point; left arm short, apically acute. *Right paramere* (Fig. 153): Elongate oval. *Endosoma* (Fig. 154): Strongly C-shaped. *Phallotheca* (Fig. 155): Relatively slender, apically acute.

*Female* (n = 5) (Fig. 25): Length to apex of hemelytron 3.07–3.64 mm, length to base of cuneus 2.27–2.66 mm, width across hemelytra 1.04–1.22 mm. *Head*: Length 0.35–0.37 mm, width across eyes 0.74–0.77 mm, interocular width 0.35–0.37 mm. *Labium*: Length 1.06–1.22 mm. *Antenna*: Segment I length 0.34–0.38 mm, II 0.98–1.10 mm, III 0.58–0.59 mm, IV 0.50–0.58 mm. *Pronotum*: Length 0.40–0.43 mm, basal width 0.91–1.01 mm.

#### Hosts.

This species is an egg predator of the sugarcane delphacid, *Perkinsiella sacharicida* Kirkaldy (Zimmerman1948, Wheeler 2001). It has also been taken on corn and taro in Hawaii.

#### Distribution.

This Indo-Pacific species has been reported from Fiji, Hawaii, New Caledonia, Papua New Guinea, the Philippine Islands, and Queensland, Australia (Schuh 1984). It has been successfully introduced into Hawaii to control the sugarcane delphacid (Zimmerman 1948, Carvalho and Southwood 1955), representing one of the best examples of successful classical biological control (Wheeler 2001). It was released in Florida, according to Nguyen et al. (1984), but apparently it has not become established (Wheeler 2001). It was also introduced into South Africa to control a tropiduchid, *Numicia viridis* Muir, on sugarcane but without success (Carnegie and Harris 1969). See Wheeler (2001) for additional information about this beneficial species.

#### Specimens examined.

**AUSTRALIA:** **Queensland:** ***North Queensland Co*.:** Halifax Apr. 1920, F. Muir, 1 ♀ (BMNH). Upper Mulgrave River, 8 miles from Goldsborough Road, 09 May 1967, D. H. Colless, 1 nymph (00161946) (USNM). **FIJI:** Natova, Apr. 1919, R. Veitch, 1 ♂, 5♀♀ (BMNH). **MAURITIUS:** Locality unknown, 20.2°S, 57.5°E, 02 May 1965, J.R. Williams, 2 ♂♂ (00138689, 00138743) (AMNH). **PAPUA NEW GUINEA:** **Eastern Highlands:** No. 11, Arau, Kratke Mountains, Valley of the upper Wanton River, 12 Oct 1959, L. J. Brass, 1 ♂ (00138756) (AMNH). **Morobe Province:** Lae at head of Huon Gulf near mouth of Markham River, camp #1, 6.723°S, 146.991°E, 2 m, 07 Oct 1959 - 19 Oct 1959, L. J. Brass, 1 ♀ (00095355) (AMNH). **South High Province**: Upper Mendi, Hoai Village, 1970 m, 4-5 Nov. 1981, B. M. Thistleton, from sugarcane, 2 ♂♂, 1 ♀ (BMNH). **PHILIPPINES:** **Luzon:** Los Banos, 21 Nov 1921, F. X. Williams, sugarcane, 1 ♀ (00161942) (USNM). **UNITED STATES:** **Hawaii:** ***Honolulu Co*.:** Honolulu, Mar 1930, F.C. Hadden, 2 ♂♂ (00167072, 00167073) (CNC). Intercepted on ship at Honolulu port which originated from Treasure Island, California, 08 Mar 1939, R. G. Oakley, 1 ♂ (00162001) (USNM). Waialua, 22 Apr 1925, F. Muir, 1 nymph (00161928) (USNM); 22 Jan 1930, O. H. Swezey, 1 ♀ (00161919) (USNM). Waipio, 02 May 1923, O. H. Swezey, 1♀ (00161918) (USNM). ***Maui Co*.:** 2191 S. Kihei Road, 29 Feb 1984, G. M. Stonedahl, at mercury vapor light, 30 ♂♂ (00138660, 00138662, 00138666, 00138668, 00138672 - 00138673, 00138682, 00138693, 00138711, 00138745, 00138749, 00138754, 00138757, 00138760, 00138787, 00165832 - 00165846), 8 ♀♀ (00165847 - 00165854) (AMNH), 2 ♂♂ (00161944, 00161945) (USNM). ***Oahu Co*.:** Barber’s Point, Mar 1960, E. J. Ford, Jr., at light, 1 ♂ (00161947), 4 ♂ (00161952 - 00161954, 00161958) (USNM). Ewa Beach, 22 Nov 1982, collector unknown, 2 ♂♂ (00161949, 00161957), 2 ♀♀ (00161955, 00161956) (USNM). Honolulu, 21.3069°N, 157.8583°W, 35 m, 24 Feb 1943, N. L. H. Krauss, on corn plants, 1 ♀ (00161940) (USNM). John Rodgers [airport], May 1958, E. J. Ford, Jr., corn, 1 ♂ (00161941) (USNM). Kailua, 09 Oct 1930, O. H. Swezey, 1 ♂ (00161920) (USNM); 15 Sep 1950, T.H., feeding on corn leafhopper eggs, 1 ♂ (00161943) (USNM). Mokuleia, 05 Feb 1940, T. H., on green corn, 2 ♂♂ (00161950, 00161951), 1 ♀ (00161948) (USNM). Punloa, 21 Dec 1926, O. H. Swezey, 1 ♂ (00161922) (USNM). Waianae, 13 Jan 1931, O. H. Swezey, Taro, 1 ♀ (00161921) (USNM).

### Tytthus neotropicalis

(Carvalho)

http://species-id.net/wiki/Tytthus_neotropicalis

Figs 26, 27 63–70 156–159

Cyrtorhinus costae : Carvalho 1945: 316 (misident., habitus, parameres).
Cyrtorhinus neotropicalis Carvalho1954: 425 (orig. descrip.).
Tytthus neotropicalis : Carvalho and Southwood 1955: 25 (n. comb.); Carvalho 1958: 158 (cat.); Carvalho and Rosas 1965: 210 (list); Maldonado 1969: 88 (descrip., Figs); Carvalho and Afonso 1977: 9 (list); Schuh 1995: 249 (cat.); Hernández and Henry 2010: 123 (diag., hosts).

#### Diagnosis.

This species is readily distinguished from all other species of *Tytthus* by the dark brown to fuscous head, pronotum, and scutellum; pale translucent hemelytra; pale yellow legs; and especially the pale first antennal segment having a broad, dark band through the middle. No other species has a broad band on antennal segment I with the base and apex pale. Males and females of this species are always macropterous.

As noted in the diagnosis of *Tytthus juturnaiba*, a photograph of the holotype stored in the PBI Heteroptera Species Database contradicts the color of antennal segments I and II described in the original description. If my interpretation of the banded antennal segment I in the photograph is correct, the two species probably are conspecific. A final decision, however, must await examination of the holotype of *Tytthus juturnaiba*.

#### Description.

*Male* (n = 10) (26, 63, 64): Length to apex of hemelytron 2.40–2.60 mm, length to base of cuneus 1.68–1.80 mm, width across hemelytra 0.82–0.83 mm. *Head*: Length 0.27–0.29 mm, width across eyes 0.54–0.59 mm, interocular width 0.27–0.29 mm. *Labium*: Length 0.94–1.07 mm. *Antenna*: Segment I length 0.24–0.29 mm, II 0.75–0.96 mm, III 0.37–0.51 mm, IV 0.32–0.34 mm. *Pronotum*: Length 0.32–0.34 mm, basal width 0.74–0.77 mm.

*Coloration*: *Head* (Figs 65–67: Uniformly black, with an indistinct, pale yellow, interocular spot near inner margin of each eye; eyes reddish brown. *Labium*: Pale yellow, with apical half of segment IV brown. *Antenna*: Segment I pale or whitish on apical and basal fourth, with a broad, uniformly dark brown to fuscous band through middle and a very narrow dark brown ring at base; segments II–IV uniformly dark brown to fuscous. *Pronotum*: Uniformly dark brown to fuscous. *Mesoscutum*: Uniformly dark brown to fuscous. *Scutellum*: Uniformly dark brown to fuscous. *Hemelytron*: Translucent, highlighted or tinged with pale brown on clavus and inner half of corium, inner half of clavus along claval commissure accented with darker brown; translucent dusky brown. *Ostiolar evaporative area* (Fig. 68): Dark reddish brown. *Ventral surface*: Thorax and abdomen uniformly dark reddish brown to fuscous. *Legs*: Coxae pale yellow, with bases reddish brown; femora pale yellowish, with metafemur sometimes accented with pale orange; tibiae, tarsi, and claws (Fig. 70) pale yellow.

*Structure, texture, and vestiture*: *Head*: Shiny, impunctate, wider than long; buccula narrow, tapering posteriorly, ending near hind margin of eye; set with scattered, relatively long, semierect setae. *Labium*: Extending just beyond metacoxae to base of abdomen; segment I extending past base of head to middle of xyphyus before procoxae. *Antenna*: Segment I with short, relatively sparse, recumbent setae and two or three erect, subapical, bristlelike setae; segment II evenly set with short, recumbent setae. *Pronotum*: Shiny, impunctate; calli weakly swollen, entire area covered with a glaucous sheen; anterior angles rounded; lateral margins concave, strongly flaring at posterior angles; posterior margin weakly sinuate; evenly set with recumbent and semierect setae, especially on disc. *Mesoscutum*: Broadly exposed; set with scattered semierect setae. *Scutellum*: Impunctate, equilateral; set with scattered, relatively long, semierect setae. *Hemelytron*: Macropterous, cuneus and membrane fully developed, extending well beyond apex of abdomen; evenly set with recumbent setae.

**Figures 156–162.**
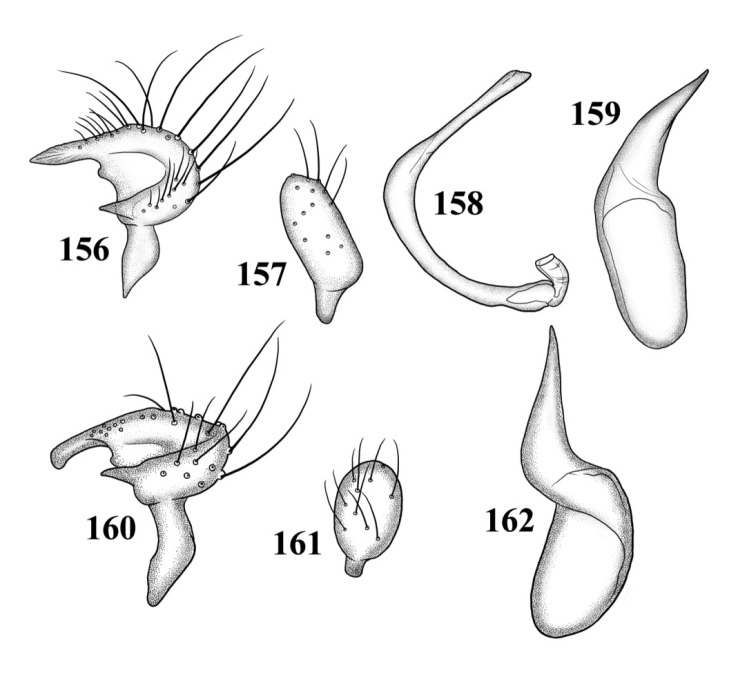
Male genitalia **156–159** *Tytthus neotropicalis* **156** left paramere **157** right paramere **158** endosoma **159** phallotheca **160–163** *Tytthus pallidus* **160** left paramere **161** right paramere **162** phallotheca.

*Male genitalia* (Fig. 69): *Left paramere* (Fig. 156): Mitt-shaped; right arm long and broad, tapering to a point apically; left arm shorter, pointed. *Right paramere* (Fig. 157): Elongate oval. *Endosoma* (Fig. 158): Strongly C-shaped. *Phallotheca* (Fig. 159): Relatively slender, apically acute.

*Female* (n = 10) (Fig. 27): Length to apex of hemelytron 2.66–3.01 mm, length to base of cuneus 2.02–2.18 mm, width across hemelytra 0.96–1.04 mm. *Head*: Length 0.29–0.32 mm, width across eyes 0.56–0.61 mm, interocular width 0.27–0.32 mm. *Labium*: Length 1.06–1.17 mm. *Antenna*: Segment I length 0.26–0.27 mm, II 0.70–0.86 mm, III 0.45–0.54 mm, IV 0.43–0.45 mm. *Pronotum*: Length 0.32–0.35 mm, basal width 0.80–0.86 mm.

Similar to males in overall appearance, differing primarily in the slightly broader body form.

#### Hosts.

No specific host known. Associated with grass savannas and marshes and pine-oak sand scrub.

#### Distribution.

This species was described from Brazil (Carvalho 1954) and later reported from Ecuador, Peru, Puerto Rico, Surinam (Schuh 1995), and Cuba (Hernández and Henry 2010). New country records are Argentina, Belize, Bolivia, Brazil, Guyana, Haiti, Mexico (Veracruz), Nicaragua, Panama, Paraguay, the United States (Florida), and Venezuela.

#### Specimens examined.

**ARGENTINA:** **Misiones:** Parque Nacional de Iguazú, 25.61666°S, 54.33333°W, Dec 1975, collector unknown, 1 ♀ (00095357) (AMNH). **BELIZE:** **Toledo:** Punta Gorda, 16.1°N, 88.8°W, Mar 1931, J.J. White, 1 ♀ (00161324) (USNM). **BOLIVIA:** **El Beni:** 40 km E. San Borja, Estacion Biologica Beni, Estancia El Porvenir, 06 Sep 1987 - 08 Sep 1987, W. E. Steiner, at black light trap in open grass savanna and marsh, 4 ♂♂ (00161989 - 00161990, 00161994, 00161997) (USNM). Capivara on Rio Itenez, approx. 20 km E. of Versalles, 22 Jul 1964, J. K. Bouseman, J. Lussenhop, 1 ♂ (00165932) (AMNH). **Sara:** Locality unknown, 1700, Steinbach, 1 ♂ (00138669) (USNM). **BRAZIL:** **Amazonas:** Estirao do Equador, AM, 4.52378°S, 71.56085°W, Oct 1979, Alvarenga, 1 ♂ (00161385) (USNM). **Ceara:** Barbalha, 7.3167°S, 39.2833°W, May 1969, M. Alvarenga, 1 ♂ (00161384) (USNM). **Minas Gerais:** Vicosa, 20.75°S, 42.8833°W, 653 m, 13 Oct 1985 - 01 Nov 1985, T. J. Henry and P. S. F. Ferreira, 15 ♂♂ (00161893, 00161959 - 00161965, 00161968 - 00161972, 00161978, 00162006), 18 ♀♀ (00161892, 00161966 - 00161967, 00161973 - 00161977, 00161979 - 00161988) (USNM). **Para:** ***Belem Co*.:** 8 km E Belem, Ananindeua, 12 Jun 1973, R. T. Schuh, 1 ♀ (00138665) (USNM). Rio Guama, 28 Aug 1973, R. T. Schuh, 2 ♂♂ (00138658, 00138734) (USNM). Jacareacanga, 6.2667°S, 57.65°W, 88 m, Jun 1970, F. R. Barbosa, 1 ♂ (00161386) (USNM). **Parana:** Antonia, Reserva Sapitanduva, 05 Oct 1986, Lev. Ent. ProfauPar. lampada, 1 ♀ (00161380) (USNM). **Rio de Janeiro:** Conceicao de Macabu, Aug 1977, M. Alvarenga, 1 ♀ (00138738) (USNM); Sep 1978, Alvarenga, 2 ♂♂ (00161322, 00161323) (USNM). Est do Rio, 1947, J.C.M. Carvalho, 3 ♀♀ (00161991 - 00161993) (USNM). Estrada Rio-Sao Paulo, Km 47, 22.8669°S, 43.7789°W, 12 Nov 1943, O. Braga, 1 ♂ (00174909) (MNRJ). Guaratiba, 22.9667°S, 42.8°W, Nov 1941, J. C. M. Carvalho, 1 ♂ (00161379) (USNM). **Rondonia:** 62 km SW of Ariquemes, near Fzda. Rancho Grande, 10.32921°S, 63.46881°W, 30 Mar 1992 - 10 Apr 1992, J. E. Eger, 1 ♂ (00161338), 2 ♀♀ (00161339, 00161340) (USNM). **Santa Catarina:** Nova Teutonia, 27.18333°S, 52.38333°W, 642 m, Dec 1944, F. Plaumann, 3 ♀♀ (00161381 - 00161383) (USNM); 03 Aug 1951, F. Plaumann, 1 ♀ (00161336) (USNM). **São Paulo:** ***São Paulo Co*.:** km 47, Estr. Rio, 1950, J. Maldonado C., 1 ♂ (00161390) (USNM). **CUBA:** **Havana:** Habana-Alamar-Cojimer, 210 m, 10 Aug 1966 - 24 Aug 1966, Jar. Prokop, 1 ♂ (00161320) (USNM). **Mantanzas:** Varadero, 23.1536°N, 81.2514°W, 31 Jan 1965, Jar. Prokop, 1 ♀ (00161321) (USNM). **HAITI:** **Ouest:** Port-au-Prince, 18.5392°N, 72.335°W, 99 m, Jul 1961, J. Maldonado C., 1 ♂ (00161998) (USNM). **MEXICO:** **Veracruz:** Los Tuxtlas area, Rio Maquinas, “Los Tuxtlas” Biological Station, 31 km NE of Catemaco, 04 May 1981 - 14 May 1981, C. M. & O. S. Flint, Jr., 1 ♀ (00161389) (USNM). Los Tuxtlas area, Rio Palma, below La Palma, “Los Tuxtlas” Biological Station, 31 km NE of Catemaco, 05 May 1981, C. M. & O. S. Flint, Jr., 1 ♀ (00161388) (USNM). Veracruz, 19.18333°N, 96.11666°W, 1 m, Dec 1961, N. L. H. Krauss, 1 ♀ (00161343) (USNM). **NICARAGUA:** **Managua:** Managua, 12.15083°N, 86.26833°W, Baker, 1 ♂ (00161378), 1 ♀ (00161595) (USNM). **PANAMA:** **Canal Zone:** Playa Venado, 30 Jul 1975 - 31 Jul 1975, E. M. & J. L. Fisher, 1 ♂ (00138763) (UCR). **Cocle:** Agua Dulce, 1951, Blanton, 2 ♂♂ (00161328, 00161329) (USNM); 07 Aug 1951, Blanton, 2 ♂♂ (00161326, 00161327), 1 ♀ (00161325) (USNM). Aguadulce, 07 Aug 1951, Blanton, 4 ♂♂ (00161353 - 00161356) (USNM); 25 Sep 1951, F. S. Blanton, 1 ♀ ( 00161344) (USNM). Puerto Obaldia, 04 Nov 1952, collector unknown, 1 ♀ (00161609) (USNM). Rio Hato, 8.3833°N, 80.1667°W, 03 Aug 1953, F. S. Blanton, 1 ♀ (00161319) (USNM). **Darien:** El Real, 8.1333°N, 77.7167°W, 17 m, 19 Mar 1953, F. S. Blanton, 2 ♀♀ (00161995, 00161996) (USNM). Garachine, 8.06472°N, 78.36277°W, 18 Feb 1953, F. S. Blanton, 1 ♀ (00161999) (USNM). **Panama:** Las Cumbres, 9.06°N, 79.32°W, 113 m, 28 Jul 1971, M. Daykin, 1 ♂ (00138698) (UCD). Tocumen, 9.0833°N, 79.3833°W, 25 Mar 1952, F. S. Blanton, 2 ♀♀ (00161341, 00162000) (USNM); 17 Nov 1952, F. S. Blanton, 1 ♀ (00161924) (USNM); 04 Dec 1952, F. S. Blanton, light trap, 1 ♀ (00161923) (USNM); 05 Oct 1953, F. S. Blanton, 1 ♀ (00161342) (USNM). **PARAGUAY:** **Alto Parana:** Reserva Biol. Tati Yupi, 14 Nov 1990, G. Arriagada, 2 ♂♂ (00161345, 00161346), 6 ♀♀ (00161347 - 00161352) (USNM). **Tarija:** ***Gran Chaco Co*.:** 260 kilometers west of the Paraguay River, 13 Jul 1935, Alberto Schulze, 1 ♂ (00161927) (USNM). **PERU:** **La Libertad:** Trujillo, near mouth of Rio Moche, 02 Jul 1972, R.T. and J.C. Schuh, 1 ♀ (00138706) (USNM). **Lima:** Callao, 12.03333°S, 77.13333°W, 3 m, 17 Nov 1950, Michelbacher and Ross, 1 ♀ (00161611) (USNM). **Loreto:** Km 3 Tournavista Rd., 34 km W Pucallpa, 8.48333°S, 74.8°W, 300 m, 13 Dec 1971 - 31 Dec 1971, R. T. & J. C. Schuh, light trap, 63 ♂♂ (00138655 - 00138657, 00138659, 00138671, 00138674 - 00138675, 00138678, 00138680, 00138683, 00138687, 00138691, 00138694, 00138696 - 00138697, 00138699, 00138702, 00138705, 00138712, 00138718, 00138721 - 00138728, 00138730 - 00138733, 00138737, 00138739 - 00138740, 00138744, 00138746 - 00138747, 00138750, 00138752 - 00138753, 00138758 - 00138759, 00138761 - 00138762, 00138764 - 00138767, 00138770 - 00138775, 00138778 - 00138779, 00138782 - 00138786, 00138788), 26 ♀♀ (00138661, 00138663, 00138667, 00138670, 00138676, 00138681, 00138685 - 00138686, 00138688, 00138695, 00138700 - 00138701, 00138704, 00138707 - 00138709, 00138714 - 00138715, 00138717, 00138720, 00138735, 00138751, 00138769, 00138777, 00138780, 00161318) (USNM). Lake Yarinacocha, 10 km NW of Pucallpa, 150 m, 08 Dec 1971 - 10 Dec 1971, R. T. Schuh, light trap, 2 ♀♀ (00138736, 00138741) (USNM); 08 Dec 1971, R. T. Schuh, light trap, 1 ♂ (00138692) (USNM). **Madre de Dios:** ***Tambopata Co*.:** Rio Tambopata Reserve, 30 air km SW Pto. Maldonado, 290 m, 26 Nov 1979 - 30 Nov 1979, J. B. Heppner, subtropical moist forest, 1 ♀ (00161337) (USNM). **PUERTO RICO:** **Caguas:** Caguas, May 1965, Ricardo Jorge, 2 ♂♂ (00161361, 00161362) (USNM). El Verde, Jun 1967, J. Maldonado C., 1 ♂ (00161360), 1 ♀ (00161359) (USNM). **Cayey:** Cayey, Jul 1961, J. Maldonado C., 1 ♂ (00161363), 3 ♀♀ (00138713, 00161364 - 00161365), 1 nymph (00161366) (USNM). **Comerio:** Comerio, 05 Jun 1961, J. Maldonado C., 1 ♂ (00161376) (USNM). **Guanica:** ***Locality unknown Co*.:** Lake Guanica, 23 Jul 1936, H. L. Dozier, 1 ♀ (00161335) (USNM). **Guayanilla:** Guayanilla, Sep 1960 - Nov 1960, E. Murphy, 2 ♀♀ (00161370, 00161371) (USNM). **Maricao:** Maricao, Jul 1960, J. Maldonado C., 1 ♂ (00161375) (USNM). **Mayaguez:** ***Sabanetas Co*.:** Mani Beach, 04 Aug 1935, H. L. Dozier, 1 ♀ (00161334) (USNM). Mayaguez, Mar 1960, J. Maldonado C., 1 ♀ (00161372) (USNM); Jun 1962, J. Maldonado C., 3 ♂♂ (00161367 - 00161369) (USNM); Jul 1975, J. Maldonado C., 1 ♀ (00161373) (USNM). Mayaguez, Dec 1964, R. Ricardo, 1 ♂ (00161357), 1 ♀ (00161358) (USNM). **Rio Grande:** El Yunque, Apr 1967 - Jun 1967, J. Maldonado C., 1 ♀ (00161374) (USNM). **San Juan:** San Juan, 18.46633°N, 66.10573°W, 02 Aug 1914 - 03 Aug 1914, collector unknown, 1 ♀ (00161377) (USNM). Cayey, 18.113°N, 66.166°W, 431 m, 1961, Julio, 1 ♂ (00095356) (AMNH). **UNITED STATES**: **Florida:** ***Highlands Co*.:** Archbold Biological Station, S of Lake Placid, hill area E. of Station, 27.18333°N, 81.34166°W, 01 Jan 2007, W.E. Steiner and J.M. Swearingen, at black light in pine-oak sand scrub, 1 ♀ (00161391) (USNM). **VENEZUELA:** **Aragua:** ***Maracay Co*.:** Maracay, 10 Jul 1968, J. Maldonado C., 1 ♀ (00161387) (USNM). Maracay, 10.24694°N, 67.59583°W, 548 m, 10 Jul 1968, J. Maldonado C., 1 ♂ (00161433) (USNM). **Guanare:** Estado Portuguesa, 10 Sep 1957 - 13 Sep 1957, Borys Malkin, 2 ♂♂ (00161330, 00161331), 2 ♀♀ (00161332, 00161333) (USNM).

### Tytthus pallidus

Henry sp. n.

urn:lsid:zoobank.org:act:E2C46E49-A43D-4C0B-9742-FBAFF41F3C2D

http://species-id.net/wiki/Tytthus_pallidus

Figs 28, 29 160 –163

#### Diagnosis.

This species is recognized by the combination of the dark brown head, pronotum, and scutellum; the pale antennal segment I, with only the base fuscous, the brown antennal segment II; and the uniformly pale yellow legs. All known specimens are macropterous.

*Tytthus pallidus* keys out with *Tytthus piceus* based on the pale antennal segment I and the pale hemelytra with smoky-brown shading. It can be distinguished from *Tytthus piceus* by the broader head, the longer antennal segment I that is longer than the interocular width, and the less prominent calli lacking a glaucous sheen.

#### Description.

*Holotype male* (Fig. 28): Length to apex of hemelytron 2.88 mm, length to base of cuneus 2.21 mm, width across hemelytra 0.88 mm. *Head*: Length 0.35 mm, width across eyes 0.61 mm, interocular width 0.29 mm. *Labium*: Length 1.31 mm. *Antenna*: Segment I length 0.35 mm, II 1.22 mm, III and IV missing. *Pronotum*: Length 0.35 mm, basal width 0.75 mm.

*Coloration*: *Head*: Uniformly dark brown; pale interocular spot found in all other species apparently absent; fuscous to dark reddish brown. *Labium*: Pale brownish yellow. *Antenna*: Segment I uniformly pale yellow, with a dark brown or fuscous ring at base; segments II–IV dark brown. *Pronotum*: Uniformly dark brown. *Mesoscutum*: Uniformly dark brown. *Scutellum*: Uniformly dark brown. *Hemelyton*: Uniformly pale translucent brown. *Ostiolar evaporative area*: Dark brown to fuscous. *Ventral surface*: Thorax dark brown to dark reddish brown; abdomen dark reddish brown on segment II, III, and genital capsule, slightly paler in between. *Legs*: Coxa pale yellow, reddish brown at bases; femora, tibiae, tarsi, and claws uniformly pale yellow.

*Structure, texture, and vestiture*: *Antenna*: Segment I set with a few, scattered, recumbent setae and two erect, subapical, bristlelike setae. *Labium*: Extending beyond metacoxae to abdominal segment II or III. *Pronotum*: Shiny, impunctate; anterior angles rounded; lateral margins weakly concave, flaring at posterior angles; posterior margin distinctly sinuate; calli weakly swollen; set with relatively long, semierect setae. *Mesoscutum*: Impunctate, broadly exposed; with a few scattered semierect setae. *Scutellum*: Weakly shining, impunctate; equilateral; set with scattered, semierect setae. *Hemelytra*: Macropterous, cuneus and membrane fully developed, extending well beyond apex of abdomen.

*Male genitalia*: *Left paramere* (Fig. 160): Mitt-shaped; right arm long, broad, apically blunt; left arm short, apically acute. *Right paramere* (Fig. 161): Round. *Endosoma*: Teneral and damaged; not drawn. *Phallotheca* (Fig. 162): slender, apically acute.

*Female* (n = 4) (Fig. 29): Length to apex of hemelytron 2.69–3.33 mm, length to base of cuneus 2.05–2.40 mm, width across hemelytra 0.82–1.02 mm. *Head*: Length 0.32–0.38 mm, width across eyes 0.56–0.64 mm, interocular width 0.29–0.30 mm. *Labium*: Length 1.12–1.44 mm. *Antenna*: Segment I length 0.26–0.34 mm, II 0.85–1.23 mm, III 0.61–0.78 mm, IV 0.51–0.56 mm. *Pronotum*: Length 0.29–0.37 mm, basal width 0.69–0.85 mm.

**Figures 163–170.**
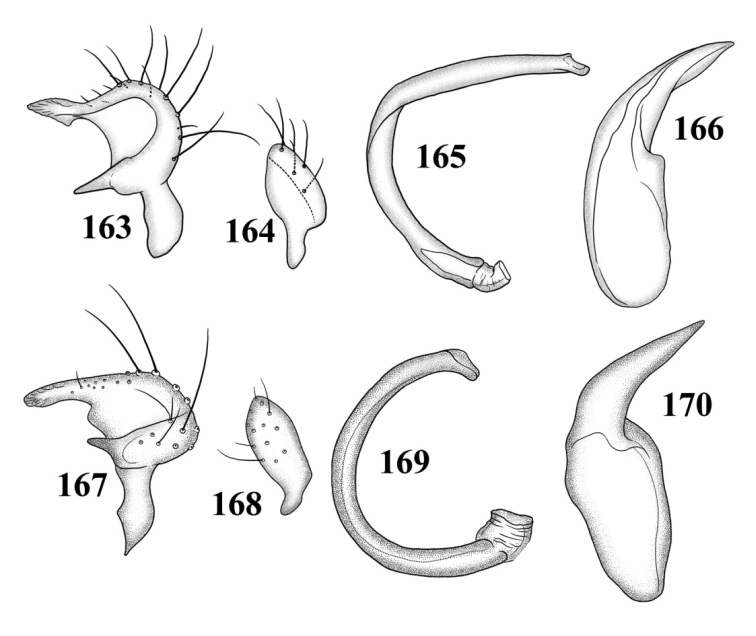
Male genitalia **163–166** *Tytthus parviceps* **163** left paramere **164** right paramere **165** endosoma **166** phallotheca **167–170** *Tytthus piceus* **167** left paramere **168** right paramere **169** endosoma **170** phallotheca.

#### Etymology.

The specific epithet “pallidus” refers to the characteristic pale yellow first antennal segment.

#### Host.

Unknown.

#### Distribution.

Known from Brazil and Panama.

#### Type material.

**Holotype** ♂ (00162199) (USNM): **PANAMA:** **Darien: [**Pinogana District], El Real, 19 Mar. 1953, F. S. Blanton, 1 ♂ (USNM). El Real, 08 Aug 1952, F. S. Blanton, 2 ♀♀ (00161392, 00161393) (USNM). **Paratypes:** **BRAZIL: Amazonas:** Reserva Ducke, 25 km NNE of Manaus, 120 m, 26 Jul 1973, R.T. Schuh, 2 ♀♀ (00165829) (AMNH).

### Tytthus panamensis

Carvalho & Southwood

http://species-id.net/wiki/Tytthus_panamensis

Figs 30, 31

Tytthus panamensis Carvalho and Southwood 1955: 32 (orig. descrip.); Carvalho 1958: 158 (cat.); Carvalho and Afonso 1977: 9 (list); Carvalho and Froeschner 1987: 218 (list, type info.); Schuh 1995: 249 (cat.); Froeschner 1999: 133 (list, note).

#### Diagnosis.

*Tytthus panamensis* is a small species distinguished by the combination of the fuscous head, pronotum, and scutellum; the fuscous antennal segment I, with only the apex pale; the pale yellowish brown antennal segment II; and the uniformly pale legs. All known specimens are macropterous.

It is most similar to *Tytthus pallidus*, sp. n. in overall size and color, including the reduced or apparent lack of an interocular spot on the head and the uniformly pale legs. It differs in having a dark brown antennal segment I, with only the apex pale, and a pale brown antennal segment II, whereas *Tytthus pallidus* has a pale antennal segment I, with only a narrow dark ring at the base, and segment II is dark brown. *Tytthus panamensis* keys near *Tytthus vagus* based on the dark antennal segment I with only the apex pale, but can be distinguished by the uniformly pale yellowish brown antennal segment II and hind femur.

#### Description.

*Male* (n =1, plus holotype in parentheses) (Fig. 30): Length to apex of hemelytron 2.21 mm (2.37 mm), length to base of cuneus 1.57 mm (1.63 mm), width across hemelytra 2.21 mm (2.37 mm). *Head*: Length 0.26 mm (0.29 mm), width across eyes 0.48 mm (0.51 mm), interocular width 0.24 mm (0.26 mm). *Labium*: Length 0.85 mm (0.93 mm). *Antenna*: Segment I length 0.30 mm (0.35 mm), II 0.88 mm (0.99 mm), III missing (0.56 mm), IV missing (missing). *Pronotum*: Length 0.22 mm (0.22 mm), basal width 0.58 mm (0.66 mm).

*Coloration*: *Head*: Uniformly dark brown, pale interocular spot found in all other species (except *Tytthus pallidus*) apparently absent; eyes dark brown to fuscous. *Labium*: Uniformly pale yellow. *Antenna*: Segment I dark brown, narrowly pale at apex; segment II pale yellowish brown; segments III and IV brown. *Pronotum*: Uniformly dark brown. *Mesoscutum*: Uniformly dark brown. *Scutellum*: Uniformly dark brown. *Hemelytron*: Uniformly pale translucent brown. *Ostiolar evaporative area*: Reddish brown. *Ventral surface*: Thorax and abdomen uniformly dark reddish brown. *Legs*: Procoxae pale yellow; meso- and metacoxae pale yellow, with bases reddish brown; femora, tibiae, tarsi, and claws uniformly pale yellow.

*Structure, texture, and vestiture*: *Head*: Shiny, impunctate; buccula slender, tapering posteriorly, ending near level with middle of eye. *Labium*: Extending to apices of meso- or bases of metacoxae; segment I extending beyond base of head to anterior edge of xyphus before procoxae. *Antenna*: Segment I with relatively sparse, short, recumbent setae and two, long, subpical, bristlelike setae; segment II thickly set with short, recumbent setae, intermixed with a few more scattered, semierect setae. *Pronotum*: Shiny, impunctate; anterior angles weakly rounded; lateral margins straight, gradually widening to posterior angles; basal margin weakly sinuate; set with scattered recumbent and semierect setae. *Mesoscutum*: Shiny, impunctate, broadly exposed; set with a few short, semierect setae. *Scutellum*: Shiny, impunctate, equilateral; set with a few scattered, short, recumbent and semierect setae. *Hemelytron*: Macropterous, cuneus and membrane fully developed, extending posteriorly well beyond apex of abdomen; evenly set with relatively long, recumbent setae.

*Male genitalia*: Not examined. See note below.

*Female* (n = 1) (Fig. 31): Length to apex of hemelytron 2.91mm, length to base of cuneus 1.98 mm, width across hemelytra 0.80 mm. *Head*: Length 0.34 mm, width across eyes 0.59 mm, interocular width 0.29 mm. *Labium*: Length 1.20 mm. *Antenna*: Missing. *Pronotum*: Length 0.27 mm, basal width 0.74 mm.

Similar to male in overall appearance, differing primarily in the broader body form.

#### Host.

Unknown.

#### Distribution.

Described and known only from Panama.

#### Discussion.

The one male paratype previously had been dissected and the genitalia apparently were placed in a glass genitalia vial sealed with a cork stopper. Since that dissection, the cork has dried and crumbled and the genitalia are missing from the vial. I now have placed the glass vial inside a polyethylene vial, sealed it with a neoprene stopper, and reattached it to the specimen pin.

#### Type material examined.

**Holotype** ♂ (00162200) (USNM): **PANAMA:** **Canal Zone:** Corozal, 8.98844°N, 79.5719°W,14 Apr 1912, A. Busck, at light. **Paratypes**: Same data as for holotype, 1 ♂ (00161395) (USNM), 1 ♀ (00161394) (USNM).

#### Other specimens examined.

**PANAMA:** **Canal Zone:**. Fort Gulick, 9.31667°N, 79.86667°W, 21 Aug 1952, F. S. Blanton, 1 ♀ (00162201) (USNM). **Darien:** Garachine, 8.06472°N, 78.36277°W, 17 Feb 1953, F. S. Blanton, 1 ♀ (00161396) (USNM).

### Tytthus parviceps

(Reuter)

http://species-id.net/wiki/Tytthus_parviceps

Figs 32–34 163–166

Cyrtorhinus parviceps Reuter 1890: 258 (orig. descrip.); Carvalho 1952: 80 (list).
Cylloceps pellicia Uhler 1893: 712 (orig. descrip.); Barber 1914: 500.; Van Duzee 1917: 418; (cat.); Blatchley 1926: 860 (descrip.); Knight 1927b: 105 (n. comb.). Synonymized by China 1924: 444; lectotype designated below.
Cyrtorhinus parviceps var. *thoracicus*Horváth 1909: 294 (orig. descrip.); Oshanin 1910: 836 (cat.); Poppius 1914: 70 (descrip.); China 1924: 444 (list); Lindberg 1953: 126 (list). Lectotype designated by Kerzhner 1996: 102.
Cyrtorhinus pygmaeus : Van Duzee 1917: 824 (misident.).
Cyrtorhinus pygmaeus : Blatchley 1926: 853 (descrip., misident.); Barber 1954: 15 (list).
Tytthus pygmaeus form *intermedia*Stichel 1956: 272 (orig. descrip.). Synonymized by Schuh 1995: 249.
Cyrtorhinus pellicia : Ingham et al. 1995: 73 (note).
Tytthus parviceps : Carvalho and Southwood 1955: 21 (descrip., n. comb.); Carvalho 1956: 41 (descrip., hosts) 1958: 158 (cat.); Schmitz 1976: 502; Nguyen et al. 1984: 1 (note); Henry and Wheeler 1988: 458 (cat.); Henry and Hilburn 1990: 681 (distr., host); Linnavuori 1993: 141 (distr.); Schuh 1995: 249; Linnavuori and Van Harten 1997: 188 (distr., host); Carapezza 1997: 108 (distr.), 1998: 37 (distr., fig.); Kerzhner and Josifov 1999: 441 (cat.); Froeschner 1999: 133 (list); Wheeler 2000: 665 (hosts, biol., review) 2001: 278 (hosts, biol., review); Hernández and Henry 2010: 123 (diag., hosts).

#### Diagnosis.

This widespread circumtropical species is best distinguished by the pale yellow anterior area of the pronotum on almost all specimens, a characteristic found only in some specimens of *Tytthus chinensis* and certain color forms of *Tytthus pygmaeus*. It is also recognized by the combination of the black head, with large, yellow interocular spots; black antennal segment I, with the apex and base of segment I narrowly pale, yellowish-brown to brown segment II with the base black (but often uniformly fuscous or black); black scutellum, pale translucent hemelytra; and uniformly pale yellow legs, with a fuscous knee spot on each tibia. *Tytthus parviceps* is readily distinguished from *Tytthus pymaeus* by the knee spots at the bases of the tibiae. From *Tytthus chinensis* it is distinguished by the usually extensively yellow anterior third to one half of the pronotum and the more strongly C-shaped (Fig. 165) endosoma. Only macropterous individuals of this species are known.

#### Description.

*Male* (n = 10) (Fig. 32): Length to apex of hemelytron 2.11–2.46 mm, length to base of cuneus 1.50–1.70 mm, width across hemelytra 0.74–0.82 mm. *Head*: Length 0.26–0.27 mm, width across eyes 0.53–0.56 mm, interocular width 0.27–0.29 mm. *Labium*: Length 0.82–0.86 mm. *Antenna*: Segment I length 0.24–0.26 mm, II 0.72–0.80 mm, III 0.42–0.48 mm, IV 0.37–0.40 mm. *Pronotum*: Length 0.27–0.30 mm, basal width 0.67–0.74 mm.

*Coloration*: *Head*: Uniformly fuscous to black, with a large, yellow, interocular spot touching inner margin of each eye, spots nearly contiguous in some individuals; eyes fuscous to dark reddish brown. *Labium*: Uniformly pale yellow, except brown apical half of segment IV. *Antenna*: Segment I black, with the apex pale yellow; segment II yellowish brown to brown, with base black but sometimes uniformly black; segments III–IV uniformly fuscous to black. *Pronotum*: Anterior half typically broadly yellow from margin to margin, sometimes yellow area reduced so lateral margins and area between calli become invaded with fuscous, to the extreme with yellow greatly reduced or absent; posterior half uniformly fuscous to black. *Mesoscutum*: Uniformly yellowish brown to fuscous. *Scutellum*: Uniformly fuscous to black. *Hemelytron*: Uniformly translucent yellow. *Ostiolar evaporative area*: Uniformly yellow to yellow with central area of auricle invaded by fuscous. *Ventral surface*: Anterior half of proacetabula yellow, propleura, pro- and mesosterna black; metapleura yellowish, invaded by fuscous; abdomen largely yellowish, with only genital capsule fuscous or black. *Legs*: Uniformly yellow, with only bases of tibiae fuscous.

*Structure, texture, and vestiture*: *Head*: Weakly shiny, impunctate; buccula slender, extending posteriorly, ending near level with hind margin of eye; thickly set with short to relatively long semierect setae, especially on frons. *Labium*: Extending to apices of meso- or bases of metacoxae; segment I extending beyond base of head to xyphyus just before procoxae. *Antenna*: Segment I set with short, recumbent setae and two, long, subapical, bristlelike setae; segment II thickly set with short, recumbent setae, intermixed with row of longer, erect setae (similar to *Tytthus chinensis*) along ventral surface. *Pronotum*: Anterior angles rounded; lateral margins weakly concave, gradually widening to rounded posterior angles; posterior margin weakly sinuate. *Mesoscutum*: Weakly shiny, impunctate; set with a few scattered, semierect setae. *Scutellum*: Weakly shiny, impunctate; equilateral; set with a few scattered recumbent and semierect setae. *Hemelytron*: Macropterous, cuneus and membrane fully developed, extending posteriorly well beyond apex of abdomen; evenly set with relatively long, recumbent setae.

*Male genitalia*: *Left paramere* (Fig. 163): Mitt-shaped; right arm long, broad, almost triangular, bluntly pointed apically; left arm relatively long, apically pointed. *Right paramere* (Fig. 164): Oval. *Endosoma* (Fig. 165): Strongly C-shaped, blunt apically. *Phallotheca* (Fig. 166): Slender, apically acute.

*Female* (n = 10) (Figs 33, 34): Length to apex of hemelytron 2.40–3.04 mm, length to base of cuneus 1.79–2.21 mm, width across hemelytra 1.02–1.09 mm. *Head*: Length 0.29–0.30 mm, width across eyes 0.59–0.64 mm, interocular width 0.30–0.32 mm. *Labium*: Length 0.88–1.04 mm. *Antenna*: Segment I length 0.26–0.29 mm, II 0.69–0.86 mm, III 0.43–0.56 mm, IV 0.43–0.50 mm. *Pronotum*: Length 0.34–0.38 mm, basal width 0.86–0.98 mm.

Similar to male, differing primarily in the larger size and broader body form.

#### Hosts.

In the New World, *Tytthus parviceps* has been associated with the delphacid *Saccarosydne saccharivora* (Westwood) (Carvalho and Southwood 1955) and the sugarcane delphacid, *Perkinsiella saccaricida* Kirkaldy (Sosa 1985), on sugarcane in Florida and Ecuador. It also has been taken on *Spartina alterniflora* Loisel [Poaceae] in North Carolina (Wheeler and Henry 1992). In the Old World, it is known to prey on eggs of the brown plant hopper, *Nilaparvata lugens* (Stål), in India and sugarcane delphacid in Australia (Bull 1981).

#### Distribution.

In the New World, this widespread circumtropical and subtropical species is known from Central and South America (see specimen data below), the West Indies, Bermuda, and Florida and North Carolina in the United States (Henry and Hilburn 1990, Wheeler and Henry 1992). In the Old World, it has been reported from southern Europe, the Middle East, Africa, eastern (Far-Eastern Russia, Korea) and southeastern Asia, Australia, India, and islands in the Indian, Pacific, and southern Atlantic oceans (Wheeler and Henry 1992, Kerzhner and Josifov 1999). Cassis and Gross (1995) omitted this species from the Australian list, suggesting that reports in the literature should be referred to the similar-appearing *Tytthus chinensis*.

#### Discussion.

*Tytthus parviceps*, described from Egypt, is similar to *Tytthus chinensis* in nearly all external characters, except for the strongly pale yellow anterior area around the calli of the pronotum, which may cause considerable confusion between these species when individuals of *Tytthus parviceps* with greatly reduced yellow markings or an entirely dark pronotum are encountered, or when specimens of *Tytthus chinensis* with more extensive yellow markings are found. Dissection of representative males of *Tytthus parviceps* from different regions (list localities) shows that the endosoma is consistently S-shaped, whereas specimens of *Tytthus chinensis* from (list localities) have a C-shaped endosoma.

China (1924) synonymized *Cylloceps pellicia* Uhler with *Cyrtorhinus parviceps* after examining the “type-specimen” deposited in the Natural History Museum (BMNH). I have examined the female syntype of *Cyrtorhinus parviceps* from Egypt and a female syntype of *Cylloceps pellicia* from St. Vincent in the Natural History Museum, as well as a male syntype of *Capsus pellicia* from Cuba (labeled in Uhler’s hand) in the USNM collection, and agree that Uhler’s species is a junior synonym of *Tytthus parviceps* as now defined.

Also, Josifov and Kerzhner (1972), in describing *Tytthus koreanus*, initially indicated that it was similar to *Tytthus parviceps* (Reuter) based on the extensive pale areas on the pronotum. However, they also added that the new species might be conspecific with *Tytthus chinensis* described from Hong Kong, which according to the original description (Stål 1860) and to Reuter (1903) is a larger species (3.00 mm) than *Tytthus parviceps*. Unfortunately, the type of *Tytthus chinensis* is lost and the current concept of this species was established by Carvalho and Southwood (1955) and Carvalho (1956) as smaller, with the pronotum entirely black. I have examined a large amount of material from Australia and the Indo-Pacific Region that contains a few individuals among mostly black specimens exhibiting a yellow pronotal color not too unlike that described by Josifov and Kerzhner (1972) for *Tytthus koreanus*. In addition, I have studied three specimens from Korea that also have more yellow on the anterior area of the pronotum but less than for typical specimens of *Tytthus parviceps*. As a consequence, I am treating *Tytthus koreanus* as a junior synonym of *Tytthus chinensis* (which see) until additional studies can show otherwise.

#### Type material examined.

To ensure nomenclatural stability, I am selecting the following syntypes as lectotypes:

*Cyrtorhinus parviceps* ♀ (BMNH): label 1 (circular label with orange ring) “Type”; label 2, “Le Cairo, V. 86, E. Antian, Schweinfor”; label 3 “Cyrtorhinus parviceps Reut. type; label 4 (here added, red printed label), “**LECTOTYPE**: ♀, *Cyrtorhinus parviceps* Uhler, by T. J. Henry”; label 4 “00085519 (BMNH).

*Cylloceps pellicia*: ♂ (USNM): label 1, “Cuba”; label 2 (handwritten), “152”; label 3, “PR Uhler Collection”; label 4 (handwritten), “Cylloceps pellicia Cuba: Uhler”; label 5 (handwritten, except “Det. Uhler”), “Cylloceps pellicia Uhler Cuba [printed] Det Uhler”; label 6 (name handwritten; remainder printed), “Tytthus parviceps (Rt.) det JCMCarvalho)”; label 7 (here added, printed red label), “**LECTOTYPE**: ♂, *Cylloceps pellicia* Uhler, by T. J. Henry.” A female from “St. Vincent, May, H. H. Smith” in the BMNH with 00085520 (BMNH) is here designated as a paralectotype.

#### Other specimens examined.

**ANTIGUA AND BARBUDA:** **Antigua:** Galleon Beach, 03 Aug 1992, H.V. & R.M. Baranowski, 2 ♀♀ (00161495, 00161496) (USNM). **BAHAMAS:** **Andros Island:** Fresh Creek, 24.7°N, 77.76666°W, 23 Apr 1953, E.B. Hayden, 1 ♂ (00165901) (plus 10 ♂♂, 3 ♀♀) (AMNH). Mangrove Cay, 24.28333°N, 77.66666°W, 26 Apr 1953, E.B. Hayden, 12 ♂♂ (00165889 - 00165900), 6 ♀♀ (00165861 - 00165866) (AMNH). **Bimini:** South Bimini Island, 25.70623°N, 79.28204°W, 6 m, Jun 1951, Cazier and C. & P. Vaurie, 1 ♀ (00165913) (AMNH). **Exuma Cays:** Staniard Cay, 24.18333°N, 76.41666°W, 13 Jan 1953, E. B. Hayden & L. Giovannoli, 9 ♂♂ (00165876 - 00165884), 7 ♀♀ (00165867 - 00165873) (AMNH). **Nassau:** ***New Providence Co*.:** New Providence Island, 03 Jan 1953, E.B. Hayden, 1 ♀ (00165874) (AMNH). **San Salvador Island:** nr. Cockburn Town, 24.0333°N, 74.5167°W, 18 Mar 1953, L. Giovannoli & G.B. Rabb, 4 ♂♂ (00165885 - 00165888), 1 ♂ (00165875) (AMNH). **BARBADOS**: H. H. Smith, 1 ♂ (BMNH). **BELIZE:** **Belize:** Big Falls Ranch, 23 Dec 1975, R. Akers, *Oryza sativa* (Poaceae), 1 ♂ (00161546) (USNM). **BERMUDA:** **Devonshire Parish:** Devonshire Marsh, 13 Jan 1988, T. J. Henry, 1 ♂ ( 00161503) (USNM); 14 Jul 1988 - 22 Jul 1988, M.R. Wilson & D.J. Hilburn, 1 ♂ (00161502) (USNM). **Paget Parish:** Paget Marsh, 23 Jul 1971, N. L. H. Krauss, 1 ♂ (00161505) (USNM); 13 Jan 1988, T. J. Henry, 1 ♀ (00161504) (USNM); 14 Jul 1988 - 22 Jul 1998, M.R. Wilson & D.J. Hilburn, 15 ♂♂ (00161468 - 00161482), 3 ♀♀ (00161483 - 00161485) (USNM) (an additional 10 ♂♂, 5 ♀♀ in BMNH). **BRAZIL:** **Para:** ***Santarem Co*.:** Taperinha, 11 Jun 1927 - 20 Jun 1927, Zerny, 1 ♀ (00161614) (USNM). **BURKINA FASO:** **Kadiogo:** Upper Volta, Ouagadougou, 03 Nov 1973 - 05 Nov 1973, R. Linnavuori, 1 ♂ (00166054), 3 ♀♀ (00166055 - 00166057) (AMNH). **CAMEROON:** Locality unknown, 1983, Irct A. Renou, 3 ♂♂ (00161707 - 00161709), 4 ♀♀ (00161705 - 00161706, 00161767 - 00161768), 2 nymphs (00161710, 00161711) (USNM). **CAYMAN ISLANDS:** **Grand Cayman:** Grand Cayman, 05 Aug 1988, P. Fitzgerald, 1 nymph (00161587) (USNM). **CHAD:** Farcha, 20 May 1973 - 22 May 1973, R. Linnavuori, 1 ♀ (00166052) (AMNH). **CHILE:** **Vicuna:** El Pangue, Nov 1961, L.E. Peña, 2 ♂♂ (00161464, 00161465), 1 ♀ (00161466) (USNM). **COSTA RICA:** **Guanacaste:** 10 mi NW of Liberia, 10.723°N, 85.344°W, 25 Jul 1965, Paul J. Spangler, 1 ♀ (00161586) (USNM). **CUBA:** **Cienfuegos:** La Jiquima, 29 Jul 1956, J. M. Osorio, 2 ♂♂ (00161519, 00161520), 6 ♀♀ (00161521 - 00161526) (USNM). **La Habana:** Alamar-Cojimar, 10 m, 04 May 1966 - 05 May 1966, Jar. Prokop, 1 ♂ (00161463) (USNM). Habana-Marianao, 15 m, 15 Jun 1966 - 20 Jul 1966, F. Gregor, 1 ♂ (00161462) (USNM). **Pinar del Rio:** La Jiquima, 27 Jul 1956, J. Acuna, 5 ♂♂ (00161729 - 00161730, 00161539, 00161729 - 00161730), 6 ♀♀ (00161540 - 00161544, 00161728) (USNM). **DEMOCRATIC REPUBLIC OF THE CONGO:** **Haut-Katanga:** Lubumbashi, 10 Apr 1971 - 11 Apr 1971, R. Linnavuori, 1 ♂ (00166024) (AMNH); 25 Apr 1971 - 26 Apr 1971, R. Linnavuori, 1 ♂ (00166023) (AMNH). **Nord-Kivu:** Lac Edouard, 15 Jan 1936, Dr. H. Damas, 1 ♂ (00161766) (USNM). **DOMINICAN REPUBLIC:** **La Altagracia:** Nisibon, 03 May 1978, Woodruff & Fairchild, 1 ♀ (00161584) (USNM). Nisibon, Finca Papagayo, 46 m, 04 Apr 2000 - 07 Apr 2000, T. J. Henry and R. E. Woodruff, 5 ♂♂ (00161486 - 00161490), 3 ♀♀ (00161491 - 00161493) (USNM). **Pendernales:** Cabo Rojo, Alcoa Headquarters, 13 Jun 1988, R. E. Woodruff, 1 ♀ (00161583), 1 ♀ (00161582) (USNM). **Puerto Plata:** Rio Camu, 19 km NE of Jarabacoa, 12 Jun 1969, Flint & Gomez, 1 ♀ (00161591) (USNM). **San Juan:** Locality unknown, 18.88°N, 71.27°W, 19 Oct 1967, collector unknown, 1 ♀ (00161550) (USNM). **San Pedro de Macoris:** Santo Domingo, 8 mi up Macoris river, 16 Jul 1917, Harold Morrison, 3 ♂♂ (00161536 - 00161538) (USNM). **ECUADOR:** **El Oro:** Victoria-Arenillas, 150 m, 18 Aug 1977 - 19 Aug 1977, L. Pena G., 1 ♂ (00161580) (USNM). **Guayas:** Ingenio San Carlos, 29 Nov 1995, Walter Gordillo, 1 ♂ (00161547), 2 ♀♀ (00161548, 00161549) (USNM). **Imbabura:** Otavalo, 01 Jan 1970, R. Levi-Castillo, 1 ♂ (00161531), 2 ♀♀ (00161530, 00161610) (USNM). **Pichincha:** Ingenio San Carlos, 07 May 1982, Robert Morey, 1 ♂ (00161721), 1 ♀ (00161722) (USNM). **EGYPT:** **6th of October Governorate:** W. Desert, Bahariya Oasis, 12 May 1995, Dr. Ullrich, 1 ♂ (00161648) (USNM); 30 Apr 1996, Dr. Ullrich, 2 ♂♂ (00161651, 00161652), 2 ♀♀ (00161649, 00161650) (USNM). **Aswan:** Aswan, 24.0875°N, 32.8989°E, 25 Dec 1993, Dr. Ullrich, 2 ♂♂ (00161572, 00161573), 1 ♀ (00161571) (USNM). **Cairo Governorate:** 25 km SW of Cairo, Wadi Digla, 23 Jan 1998, Dr. Ullrich, 1 ♂ (00161575), 1 ♀ (00161576) (USNM). Cairo, Digla, Road to Wadi Digla, 29.95861°N, 31.32041°E, 01 Feb 1998, Dr. Ullrich, 4 ♂♂ (00161657 - 00161660), 3 ♀♀ (00161661 - 00161663) (USNM); 11 Feb 1998, Dr. Ullrich, 1 ♂ (00161577), 1 ♀ (00161616) (USNM). Dahshur, ca. 23 km S. Cairo, 29.79908°N, 31.24625°E, 29 May 1996, Dr. Ullrich, 12 ♂♂ (00161617 - 00161624, 00161632 - 00161633, 00161645 - 00161646), 17 ♀♀ (00161625 - 00161630, 00161634 - 00161644), 1 nymph (00161631) (USNM). El-Marg, 02 Apr 1912, Alfieri, 1 ♂ (00161654) (USNM). Maadi, 05 May 1931, Alfieri, 1 ♂ (00161655) (USNM); 29 Aug 1992, Dr. Ullrich, 1 ♀ (00161647) (USNM); 28 May 1996, Dr. Ullrich, 1 ♀ (00161653) (USNM). Meadi, 12 June 1961, R. Linnavuori, 1 ♂ (BMNH). Umgeb, Cairo, 1-19 June 1961, R. Linnavuori, 1 ♀ (BMNH). **Fayum Governorate**: Fayum, 13-14 June 1961, R. Linnavuori, 2 ♀♀ (BMNH). **Qahirah:** Cairo (‘Le Caire,’ ‘Al Qahirah’), 30.05°N, 31.25°E, May 1888, Schweinfurth, syntype, 1 ♀ (00085519) (BMNH). Meadi, 29.9667°N, 31.25°E, 20 m, 06 May 1931, H. Priesner, 1 ♂ (00235057) (ZISP). **EL SALVADOR:** **La Libertad:** La Libertad, 10 m, 29 Oct 1965, N. L. H. Krauss, 1 ♀ (00161589) (USNM). **GHANA:** Accra, 27 Sep 1943 - 29 Sep 1943, M.A. Locke, 1 ♂ (00161780), 3 ♀♀ (00161781 - 00161783) (USNM). **GRENADA:** Balthazar, Windward side, 07 Aug 1963, O. S. Flint, 1 ♀ (00161588) (USNM). **GUATEMALA:** **San Marcos:** 17.3 km SE of Talisman, Rio Cabuz at Hwy. CA2, 14.85°N, 92.06666°W, 200 m, 23 May 1973, G. F. Hevel, 1 ♀ (00161585) (USNM). **GUYANA**: Mibicury, 26 Feb 1998, Srinivasan, on “paddy plant hopper,” 8 ♂♂, 18 ♀♀ (BMNH). Demerara (?), Sahr, C. B. Williams, 1910, 1 ♀ (BMNH). **HAITI**: Gonave Id., 21 Jul 1931, B. M. 1931-448, 2 ♂♂, 1 ♀ (BMNH). **INDIA:** **Manipur:** Delhi, 01 Jan 1950, P.K. Pathek, 2 ♂♂ (00161701, 00161702), 2 ♀♀ (00161703, 00161704) (USNM). **Orrisa**: Cuttack, CRRI, 1980, “predatory on brown planthopper,” 3 ♂♂, 1 ♀ (BMNH). **IRAN (ISLAMIC REPUBLIC OF):** **Hormozgan:** Rodan, 27 Mar 2001 - 29 Mar 2001, R. & S. Linnavuori, 3 ♂♂ (00161675 - 00161677), 1 ♀ (00161678) (USNM). **Khuzestan:** Dezful Research Station, garden, 30 Oct 1971 - 10 Nov 1971, L. Knutson, 1 ♂ (00161656) (USNM). Hormozgan Garband, 23-25 Feb. 2001, 26 Mar., 4 Apr. 2001, R. Linnavuori, 13 ♂♂, 11 ♀♀ (NMW). Harmozagan Oeshm Ramkan, 14-15 May 2002, R. Linnavuori, 1 ♂ (NMW). **ISRAEL:** **Mehoz HaZafon (Northern region):** Dan, 33.21666°N, 35.65°E, 07 Jul 1958, R. Linnavuori, 1 ♂ (00235058) (ZISP). Deganya, 32.7°N, 35.56666°E, 23 Jul 1958, R. Linnavuori, 1 ♀ (00235173) (ZISP).N. Distr., Bet-Savoa Res., 8-9 Aug. 1986, R. Linnavuori, 1 ♂, 3 ♀♀ (NMW). N. Distr., Dan, 11 June 1986, R. Linnavuori, 1 ♀ (NMW). S. Distr. Melot hakikkar, 16-20 Jul. 1986, R. Linnavuori, 1 ♂, 1 ♀ (NMW). S. Distr., Nahal ‘Aruoot, 22 Jul. 1986, R. Linnavuori, 9 ♂♂, 6 ♀♀ (NMW). Yad Hashmone, 10 June 1986, Linnavuori, 1 ♀; S. Distr., Be’er Shev’, June 1986, R. Linnavuori, 2 ♀♀ (NMW). S. Distr., Revivim, 4 June 1986, R. Linnavuori, 1 ♀, (NMW). S. Distr., ‘En Gedi, 28 Apr. 1986, R. Linnavuori, 3 ♂♂, 7 ♀♀ (NMW). S. Distr., ‘Ein Avdat, 10 Aug. 1986, R. Linnavuori, 1 ♀ (NMW). S. Distr., ‘En Besor, 6 Jul. 1986, R. Linnavuori, 8 ♂♂, 3 ♀♀ (NMW). S. Distr., Be’er Sheva’, May 1986, R. Linnavuori, 1 ♂, 2 ♀♀ (NMW). S. Distr., Na’ot hakikkar, 16-20 Jul. 1986, R. Linnavuori, 1 ♂ (NMW). S. Distr., Eliias nr Imma, 23 June 1986, R. Linnavuori, 1 ♂, 1 ♀ (NMW). **IVORY COAST**: Gouméré, 19 Sept. 1973, R. Linnavuori, 3 ♂♂, 6 ♀♀ (NMW). Foro Foro, 25-28 Sept. 1973, R. Linnavuori, 6 ♂♂, 8 ♀♀ (NMW). Lamto, 8-9 Oct. 1973, R. Linnavuori, 1♀ (NMW); Adiopodoumé, 29 Sept.-7 Oct. 1973, R. Linnavuori, 1 ♀ (NMW). Man, 14-21 Oct. 1973, R. Linnavuori, 2 ♂♂, 2 ♀♀ (NMW). **JAMAICA:** **Clarendon Parish:** Monymusk Estate, Jul 1959, F. D. Bennett, (Poaceae), 1 ♀ (00161532) (USNM); 10 Dec 1959, F. D. Bennett, (Poaceae), 1 ♂ (00161535), 2 ♀♀ (00161533, 00161534) (USNM). J. Faradane, B. M. 1970-455, 1 ♂, 3 ♀♀ (BMNH). **MAURITIUS**: Bel Etan, 3 Oct 1957, J. R. Williams, on sugarcane, 3 ♂♂, 1 ♀ (BMNH). **MEXICO:** **Tamaulipas:** Tampico, 22.2167°N, 97.85°W, 2 m, 29 Dec 1908, collector unknown, 1 ♂ (00161402) (USNM). **Yucatan:** Progreso, 21.28°N, 89.67°W, 01 Apr 1992, J. R. Vockeroth, 1 ♂ (00112062), 1;f (00166921) (CNC). **NICARAGUA:** **Granada:** Santa Lastenia, Malacatoya, 08 Nov 1971, Ev. Vogel, 4 ♀♀ (00161513 - 00161516) (USNM). Locality unknown, Nov 1989, J. M. Maes, 10 ♂♂ (00161596 - 00161605), 2 ♀♀ (00161606, 00161607) (USNM). **NIGERIA:** **Lagos**: Ikoyi, 25 Oct 1975, J. Riley, 2 ♀♀ (BMNH). NE St. Yola, 25 Aug. 1973, R. Linnavuori, 2 ♂♂, 4 ♀♀ (NMW). Tahoua- In Waggeur, 12-13 Nov. 1973, R. Linnavuori, 4 ♂♂, 2 ♀♀ (NMW). Kw. St. nr. Sinau, 4 Sept. 1975, R. Linnavuori, 1 ♂, 2 ♀♀ (NMW). N. C. St., Malumfashi, 26-30 Jul. 1973, R. Linnavuori, 3 ♂♂, 9 ♀♀ (NMW). W. St. Ife, 7-8 Jul., 14 Aug. 1973, R. Linnavuori, 1 ♀. NW St. Badeggi, 8-9 Aug. 1973, R. Linnavuori, 1 ♂ (NMW). Oyo State, Ibadan, INTA 1994, C. T. Williams & S. Okhidie, from filtering irrigated lowland rice, 1 ♂ (NMW). Niamey, 9 Nov. 1973, R. Linnavuori (NMW), 1 ♂, 5 ♀♀. Ile-Ife, 5 Aug. 1969, J. T. Medler, 2 ♂♂, 1 ♀ (NMW). N. Bussa K state, 12 Jan. 1970, J. T. Medler, 1 ♀ (NMW). B. Pl. St., nr. Makurdi, 30 Aug. 1973, R. Linnavuori, 3 ♂♂, 3 ♀♀ (NMW). **Kwara:** Shaganu Biological Station, 20 Jul 1973 - 22 Jul 1973, R. Linnavuori, 1 ♂ (00166053) (AMNH). **NE State:** Serti, 20 Aug 1975, R. Linnavuori, 1 ♂ (00161733), 1 nymph (00161734) (USNM). Yola, 25 Aug 1973, R. Linnavuori, 1 ♂ (00161731), 1 ♀ (00161732) (USNM). **Oyo**: Ibadan, Oct 1982, C.I.E., on rice, 1 ♂, 1 ♀ (BMNH). **OMAN**: Wadi Ghul, 1450 m, 23E14'N,57E09'E, 1 Nov. 1990, M. D. Gallagher & J. C. Deeming, 1 ♂ (NMW). DhoPar, Ain Hamran, streamside vegetation, 10 Oct. 1990, J. C. Deeming, 2 ♂♂ (NMW). **PALESTINE TERRITORY[?]**: **West Bank**: Beit-el-Ghofr, N of Haz, about 16 mi NW of San’a, ca 9,300 ft, 4 Feb. 1938, from a lucerne field, 1 ♂ (BMNH). **PANAMA:** **Barro Colorado Island:** Canal Zone, 19 Oct 1971, D. Engleman, 1 ♀ (00165989) (AMNH). **Canal Zone:** Barro Colorado Island, 9.16667°N, 79.85°W, 08 Aug 1972, D. Engleman, light trap, 1 ♂ (00165958) (AMNH). Fort Amador, Feb 1964, Ch. Keenan, 1 ♀ (00161467) (USNM). Playa Venado, 30 Jul 1975 - 31 Jul 1975, E. M. ♀ J. L. Fisher, 1 ♂ (00161497) (USNM). **Cocle:** Agua Dulce, 07 Aug 1951, Blanton, 1 ♀ (00161581) (USNM). El Chiru, 10 Nov 1952, F. S. Blanton, 1 ♂ (00161593) (USNM). **Herrera:** Port Chitre, 24 Oct 1952, F. S. Blanton, 1 ♂ (00161719), 1 ♀ (00161720) (USNM). **PERU:** **Lima:** Barranca, 01 Jan 1900, Sauer, 1 ♂ (00161507) (USNM); 16 Sep 1940, collector unknown, 2 ♀♀ (00161608, 00161612) (USNM). **PHILIPPINES:** **Bulacan:** Malolos, 29 May 1972, A. D. Pawar, 1 ♀ (00166922) (CNC). **Laguna:** Los Banos, International Rice Research Institute, 10 Jul 1972, A. D. Pawar, *Oryza* sp. (Poaceae), 1 ♂ (00112064) (CNC). **Mountain:** Sagada, 09 Apr 1972, A. D. Pawar, 1 ♀ (00166923) (CNC). **PUERTO RICO:** **Arecibo:** Arecibo, 30 Jul 1914 - 01 Aug 1914, collector unknown, 1 ♀ (00165914) (AMNH). **Caguas:** Caguas, May 1965, Ricardo Jorge, 2 ♂♂ (00161528, 00161712), 3 ♀♀ (00161529, 00161713 - 00161714) (USNM). **Cayey:** Cayey, Sep 1960 - Nov 1960, M. M. Beauchamp, 1 ♂ (00161718), 1 nymph (00161717) (USNM); Sep 1960, M. Santiago, 2 ♂♂ (00161725, 00161726) (USNM). **Comerio:** Comerio, 05 Jun 1961, J. Maldonado C., 1 ♂ (00161727) (USNM). **Guanica:** State Forest, 30 Jun 1955, J. A. Ramos, J. Maldonado, 1 nymph (00161461) (USNM). **Guayanilla:** Guayanilla, Sep 1960 - Nov 1960, E. Murphy, 2 ♀♀ (00161715, 00161716) (USNM). **Lajas:** Lajas, 18.0519°N, 67.0597°W, 47 m, 01 May 1954 - 20 May 1954, J. Maldonado Capriles, 1 ♂ (00161592) (USNM); Sep 1960, J. Maldonado C., 1 ♂ (00161724) (USNM). **Mayaguez:** Guanajibo, 03 Aug 1935, H. L. Dozier, 1 ♀ (00161527) (USNM). Mayaguez, Mar 1960, J. Maldonado C., 1 ♂ (00161723) (USNM); Jul 1975, J. Maldonado C., 2 ♀♀ (00161451, 00161452) (USNM). **San Juan:** Rio Piedras, 18.3994°N, 66.0503°W, 29 m, 01 Jan 1972, G. F. and S. Hevel, 1 ♂ (00161590) (USNM). San Juan, 18.46633°N, 66.10573°W, 18 Feb 1964, L.T. Sanders, 1 ♀ (00161545) (USNM). Manati, 18.429°N, 66.492°W, 53 m, 27 Jun 1915 - 29 Jun 1915, collector unknown, 1 ♂ (00095358) (AMNH). **REUNION:** Savannah, 17 Jan 1990, B. Vercambre, 1 ♀ (00161553) (USNM); 09 Feb 1990, B. Vercambre, 1 ♂ (00161551), 1 ♀ (00161552) (USNM). **RODRIGUES ISLAND**: Aug-Nov 1918, H. J. Snell and H. P. Thomasset, 1 ♂, 1 ♀ (BMNH). **SAINT LUCIA:** Castries, 0-210 meters, Aug 1976, N. L. H. Krauss, 1 ♂ (00165986) (AMNH). **ST. HELENA**: Diana’s Peak, 27 June 1959, . R. Wallace, swept from grass, 1 ♀. Picquet Post. 27 Feb 1936, H. F. D. Barlett, 1 ♂ (BMNH). **SAUDI ARABIA:** **Ash Sharqiyah:** Al Hasa, Oct 1977, R. Linnavuori, 3 ♂♂ (00161697 - 00161699), 1 ♀ (00161700) (USNM). Al Hasa, 10 Nov 1917, R. Linnavuori, 4 ♂♂ (00165919 - 00165922), 2 ♀♀ (00165923, 00165924) (AMNH); Oct 1977, R. Linnavuori, 1 ♂ (00165925), 3 ♀♀ (`00165926 - 00165928) (AMNH); 05 Dec 1977, R. Linnavuori, 1 ♂ (00165931), 2 ♀♀ (00165929, 00165930) (AMNH); 02 Oct 1978, R. Linnavuori, 4 ♂♂ (00165915 - 00165918) (AMNH). El Riyadh, 24.63333°N, 46.71666°E, 31 Mar 1959 - 05 Apr 1959, E. W. Diehl, 1 ♀ (00235174) (ZISP). **SENEGAL:** **Dakar:** Dakar, 25 Oct 1943, J. Phillips, 2 ♂♂ (00161777, 00161778), 1♀ (00161779) (USNM). **SIERRA LEON:** Njala, 21-23 Nov. 1981, L. H. Rolston, 2 ♂♂, 3 ♀♀ (USNM). **SOMALIA:** Sar Uanle, 28 May 1973, S.B.S., 1 ♂ (00165999) (AMNH); 08 Jun 1973, S.B.S., 1 ♂ (00166022), 1 ♀ (00166040) (AMNH); 11 Jun 1973, S.B.S., 1 ♀ (00166025) (AMNH); 11 Jun 1973, R. Linnavuori, 1 ♀ (00166041) (AMNH). **SOUTH AFRICA:** **Cape Province:** Rondvlei near Knysna, 08 Feb 1968, T. Schuh, J. A. & S. Slater, M. Sweet, 1 ♂ (00165906) (AMNH). Wilderness, 12 Mar 1968 - 13 Mar 1968, P. J. Spangler, 1 ♂ (00161771), 2 ♀♀ (00161769, 00161770) (USNM). **Gauteng:** Lyttelton, 25.833°S, 28.216°E, 1492 m, 12 Jan 1968, J. A. & S. Slater, 1 ♀ (00165912) (AMNH); 29 Feb 1968, J. and S. Slater, 4 ♂♂ (00165902 - 00165905), 1 ♀ (00095359) (AMNH). **KwaZulu-Natal**: Port-Shepstone, 5.97, 1926, 1 ♀ (BMNH). **Northern Cape:** McDougall Bay, Port Nolloth, 29.28796°S, 16.87945°E, 2 m, 06 Sep 2004, Schuh, Schwartz, Henry, Wyniger, Forero, *Sporobolus virginicus* (L.) Kunth (Poaceae), det. J Manning VOUCHER-NYBG, 17 ♂♂ (00161735 - 00161751), 19 ♀♀ (00161752 - 00161765, 00161772 - 00161776) (USNM). **SPAIN**: **Canary Islands**: Grand Canary Island, Maspalomas, 14 Aug 1966, K. M. Guichad & P. H. Ward, 9 ♂♂, 6 ♀♀ (BMNH). **SUDAN:** **Bahr El-Jabal:** Equatoria, Juba, 27 Feb 1963 - 02 Mar 1963, R. Linnavuori, 5 ♂♂ (00166002 - 00166005, 00166013), 1 ♀ (00166042) (AMNH). Equatoria, Juba-Nimule, 10 Mar 1963 - 11 Mar 1963, R. Linnavuori, 1 ♂ (00166016) (AMNH). Equatoria, Lalyo-Juba, 26 Feb 1963 - 27 Feb 1963, R. Linnavuori, 1 ♀ (00166049) (AMNH). Equatoria, Loka Forest, 08 Apr 1963 - 10 Apr 1963, R. Linnavuori, 1 ♀ (00166050) (AMNH). Equatoria, Nimule, 11 Mar 1963 - 13 Mar 1963, R. Linnavuori, 1 ♂ (00166015) (AMNH). Central Equatoria, Juba, 27 Feb-2 Mar 1963, R. Linnavuori, 2 ♂♂, 2 ♀♀ (BMNH). **Blue Nile:** Singa-Damazin, 15 Nov 1962 - 17 Nov 1962, R. Linnavuori, 9 ♂♂ (00165975 - 00165983), 5 ♀♀ (00166030 - 00166034) (AMNH). Umm Banein, 14 Nov 1962, R. Linnavuori, 5 ♂♂ (00166007 - 00166011), 1 ♀ (00166035) (AMNH). Wad Madani [Medani], 14.4°N, 33.5°E, 11 Nov 1962 - 12 Nov 1962, R. Linnavuori, 1 ♂ (00166012), 2 ♀♀ (00166044, 00166045) (AMNH). Wad es Zaki, 10 May 1963, R. Linnavuori, 1 ♂ (00166014) (AMNH). **Kassala:** Kassala, 30 Nov 1962, R. Linnavuori, 1 ♀ (00166043) (AMNH). **Khartoum:** Khartoum, 13 Aug 1966, Stys, 1 ♂ (00161569), 1 ♀ (00161570) (USNM). **Nile:** Ed Damer, 05 Jul 1961 - 10 Jul 1961, R. Linnavuori, 2 ♂♂ (00166019, 00166020), 1 ♀ (00166047) (AMNH). Shendi, 02 Nov 1962 - 05 Nov 1962, R. Linnavuori, 1 ♀ (00166048) (AMNH). **Southern Kordofan:** Lake Keilak, 08 Feb 1963 - 11 Feb 1963, R. Linnavuori, 1 ♂ (00166018) (AMNH). Upper Nile, Malakal, 05 Jan 1963 - 20 Jan 1963, R. Linnavuori, 2 ♂♂ (00165984, 00166006) (AMNH). **Upper Nile:** Malakal, 05 Jan 1963 - 20 Jan 1963, R. Linnavuori, 3 ♀♀ (00166026 - 00166028) (AMNH). **Warab:** Bahr el Ghazal, 19 Feb 1963, R. Linnavuori, 1 ♂ (00166021) (AMNH). **White Nile:** Blue Nile, Kosti, 22 Jan 1963, R. Linnavuori, 2 ♂♂ (00166000, 00166001) (AMNH). Kordofan, Kadugli, 02 Feb 1963 - 14 Feb 1963, R. Linnavuori, 1 ♂ (00166017), 1 ♀ (00166029) (AMNH). APO 625, AMM #A857, 30 Oct 1943, collector unknown, 1 ♂ (00161784) (USNM). **TAIWAN:** **Nantou:** ***Ren-ai Township Co*.:** Huei-Sun For. Rec. Area, 5km NE of Meiyuan, 24.0667°N, 120.9833°E, 733 m, 10 Jul 1992 - 11 Jul 1992, T. J. Henry and A. G Wheeler, Jr., 1 ♀ (00161420) (USNM). **TANZANIA:** Ukiriguru, 18 May 1961, I. A. D. Robertson, ex light trap, 1 ♂, 5 ♀♀ (AMNH). **TRINIDAD AND TOBAGO:** **Arima:** Curepa, 11 Aug 1975, F. D. Bennett, 1 ♀ (00165988) (AMNH). **Port-of-Spain:** Port-of-Spain, 10.65°N, 61.5167°W, 09 Sep 1969, H.A. Wright, 1 ♀ (00161494) (USNM). **UNITED STATES:** **Florida:** ***Alachua Co*.:** Gainesville, 29.63527°N, 82.37111°W, 24 m, 29 Dec 1964, F.W. Mead, *Medicago sativa* (Fabaceae), 3 ♂♂ (00161670 - 00161672) (USNM); 25 Oct 1982, R. Nguyen, *Saccharum officinarum* (Poaceae), 1 ♀ (00161666) (USNM). ***Brevard Co*.:** Orsino, 13 Aug 1951, collector unknown, 1 ♂♂ (00161594) (USNM). ***Broward Co*.:** Deerfield Beach, 26 Jul 1948, R. H. Beamer, 1 ♀ (00165991) (AMNH). W. Park, 29.66806°N, 82.40194°W, 18 Aug 1939, collector unknown, 2 ♀♀ (00161400, 00161401) (USNM). ***Hendry Co*.:** Locality unknown, 31 Aug 1982, D. G. Hall, 1 ♀ (00161421) (USNM). ***Highlands Co*.:** Lake Placid, 27.29278°N, 81.36306°W, 13 Jul 1948, R. H. Beamer, 1 ♀ (00165992) (AMNH), 1 ♂ (00161673) (USNM). Sebring, 27.49555°N, 81.44083°W, 41 m, 24 Jul 1950 - 31 Jul 1950, C. T. Parsons, 2 ♀♀ (00165966, 00165967) (AMNH); 06 Aug 1950 - 12 Aug 1950, C. T. Parsons, 9 ♂♂ (00165960 - 00165965, 00165971 - 00165973), 1 ♀ (00165974) (AMNH); 10 Aug 1950 - 30 Aug 1950, C. T. Parsons, 2 ♀♀ (00165969, 00165970) (AMNH); 01 Sep 1950 - 16 Sep 1950, C. T. Parsons, 1 ♀ (00165968) (AMNH). ***Indian River Co*.:** Fellsmere, 04 Sep 1937, H.A.J., 1 ♀ (00161664) (USNM). Sebastian, 27.81642°N, 80.47061°W, Apr 1700, G. Nelson, 1 ♂ (00165985) (AMNH). ***Jefferson Co*.:** Monticello, 30.545°N, 83.87°W, 08 Oct 1965, W.H. Whitcomb, 1 ♀ (00161665) (USNM). ***Miami-Dade Co*.:** Airport Fumigation Site, 25.79966°N, 80.30733°W, 02 Jun 2008, T. Dobbs, light trap, 1 ♀ (00161412) (USNM). Biscayne Bay, 25.5747°N, 80.3112°W, 1700, Mrs. A.T. Slosson, 2 ♂♂ (00165907, 00165908) (AMNH), 1 nymph (00161404) (USNM); 01 Jan 1900, collector unknown, 2 ♀♀ (00165994, 00165995) (AMNH), 1 ♂ (00161397) (USNM). Coconut Grove, Mar 1900, G. Fairchild, 1 ♀ (00165993) (AMNH). Everglades National Park, 25.3125°N, 80.9375°W, 19 Jul 1973, C. W. O’Brien, 1 ♂ (00165959) (AMNH). Homestead, 25.46833°N, 80.47778°W, 26 Aug 1968, R. M. Baranowski, 1 ♂ (00161417) (USNM); 08 Dec 1974, J. A. Slater, 1 ♀ (00165990) (AMNH). Miami, 25.77389°N, 80.19389°W, 1 m, 01 May 2005, APHIS port inspector, 2 ♀♀ (00161498, 00161499) (USNM); 20 Dec 2009, APHIS port inspector, 1 ♂ (00161501) (USNM). ***Monroe Co*.:** Big Pine Key, Watsons Hammock, 24.68643°N, 81.36715°W, 14 Apr 1981, T. J. Henry and A. G. Wheeler, Jr., 3 ♀♀ (00161409 - 00161411) (USNM). ***Orange Co*.:** Orlando, 28.53805°N, 81.37944°W, 34 m, 02 Jul 1918, Geo. G. Ainslie, rye, 1 ♀ (00161399) (USNM). Winter Park, 28.59972°N, 81.33944°W, 16 Aug 1944, H. T. Fernald, 1 ♂ (00165910) (AMNH). ***Osceola Co*.:** CR 532, 1 km E. of CR 545, 3.5 km NNW of Loughman, 05 Apr 2003, A. G. Wheeler, Jr., 1 ♀ (00161413) (USNM). ***Palm Beach Co*.:** 2 miles south of co. line on Rt. 27, 20 Apr 1981, T. J. Henry, 1 ♂ (00161405) (USNM). Belle Glade, 26.68417°N, 80.66778°W, 10 Jul 1963, Tullosand Brandt, 1 ♀ (00161674) (USNM). Canal Point, 07 Feb 1983, O. Sosa Jr., 2 ♀♀ (00161418, 00161419) (USNM). Lake Worth, 26.58°N, 80.04°W, 01 Jan 1750, Mrs. A.T. Slosson, 1 ♀ (00165996) (AMNH); 01 Jan 1900, collector unknown, 1 nymph (00161398) (USNM). Palm Beach, 26.70528°N, 80.03667°W, 1 m, 27 Jul 1948, R. H. Beamer, 1 ♀ (00161500) (USNM); 19 Nov 1982, D. G. Hall, 3 ♂♂ (00161667 - 00161669) (USNM); 24 Nov 1982 - 26 Nov 1982, D. G. Hall, sugarcane, 1 ♂ (00161406), 2 ♀♀ (00161407, 00161408) (USNM). ***Pinellas Co*.:** Saint Petersburg, 27.7709°N, 82.6793°W, 12 Aug 1910, J. C. Bradley, 1 ♂ (00165987) (AMNH). ***Polk Co*.:** Lakeland, 28.0392°N, 81.95°W, 02 Oct 1948, R. F. Hussey, 1 ♂ (00165909) (AMNH); 14 Nov 1948, R. F. Hussey, 3 ♂ (00161554 - 00161556) (USNM); 24 Sep 1952, R. F. Hussey, 1 ♀ (00165911) (AMNH). **North Carolina:** ***Camden Co*.:** North River, 17 Oct 1959, L. Davis, 1 ♂ (00161414), 2 ♀♀ (00161415, 00161416) (USNM). **Virginia:** ***Louisa Co*.:** 4 mi. S. of Cuckoo, 01 Jun 1985, J. Kloke & D.R. Smith, 1 ♀ (00161828) (USNM). **VENEZUELA:** **Aragua:** ***Maracay Co*.:** Maracay, 10 Jul 1968, J. Maldonado C., 7 ♂♂ (00161557 - 00161563), 5 ♀♀ (00161564 - 00161568) (USNM). Maracay, 10.24694°N, 67.59583°W, 548 m, 10 Jul 1968, J. Maldonado C., 11 ♂♂ (00161422 - 00161432), 6 ♀♀ (00161434 - 00161439) (USNM). **Carabobo:** Mariara, Las Vueltas, 10.2983°N, 67.7161°W, 450 m, Aug 1970, L. J. Joly, 1 ♂ (00161460) (USNM). Saman Mocho, 10.1206°N, 67.8914°W, 595 m, Aug 1970, L. J. Joly, 1 ♂ (00161440), 5 ♀♀ (00161441 - 00161445) (USNM). Saman Mocho, 10.1206°N, 67.8914°W, 450 m, Aug 1970, L. J. Joly, 1 ♂ (00161453), 7 ♀♀ (00161454 - 00161459, 00161578) (USNM). Tocuyo, Aug 1970, L. J. Joly, 2 ♂♂ (00161447, 00161448), 1 ♀ (00161446) (USNM). **VIRGIN ISLANDS (U.S.):** **St. Thomas:** 3 miles from Charlotte Amalie, 31 May 1917, Harold Morrison, 1 ♂ (00161512) (USNM). Charlotte Amalie, 18.3439°N, 64.9311°W, 02 Jun 1917, Harold Morrison, 2 ♀♀ (00161517, 00161579), 1 nymph (00161518), 1 ♂ (00161403) (USNM). **YEMEN:** **Abyan:** Al Kowd, 13.08333°N, 45.36666°E, Jan 2000, van Harten Awad, 4 ♂♂ (00161691 - 00161694), 2 ♀ (00161695, 00161696) (USNM). **Ta’izz:** Ta’izz, Aug 1999 - Nov 1999, van Harten Awad, 7 ♂♂ (00161679 - 00161685), 5 ♀♀ (00161686 - 00161690) (USNM). Ta’izz, Jan., Jul., Aug., Sept., & Nov. 1999, van Harten & Awad, 30 ♂♂, 18 ♀♀ (NMW). Ta’izz, May, June, Aug., & Sept. 2000, van Hartenn & Al Yarimi, 8 ♂♂, 12 ♀♀ (NMW). Ta’izz, Abkhawkhah, 4 May 1992, R. Linnavuori, 1 ♂ (NMW). Abyan, A Kowd, Jul., Sept., & Dec. 1999, van Harten, Al Harun, & Sallam, 8 ♂♂, 10 ♀♀. Abyan, A Kowd, 4 May & 8-12 July 2001, van Harten & Asi Harun, 5 ♂♂, 1 ♀ (NMW). Abyan, A Kowd, 7 Sept. 2002, van Harten & Alö Harun, 3 ♀♀ (NMW). Abyan, A Kowd, Sept. 2003, van Harten & Al Haurn, 1 ♂. A Kowd, Jan., Feb., May, June, Jul., Oct. 2000, van Harten & Sallam, 22 ♂♂, 19 ♀♀ (NMW). Hudaydah, Zabid, 19-20 Mar. 1992, R. Linnavuori, 3 ♂♂, 1 ♀ (NMW). Sana’a Sana’a, Feb.-Mar. 1992, R. Linnavuori, 3 ♂♂, 1 ♀ (NMW). Sana’a, May & 1-10 Jul. 1999, van Harten, 3 ♂, 4 ♀♀ (NMW). Lahj: Lahi, 3-5 2002, van Harten & Sallam, 1 ♂, 3 ♀♀ (NMW). Al Kadan, Oct. 2001, van Harten & Abdul-Haq, 8 ♂♂, 2 ♀♀ (NMW).

### Tytthus piceus

(Osborn & Drake) comb. n.

http://species-id.net/wiki/Tytthus_piceus

Figs 35–37 71–78 167–170

Isoproba picea Osborn and Drake 1915: 533 (orig. descrip.); Carvalho 1952: 72 (as type); Carvalho 1958(2): 201 (cat.); Cassis 1984: 165 (subfam. note); Schuh 1995: 498 (cat.).
Tytthus hondurensis Carvalho 1984: 203 (orig. descrip.); Schuh 1995: 248 (cat.). **syn. n.**

#### Diagnosis.

This species, known from macropterous males and females (Figs 35, 36) and brachypterous females (Fig. 37), is distinguished by the bulbous black head, the black pronotum and scutellum having a distinct glaucous sheen, the raised finely punctate pronotal calli, the pale translucent-brown hemelytra, and the pale yellow to white first antennal segment with a narrow black ring at the base.

*Tytthus piceus* keys out with *Tytthus pallidus*, n. sp but can be distinguished by the bulbous head, especially in males, the shorter antennal segment I that is only subequal to the interocular width, and the distinct calli that are covered in a glaucous sheen.

#### Description.

*Male* (n = 10; holotype in parentheses) (Figs 35, 71, 72): Length to apex of hemelytron 2.35–2.50 mm (2.70 mm), length to base of cuneus 1.70–1.75 mm (1.89 mm), width across hemelytra 0.62–0.66 mm (wings folded). *Head*: Length 0.34–0.37 mm (0.35 mm), width across eyes 0.51–0.54 mm (0.50 mm), interocular width 0.24–0.26 mm (0.26 mm). *Labium*: Length 1.07–1.14 mm (imbedded in glue). *Antenna*: Segment I, length 0.26–0.27 mm (antennae missing); II, 0.98–1.02 mm; III, 0.50–0.56 mm; IV, 0.40–0.50 mm. *Pronotum*: Length 0.32–0.34 mm (0.32 mm), basal width 0.59–0.61 mm (0.59 mm).

*Coloration*: *Head* (Figs 73–75): Shiny black; eyes black. *Labium*: Segment I fuscous to black, apex paler yellowish brown, tinged with red or reddish brown in some specimens; segments II–IV pale yellowish brown, apex of segment IV fuscous. *Antenna*: Segment I pale yellowish brown to white, with a narrow black ring at base; segments II–IV uniformly black. *Pronotum, mesoscutum, and scutellum*: Shiny black, with a distinct glaucous sheen. *Hemelytron*: Uniformly smoky brown, slightly darker brown on clavus. *Ostiolar evaporative area* (Fig. 76): Dark reddish brown to fuscous. *Ventral surface*: Thorax dark brown or fuscous; abdomen in males dark brown, sometimes paler ventrally, genital capsule fuscous to black; abdomen in females yellowish green to pale brown, with broad lateral margins and ovipositor fuscous to black. *Legs*: Uniformly yellowish brown, inner face of pro- and mesofemora and outer face of metafemur usually with a narrow reddish line; claw (Fig. 78).

*Structure, texture, and vestiture*: *Head*: Impunctate, round or bulbous in both sexes, slightly wider than long; with scattered, long, erect setae on frons and vertex and a few short, erect setae on eyes. *Labium*: Extending to abdominal segment II or III. *Pronotum*: Trapeziform, narrowest anteriorly, lateral margins weakly concave, flaring to humeral angles; impunctate, except for a few scattered punctures on distinctly swollen calli. *Mesoscutum*: Broadly exposed. *Scutellum*: Equilateral, weakly convex, rising just above level of hemelytra. *Hemelytron*: Translucent, subparallel; in macropterous males and females (Figs 35, 36) cuneus longer than wide at base, membrane fully developed, extending well beyond apex of abdomen; in brachypterous females (Fig. 37) lateral margins slightly more rounded than macropters, cuneus reduced to about as wide as long, membrane greatly reduced, extending only to the sixth or seventh abdominal tergite, exposing apex of abdomen.

*Male genitalia*: *Left paramere* (Fig. 167): Mitt-shaped; right arm long, wide, apically blunt; left arm shorter, apically pointed. *Right paramere* (Fig. 168): Elongate oval. *Endosoma* (Fig. 169): Strongly C-shaped, apically blunt. *Phallotheca* (Fig. 170): Relatively slender, apically acute.

*Macropterous female* (n = 10) (Fig. 36): Length to apex of hemelytron 2.60–2.85 mm, length to base of cuneus 1.85–2.05 mm, width across hemelytra 0.70–0.83 mm. *Head*: Length 0.35–0.40 mm, width across eyes 0.54–0.58 mm, interocular width 0.27–0.29 mm. *Labium*: Length 1.18–1.22 mm, extending to near base of ovipositor. *Antenna*: Segment I, length 0.26 mm; II, 0.85–0.93 mm; III, 0.51–0.53 mm; IV, 0.43–0.45 mm. *Pronotum*: Length 0.34–0.37 mm, basal width 0.69–0.70 mm.

*Brachypterous female* (n = 3) (Fig. 37): Length to apex of hemelytron 1.80–1.90 mm; length to base of cuneus 1.55–1.60 mm; length to apex of abdomen 2.20–2.40 mm; width across hemelytra 0.64–0.70 mm. *Head*: Length 0.35–0.40 mm, width across eyes 0.54–0.58 mm, interocular width 0.29 mm. *Labium*: Length 1.10–1.14 mm, extending to abdominal segment II. *Antenna*: Segment I, length 0.26–0.29 mm; II, 0.82–9.94 mm; III, 0.51–.56 mm; IV, 0.43–0.45 mm. *Pronotum*: Length 0.29–0.34 mm, basal width 0.51–0.59 mm.

#### Host.

Adults and nymphs have been taken in large numbers on switch grass, *Panicum virgatum* L. cv ‘Alamo’ [Poaceae], infested with Delphacidae (A. G. Wheeler, pers. comm.).

#### Distribution.

This species was described and known previously only from Guatemala (as *Isoproba picea*) and Honduras (as *Tytthus hondurensis*). Colombia, Costa Rica, Mexico, Panama, and the United States (Florida, Maryland, and South Carolina) represent new country records and considerable range extensions.

#### Discussion.

Specimens of *Tytthus piceus* from Colombia, Costa Rica, Honduras, Mexico, and Panama represent an expected distribution for a Central American species, but the recent detection of it in Florida, Maryland, and South Carolina in the United States is somewhat of a mystery. The eastern United States is relatively well collected, so it seems unlikely that early workers overlooked this unusual bug, given that other obscure species of the genus with similar habits, such as *Tytthus vagus* (Knight) and the tiny *Tytthus alboornatus* (Knight), have been discovered. The most logical explanation is that *Isoproba piceus* has been inadvertently introduced relatively recently and moved around on ornamental grasses, such the one A. G. Wheeler found serving as a host in the South Carolina Botanical Gardens.

I have examined the holotypes of *Tytthus hondurensis* and *Tytthus piceus* and find them conspecific.

#### Type material examined.

**Holotype** ♂ (of *Isoproba picea*) (00162336) (OSU): **GUATEMALA:** Pt. Barios**:** 3/3/05 [J. S. Hine coll., as per Osborn and Drake, 1915], Herbert Osborn collection. **Holotype** ♂ (of *Tytthus hondurensis*) (00162205) (USNM): **HONDURAS:** **Atlantida:** Lancetilla, Aug 1701, Stadelmann.

#### Other specimens examined.

**COLOMBIA:** **Cundinamarca:** Sasaima, 4.96638°N, 76.4375°W, 1221 m, 28 Aug 1965, J. A. Ramos, 1 ♀ (00161785) (USNM). **COSTA RICA:** **Cartago:** Pejibaye, 24 Mar 1987 - 25 Mar 1987, W. E. Steiner, 1 ♀ (00161786) (USNM). **Puntarenas:** El Palmar, Jan 1962, J. O. Harrison, 2 ♂♂ (00161449 - 00161450) (USNM). **MEXICO**: ***San Luis Potosi*:** Huichihayan, 25 Sept. 1938, L. J. Lipovsky, 1 ♂ (UK).

**PANAMA**: Cerro Jefe, 12 Mar. 1969, R. L. Fischer, elev. 2000 ft., 1 ♀ (00166062) (AMNH). **UNITED STATES:** **Florida:** ***Miami-Dade Co*.:** Coral Gables, Matheson Hammock, 10 May 1997, Vince Golia, sweeping, 1 ♀ (00161829) (USNM). **Maryland:** ***Howard Co*.:** Howard Co. field, 18 Oct 1961, collector unknown, red clover (Fabaceae), 1 ♂ (00161827) (USNM). **South Carolina:** ***Pickens Co*.:** South Carolina Botanical Garden, Clemson, 28 Aug 2004, A. G. Wheeler, Jr., *Panicum virgatum*, 1 ♀ (00161813) (Poaceae), 5 ♂♂ (00161814 - 00161818), 7 ♀♀ (00161819 - 00161825), 1 nymph (00161826) (USNM); 31 Jul 2005, A. G. Wheeler, Jr., *Panicum virgatum* (Poaceae), 11 ♂♂ (00161787 - 00161797), 15 ♀♀ (00161798 - 00161812) (USNM).

### Tytthus pubescens

(Knight)

http://species-id.net/wiki/Tytthus_pubescens

Figs 38–40 79–86 171–174

Capsus geminus Flor 1860: 464 (orig. descrip.). Preoccupied by *Capsus geminus* Say, 1832 as noted by Henry and Wheeler 1988: 457.
Cyrtorhinus geminus : Reuter 1883: 382 (descrip.); Butler 1923: 480 (biol.); Wagner 1952: 129 (descrip., key).
Chlamydatus (Cyrtorhinus) geminus : Reuter 1875b: 126 (descrip.).
Cyrtorhinus pubescens Knight 1931: 172 (orig. descrip.). Synonymized by Carvalho and Southwood 1955: 28; resurrected by Henry and Wheeler 1988: 457.
Tytthus geminus : Fieber 1864: 83 (comb.); Carvalho and Southwood 1955: 28 (comb., descrip., distr., key); Kiritshenko 1951: 175 (key); Carvalho 1952: 81 (list); Carvalho 1958: 157 (cat.); Kerzhner 1964: 754 (key); Kelton 1980: 303 (descrip.host, distr.).
Tytthus geminus form *flori*Stichel 1956: 271 (orig. descrip.).
Tytthus geminus form *pallidior*Stichel 1956: 271 (orig. descrip.).
Tytthus pubescens : Henry and Wheeler 1988: 457 (cat., revised status); Wheeler and Henry 1992: 140 (distr., host); Polhemus 1994: 133 (list); Schuh 1995: 249 (cat.); Scudder 1997: 270 (distr., hosts); Kerzhner and Jovifov 1999: 442 (cat.); Maw et al. 2000: 121 (list); Wachman et al. 2004 (note, biol.); Wheeler 2011: 209 (distr., hosts).

#### Diagnosis.

This species is distinguished by the fuscous to black head; brown to black pronotum, often entirely or with only the posterior angles or posterior half whitish to pale brown; the pale yellow to yellowish brown antennal segment I and black segments II–IV; the long, erect setae in both sexes on antennal segment II as long or longer than the diameter of the segment; the uniformly pale, translucent hemelytra; and pale brownish yellow legs. Males of this species are always macropterous (Fig. 38); both macropters (Fig. 39) and brachypters (Fig. 40) are known for females, but brachypters are most common**.**

This Holoarctic species is superficially similar to another Holarctic species, T*ytthus pygmaeus*, in general color and size. *Tytthus pubescens* is readily distinguished by the pale antennal segment I, the long erect and semierect setae on antennal segments I and II, and the often pale humeral pronotal angles.

#### Description.

*Macropterous male* (n = 10) (Figs 38, 79, 80): Length to apex of hemelytron 2.59–3.01 mm, length to base of cuneus 1.98–2.37 mm, width across hemelytra 0.93–0.96 mm. *Head*: Length 0.30–0.32 mm, width across eyes 0.64–0.66 mm, interocular width 0.30–0.32 mm. *Labium*: Length 1.01–1.07 mm. *Antenna*: Segment I length 0.35–0.38 mm, II 0.88–0.94 mm, III 0.62–0.66 mm, IV 0.56–0.58 mm. *Pronotum*: Length 0.35–0.40 mm, basal width 0.74–0.78 mm.

*Coloration*: *Head* (Figs 81–83): Fuscous to black, with a small, yellow, interocular spot near inner margin of each eye; eyes reddish brown. *Labium*: Segments I–III pale yellow; segment IV brown. *Antenna*: Segment I pale yellow; segment II–IV fuscous to black. *Pronotum*: Uniformly fuscous to black, some specimens brown at posterior angles, others pale brown on posterior half, sometimes with dark brown invading darker anterior half. *Mesoscutum and Scutellum*: Fuscous to black. *Hemelytra*: Pale translucent yellow to whitish. *Ostiolar evaporative area* (Fig. 84): Fuscous to black. *Ventral surface*: Thorax and abdomen uniformly fuscous to black. *Legs*: Coxae pale yellow, the bases brown; femora, tibiae, tarsi, and claws (Fig. 86) uniformly pale yellow.

*Structure, texture, and vestiture*: *Head*: Weakly shiny, impunctate; set with relatively long, erect and semierect setae on vertex and frons. *Labium*: Extending to apices of meso- or bases of metacoxae; segment I extending just past base of head to anterior margin of xyphyus before procoxae. *Antenna*: Segment I set with rather short, sparse, recumbent setae and two to four or more long, erect, subapical, bristlelike setae; segment II densely set with short, recumbent setae, intermixed with erect and semierect setae mostly subequal in length to diameter of segment. *Pronotum*: Nearly rectangular; anterior angles rounded; lateral margins straight, only slightly widening to posterior angles; posterior margin straight or only very slightly sinuate; set with relatively long, recumbent and semierect setae. *Mesoscutum*: Broadly exposed, even in brachypters; with a few scattered, semierect setae. *Scutellum*: weakly shiny, equilateral; set with relatively long, semierect setae. *Hemelytron*: Macropterous, subparallel; cuneus and membrane fully developed, extending beyond apex of abdomen; evenly set with recumbent setae.

**Figures 171–178.**
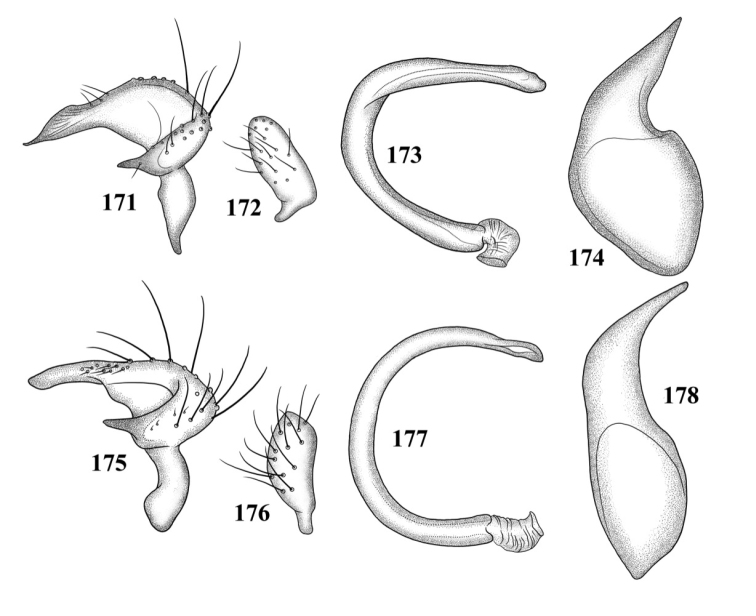
Male genitalia **171–174** *Tytthus pubescens* **171** left paramere **172** right paramere **173** endosoma **174** phallotheca **175–178** *Tytthus pygmaeus* **175** left paramere **176** right paramere **177** endosoma **178** phallotheca.

*Male genitalia* (Fig. 85): *Left paramere* (Fig. 171): Right arm broad, widened through middle, tapering to a fine point apically; left arm short, apically pointed. *Right paramere* (Fig. 172): Elongate oval. *Endosoma* (Fig. 173): Strongly C-shaped, apically blunt. *Phallotheca* (Fig. 174): Broad, apically acute.

*Macropterous female* (n = 10) (Fig. 39): Length to apex of hemelytron 2.66–3.10 mm, length to base of cuneus 1.98–2.24 mm, width across hemelytra 1.06–1.15 mm. *Head*: Length 0.30–0.32 mm, width across eyes 0.64–0.67 mm, interocular width 0.32–0.34 mm. *Labium*: Length 1.12–1.15 mm. *Antenna*: Segment I length 0.29–0.30 mm, II 0.69–0.72 mm, III 0.51–0.54 mm, IV 0.48–0.51 mm. *Pronotum*: Length 0.35–0.38 mm, basal width 0.82–0.93 mm.

Macropters have normally developed hemelytra that extend well beyond the apex of the abdomen as in males. Out of 63 females examined, only 10 are macropterous.

*Brachypterous female* (n = 10) (Fig. 40): Length to apex of abdomen 2.34–2.66 mm, length to base of cuneus 2.05–2.11 mm, width across hemelytra 0.94–1.15 mm. *Head*: Length 0.32–0.34 mm, width 0.67–0.69 mm, interocular width 0.32–0.34 mm. *Labium*: Length 1.10–1.17 mm. *Antenna*: Segment I length 0.27–0.32 mm, II 0.75–0.80 mm, III 0.50–0.54 mm, IV 0.45–0.53 mm. *Pronotum*: Length 0.40–0.42 mm, basal width 0.75–0.83 mm.

Brachypters are broadly rounded, with the cuneus sometimes slightly shortened and membrane greatly reduced and not attaining the apex of the abdomen.

#### Host.

In North America, this species has been collected on *Carex* sp. (Kelton 1980) and *Carex utriculata* Boott [Cyperaceae] (Wheeler 2011). It has been taken on rushes, sedges, and grasses in England where it is said to prey on leafhopper eggs (Butler 1923, Southwood and Leston 1959, Rothschild 1963, Wheeler and Henry 1992). Specimens from Arizona were beaten from clumps of deer grass, *Muhlenbergia rigens* (Benth.) Hitchc. [Poaceae].

#### Distribution.

This Holarctic species was previously known in North America from Alaska, Alberta, British Columbia, Colorado, Newfoundland, New Mexico, Quebec, Saskatchewan, and Yukon Territory (Henry and Wheeler 1988, Wheeler and Henry 1992, Polhemus 1994, Maw et al. 2000, Wheeler 2011). Arizona is a new state record.

In the Old World, it is known in Asia from Russia (East Siberia, Far East, and West Siberia) and in northern Europe from Austria, Byelorussia, Czech Republic, Denmark, Estonia, Finland, France, Great Britain, Germany, Hungary, Ireland, Latvia, Luxembourg, Moldavia, The Netherlands, Norway, Poland, Russia (Central European and Northern European territories), Slovakia, Sweden (Wheeler and Henry 1992, Kerzhner and Josifov 1999).

#### Type material examined.

Holotype ♀ (brachypterous) of *Tytthus pubescens* (USNM) **UNITED STATES:** ***Colorado*:** Wray, 4 Aug 1925, H. H. Knight (USNM).

#### Other specimens examined.

**CANADA:** **Alberta:** Jasper National Park, Banff-Jasper Highway, 26 Aug 1970 - 28 Aug 1970, L. A. Kelton, *Carex* sp. (Cyperaceae), 24 ♀♀ (00167011 - 00167034) (CNC). McMurray, 56.73333°N, 111.38333°W, 11 Jul 1953, W. J. Brown, 1 ♀ (00167052) (CNC). **British Columbia:** Atlin, 59.58333°N, 133.71666°W, 2199 m, 21 Jul 1955, B. A. Gibbard, 1 ♀ (00167051) (CNC); 14 Aug 1955, H. J. Huckel, 2 ♀♀ (00167037, 00167050) (CNC). Pink Mountain, 57.05°N, 122.86666°W, 17 Aug 1982, L. A. Kelton, 1 ♀ (00167141) (AMNH). Pouce Coupe, 55.7167°N, 120.1333°W, 18 Aug 1982, L. A. Kelton, *Beckmannia syzigachne* (Poaceae), 1 ♂ (00167036) (CNC). Terrace, 54.51241°N, 128.59637°W, 67 m, 27 Jul 1960, collector unknown, 1 ♀ (00166931) (CNC). **Ontario:** Fergus, 43.7°N, 80.36666°W, 24 Jul 1962, Kelton and Thorpe, 1 ♂ (00167055) (CNC). **Quebec:** Bonaventure Co., Escuminac, 48.11667°N, 66.48333°W, 2 m, 22 Aug 1983, Larochelle and Lariviere, 2 ♀♀ (00167046, 00167047) (CNC). Lac Mondor, Ste. Flore, 46.61666°N, 72.76666°W, 06 Jul 1951, E. G. Munroe, 1 ♂ (00167010) (CNC). **Saskatchewan:** Candle Lake, 53.74996°N, 105.25°W, 499 m, 19 Aug 1959, A. and J. Brooks, 1 ♂ (00167008) (CNC). Elbow, 51.11666°N, 106.6°W, 14 Jul 1960, A. R. Brooks, 1 ♂ (00167006) (CNC). **Yukon Territory:** 58 mi E of Dawson, Gravel Lake, 64.06666°N, 137.49935°W, 625 m, 12 Aug 1962, R. E. Leech, 1 ♀ (00167035) (CNC). Haines Junction, 60.75°N, 137.5°W, 28 Jul 1982, L. A. Kelton, 1 ♂ (00167005), 1 ♀ (00167004) (CNC). Koidern, 61.98333°N, 140.5°W, 24 Jul 1982, L. A. Kelton, 2 ♀♀ (00167038, 00167039) (CNC). Moose Creek, 18 Jul 1982, L. A. Kelton, 1 ♂ (00167009) (CNC). Rancheria, 60.08333°N, 130.6°W, 28 Jul 1982, L. A. Kelton, 1 ♂ (00167007) (CNC). Tagish, 60.3°N, 134.26666°W, 11 Aug 1983, L. A. Kelton, 6 ♀♀ (00167041 - 00167045, 00167053) (CNC). Takhini Hot Springs, 60.85°N, 135.51666°W, 732 m, 19 Aug 1962, R. E. Leech, *Betula* sp. (Betulaceae), 2 ♂♂ (00167000, 00167001), 2 ♀♀ (00167002, 00167003) (CNC). **FINLAND:** **Lansi-Suomen:** Pargas (Parainen), 60.3°N, 22.3°E, V. Jakovlev coll., 1 ♀ (00235128) (ZISP). Raisio, 60.4833°N, 22.1833°E, 28 m, R. Linnavuori, 1 ♀ (00161842) (USNM); Suomi V Raisio, 16 Sep 1947, R. Linnavuori, 2 ♀♀ (BMNH). **Lapland:** Pisavaara Nature Reserve, 03 Jul 1950, Hakan Lindberg, 1 ♂ (00161847) (USNM); 14 Jul 1950, Hakan Lindberg, 1 ♀ (00167054) (CNC). **Southern Finland:** ***Uusimaa Co*.:** Ekenas, 24 Jul 1936, Hakan Lindberg, 1 ♀ (00161843) (USNM). Helsinki, 19 Aug 1946, Hakan Lindberg, 2 ♀♀ (00167048, 00167049) (CNC). **Western Finland:** Pargas, 1900, Reuter, 2 ♂♂ (00161845, 00161846), 1 nymph (00161844) (USNM); Pargus J. Sahlberg, Saunders coll., Brit. Mus. 1910-357, 1 ♀ (BMNH). Kiumvesi, 4 Aug. 1951, R. Linnavuori, 1 ♂, 1 ♀ (NMW). **RUSSIAN FEDERATION:** **Kostroma Prov.:** Ugory, Manturovskiy Dist., 58.13333°N, 44.35°E, 09 Sep 1983, Veselova, *Carex gracilis* (Cyperaceae), 3 ♀♀ (00235130) (ZISP). **Leningrad Prov.:** St.-Petersburg [Petrograd], Novaya Derevnya, 59.98333°N, 30.26666°E, 15 Aug 1924, A. N. Kiritshenko, 2 ♂♂ (00235136), 3 ♀♀ (00235133 - 00235134, 00235136) (ZISP). **UNITED KINGDOM:** **England:** ***Locality unknown Co*.:** 1800, C. F. Baker, 1 ♂ (00161837) (USNM). **UNITED STATES:** **Alaska:** ***Anchorage Co*.:** 30 mi NE of Anchorage, 04 Aug 1948, R. I. Sailer, 1 ♂ (00161832), 2 ♀♀ (00161830, 00161831) (USNM). **Arizona:** ***Santa Cruz Co*.:** Audubon Research Ranch, Elgin, Finley Tank, 14 May 2004, A. G. Wheeler, Jr., *Muhlenbergia rigens* (Poaceae), 1 ♂ (00161835), 1 ♀ (00161833), 1 nymph (00161834) (USNM). **Colorado:** ***Garfield Co*.:** Grizzly Creek, 1896, C. F. Baker, 1 ♂ (00161838) (USNM). ***Larimer Co*.:** Chambers Lake, 01 Aug 1896, C. F. Baker, 1 ♀ (00161839) (USNM). Mountain Park Campground, Roosevelt National Forest, 40.6825°N, 105.4675°W, 2012 m, 06 Aug 1968, L. A. Kelton, 1 ♀ (00167040) (CNC). ***Yuma Co*.:** Wray, 40.07583°N, 102.22278°W, 1128 m, 04 Aug 1925, H. H. Knight, 1 ♂ (00162204) (USNM). ‘Colo’ 2074, 1900, P.R. Uhler Collection, 1 ♀ (00161840) (USNM). **Idaho:** ***Idaho Co*.:** 10.2 mi WSW of Lolo Pass, Powell Pasture, 46.46274°N, 114.89083°W, 1097 m, 22 Jul 1978, N. L. Herman, 1 ♂ (00166051) (AMNH). **New Mexico:** ***Sandoval Co*.:** Valles Caldera National Preserve, East Fork Jemez River, 35.8483°N, 106.49048°W, 20 Aug 2007, A. G. Wheeler, Jr., 1 ♀ (00161836) (USNM); 01 Aug 2008, A. G. Wheeler, Jr., *Carex utriculata* (Cyperaceae), 4 ♂♂ (00162194 - 00162197), 6 ♀♀ (00162188 - 00162193) (USNM). Valles Caldera National Preserve, Main Road at East Jemez River, 35.84826°N, 106.49051°W, 17 Aug 2009 - 18 Aug 2009, A. G. Wheeler, Jr., *Carex utriculata* (Cyperaceae), 4 ♂♂ (00162184 - 00162187) (USNM). Valles Caldera National Preserve, below historic ranch headquarters, 35.86363°N, 106.51681°W, 17 Aug 2009 - 18 Aug 2009, A. G. Wheeler, Jr., *Carex utriculata* (Cyperaceae), 7 ♂♂ (00162177 - 00162183), 13 ♀♀ (00162164 - 00162176) (USNM).

### Tytthus pygmaeus

(Zetterstedt)

http://species-id.net/wiki/Tytthus_pygmaeus

Figs 41–44 , 175–178

Capsus pygmaeus Zetterstedt 1838: 279 (orig. descrip.). Lectotype designated by Carvalho and Southwood 1955: 24.
Capsus pellucens Boheman 1852: 75 (orig. descrip.). Synonymized by Sahlberg 1868: 176.
Tytthus insignis Douglas and Scott 1866: 247 (orig. descrip.). Synonymized by Saunders 1876: 113.
Capsus (Cyrtorhinus) pygmaeus Thomson 1871: 437 (key).
Leptomerocoris insignis : Walker 1873: 131 (list).
Tytthus pygmaeus : Fieber 1864: 83 (descrip.); Carvalho and Southwood 1955: 23 (descrip., key); Carvalho 1958: 158 (cat.); Kerzhner 1964: 754 (key); Kelton 1980: 289 (distr., host); Kerzhner 1988: 840 (key); Henry and Wheeler 1988: 458 (cat.); Wheeler and Henry 1992: 141 (distr., host); Schuh 1995: 250 (cat.); Scudder 1997: 271 (distr., hosts); Kerzhner and Josifov 1999: 442 (cat.); Wachmann et al. 2004: 271 (note, biol., host, photo); Wheeler 2011: 210 (distr., hosts).
Chlamydatus pygmaeus Reuter 1875a: 31 (key); 1875b: 282 (descrip.).
Cyrtorhinus pygmaeus : Reuter 1883: 554 (descrip., key); Saunders 1892: 283 (descrip., hosts); Van Duzee 1917: 824 (cat.); Butler 1923: 480 (biol.); Kiritshenko 1951: 175 (key); Wagner 1952: 128 (descrip., key).
Tytthus pygmaeus form *flavomarginata*Stichel 1956: 272 (orig. descrip.). Synonymized by Schuh 1995: 249
Tytthus pygmaeus form *flavescens*Stichel 1956: 272 (orig. descrip.). Synonymized by Schuh 1995: 249.
Tytthus vagus : Polhemus 1994: 133 (list). See discussion under *Tytthus vagus*.

#### Diagnosis.

This highly variable species is distinguished by the black head, with relatively vague interocular spots; entirely pale (European material) to entirely fuscous to black (all North American material) pronotum, with intermediate color forms; very clear or translucent white hemelytra; the fuscous to black antennal segment I, with the apical one fourth pale yellow; antennal segments II–IV uniformly fuscous to black; and the uniformly pale yellow legs. All males and most females of this species are fully macropterous; only a few weakly brachypterous females, with the membrane extending to the apex of the abdomen, have been examined.

*Tytthus pygmaeus* is superfically similar to the Holarctic *Tytthus pubescens* in overall size, coloration, and distribution. It can be distinguished from *Tytthus pubescens* by the fuscous antennal segment I having only the apex pale, the short, recumbent setae on antennal segments I and II, and the variably fuscous to pale pronotum (but never fuscous with pale humeral angles as in *Tytthus pubescens*).

#### Description.

*Male* (n = 10) (Figs 41, 43): Length to apex of hemelytron 2.50–2.94 mm, length to base of cuneus 1.98–2.14 mm, width across hemelytra 0.94–0.98 mm. *Head*: Length 0.29–0.32 mm, width across eyes 0.69–0.75 mm, interocular width 0.30–0.32 mm. *Labium*: Length 1.07–1.12 mm. *Antenna*: Segment I length 0.30–0.32 mm, II 0.98–1.04 mm, III 0.61–0.62 mm, IV 0.54–0.58 mm. *Pronotum*: Length 0.35–0.40 mm, basal width 0.77–0.82 mm.

*Coloration*: *Head*: Shiny fuscous to black, with a relatively small, yellow, interocular spot near inner margin of each eye; eyes fuscous to dark reddish brown. *Labium*: Uniformly pale yellow, with apex of segment IV usually brown. *Antenna*: Segment I, fuscous to black, with only apical one fourth to one third pale yellow; segment II–IV black. *Pronotum*: Highly variable, ranging from uniformly pale yellow to entirely fuscous to black, with many intermediate forms, including all of disc dark and anterior half yellow and almost entirely dark with yellow across anterior margin and through middle of calli; some yellow specimens with only fuscous posterior angles. *Mesoscutum and Scutellum*: Uniformly fuscous to black. *Hemelytron*: Uniformly clear to very pale translucent white. *Ostiolar evaporative area*: Fuscous to black, even on palest specimens. *Ventral surface*: Thorax uniformly fuscous; abdomen fuscous along lateral margins and genital capsule, ventral area and sides pale yellow to whitish. *Legs*: Uniformly pale yellow.

*Structure, texture, and vestiture*: *Head*: Shiny, impunctate, much broader than long; set with numerous, long, erect and semierect setae on vertex and frons. *Labium*: Extending to apices of meso- or bases of metacoxae; segment I extending just beyond head to anterior margin of xyphus just before procoxae. *Antenna*: Segment I sparsely set with recumbent setae and two, long, erect, subapical, bristlelike setae; segment II, thickly set with short, recumbent and semierect setae. *Pronotum*: Trapeziform, anterior angles nearly weakly rounded; lateral margins straight, gradually widening to posterior angles; posterior margin nearly straight or only weakly sintuate. *Mesoscutum*: Broadly exposed. *Scutellum*: Equilateral; sparsely set with scattered relatively short, semierect setae. *Hemelytra*: Macropterous, cuneus and membrane fully developed, extending well beyond apex of abdomen; evenly set with recumbent setae.

*Male genitalia*: *Left paramere* (Fig. 175): Right arm long, broad, apically blunt; left arm short, apically pointed. *Right paramere* (Fig. 176): Elongate oval. *Endosoma* (Fig. 177): Strongly C-shaped, apically blunt. *Phallotheca* (Fig. 178): Slender, apically acute.

*Female* (n = 10) (Figs 42, 44): Length to apex of hemelytron 2.92–3.17 mm, length to base of cuneus 2.21–2.34 mm, width across hemelytra 1.20–1.31 mm. *Head*: Length 0.34–0.35 mm, width across eyes 0.74–0.75 mm, interocular width 0.34–0.35 mm. *Labium*: Length 1.12–1.20 mm. *Antenna*: Segment I length 0.27–0.29 mm, II 0.86–0.88 mm, III 0.61–0.62 mm, IV 0.54–0.58 mm. *Pronotum*: Length 0.40–0.42 mm, basal width 0.91–0.96 mm.

#### Hosts.

In North America, this species has been recorded from sedges, *Carex* spp. (Kelton 1980) and *Carex urtriculata* [Cyperaceae] (Wheeler 2011). In the Old World, it has been taken on European beachgrass, *Ammophila arenaria* (L.) Link [Poaceae], and on rushes, *Juncus* spp., where it feeds on the delphacid *Conomelus anceps* (Germar) (Scudder 1957, Wheeler and Henry 1982). Rothschild (1963) studied the biology and illustrated the fifth instar. Ehanno (1987) studied the life history in France and reported *Juncus effusus* L. (as *conglomeratus* L.) [Juncaceae] and *Typha* sp. [Typhaceae] as hosts.

#### Distribution.

This Holarctic species is known in Canada from Alberta, British Columbia, Labrador, Newfoundland, Ontario, Saskatchewan, and Yukon Territory and in the United States from New Mexico and Wyoming (Kelton 1980, Henry and Wheeler 1988, Wheeler and Henry 1992, Maw et al. 2000, Wheeler 2011). Colorado is a new state record.

In the Old World, it is known in Asia from Russia (East Siberia, Far East, and West Siberia) and in Europe from Austria, Byelorussia, Czech Republic, Denmark, Estonia, Finland, France, Germany, Hungary, Latvia, Luxembourg, Moldavia, The Netherlands, Norway, Poland, Russia (Central European and North European territories), Slovakia, Sweden, and the United Kingdom (Carvalho and Southwood 1955, Wheeler and Henry 1992, Kerzhner and Josifov 1999).

#### Specimens examined.

**CANADA:** **Alberta:** 25 mi N of Nordegg, 52.73804°N, 116.2332°W, 1298 m, 20 Jul 1987, S. A. Marshall, *Carex* sp. (Cyperaceae), 1 ♂ (00382161) (DEBU). Coal Valley, 53.08°N, 116.8°W, 31 Aug 1970, L. A. Kelton, 1 ♀ (00167065) (CNC). **Newfoundland and Labrador:** Hebron, 58.2°N, 62.63333°W, 19 Jul 1954, J. F. McAlpine, 2;m (00167056, 00167057), 6 ♀♀ (00167058 - 00167063) (CNC). **Ontario:** Elmira, 13 Jun 1958 - 19 Jun 1958, J. Juillet, 1 ♂ (00167067) (CNC). Elora, 43.68242°N, 80.43364°W, 384 m, 27 Jun 1977, K. Barber, 1 ♂ (00382166) (DEBU). Guelph, 43.55°N, 80.25°W, 323 m, 24 Jun 1977, P. R. Heels, 3 ♂♂ (00382162 - 00382164), 1 ♀ (00382165) (DEBU). Iroquois Falls, 48.77177°N, 80.66576°W, 238 m, 30 Jun 1987, J. R. Vockeroth, 2 ♂♂ (00166943, 00166992) (CNC). Marmora, 44.48333°N, 77.68333°W, 25 Jun 1952, J. R. Vockeroth, 1 ♂ (00166945) (CNC). Ottawa, 45.39079°N, 75.70324°W, 71 m, 15 Jul 1964, J. R. Vockeroth, 1 ♀ (00167066) (CNC). **Saskatchewan:** Christopher Lake, 53.56666°N, 105.83333°W, 11 Jun 1959, A. and J. Brooks, 2 ♀♀ (00166939, 00166940) (CNC); 13 Jul 1959, A. and J. Brooks, 3 ♀♀ (00166934 - 00166936) (CNC); 15 Jul 1959, A. and J. Brooks, 1 ♂ (00166929), 2 ♀♀ (00166937, 00166938), 1 nymph (00166928) (CNC). **Yukon Territory:** 14 mi E of Dawson, 64.01938°N, 139.10712°W, 396 m, 29 Jul 1962, R. E. Leech, 1 ♂ (00166944) (CNC). 58 mi E of Dawson, Gravel Lake, 64.06666°N, 137.49935°W, 625 m, 10 Aug 1962, R. E. Leech, 1 ♂ (00166941) (CNC); 12 Aug 1962, R. E. Leech, 1 ♀ (00166942) (CNC). Takhini Hot Springs, 60.85°N, 135.51666°W, 732 m, 19 Aug 1962, R. E. Leech, *Betula* sp. (Betulaceae), 1 ♀ (00166999) (CNC); 19 Aug 1962, P. J. Skitsko, 1 ♂ (00166989) (CNC). **FINLAND:** **Lansi-Suomen:** Raisio, 60.4833°N, 22.1833°E, 28 m, R. Linnavuori, 2 ♂♂ (00138748, 00138748) (AMNH). Locality unknown, Reuter, 1 ♀ (00235138) (ZISP). **Southern Finland:** Tvarminne, 12 Aug 1960, G.G.E. Scudder, 1 ♀ (00167068) (CNC). Ob Rovaniemi Posa, 29 Jul. 1950, H. Lindberg, 1 ♀ (NMW). **KYRGYZSTAN:** Upper course of Gava-Say [Gava] River, 41.16666°N, 72.86666°E, 10 Aug 1937, A. N. Kiritshenko, 1 ♂ (00235045) (ZISP). **RUSSIAN FEDERATION:** **Amur Prov.:** Klimoutsy, 40 km W of Svobodnyi, 51.4667°N, 127.5833°E, 242 m, 30 Jun 1959, I. M. Kerzhner, 1 ♂ (00235048) (ZISP). Simonovo, 75 km W Svobodnyi, 51.45°N, 126.96666°E, 19 Jul 1959, I. M. Kerzhner, 1 ♀ (00235144) (ZISP). **Chita Prov.:** Kharanor, 50.0833°N, 116.6667°E, 12 Jul 1963, I. M. Kerzhner, 10 ♂♂ (00235035 - 00235044), 6 ♀♀ (00235146 - 00235151) (ZISP). **Irkutsk Prov.:** Khargino [Kharga], SW Baikal, 52.31666°N, 105.76666°E, 19 Jul 1950, A. N. Kiritshenko, 3 ♀♀ (00235141 - 00235143) (ZISP). **Kamchatka Prov.:** Uzon volcano, 54.51666°N, 159.8°E, 11 Aug 1985, I. M. Kerzhner, 2 ♂♂ (00235049, 00235050), 3 ♀♀ (00235049, 00235050), 1 nymph (00235049) (ZISP). **Komi Rep.:** Vodnyy, 63.5°N, 53.4°E, 21 Aug 1964, collector unknown, 1 ♀ (00235165) (ZISP). **Kostroma Prov.:** Shilovo, ~25 km W of Manturovo, 58.33333°N, 44.33333°E, 12 Jul 1981, Veselova, 2 ♀♀ (00235166) (ZISP). **Leningrad Prov.:** Berezhok 20 km W Sosnovo, 60.55°N, 29.91666°E, 01 Aug 1961 - 15 Aug 1961, I. M. Kerzhner, 1 ♂ (00235034) (ZISP). Lebyazhye, 59.93333°N, 29.41666°E, 30 Jun 1898, Bianchi, 1 ♀ (00235139) (ZISP). Serezhino, 59.43°N, 28.32°E, 24 Aug 1895, Bianchi, 1 ♀ (00235140) (ZISP). **Sakhalin Prov.:** Novoaleksandrovsk, South Sakhalin, 47°N, 142.7°E, 07 Sep 1973, I. M. Kerzhner, 1 ♀ (00235164) (ZISP). **Yakutia Rep.:** Amga [Amginskaya Sloboda], 60.9°N, 132.01666°E, 12 Aug 1925, Bianchi, 1 ♀ (00235145) (ZISP). Balagannakh, 30 km ESE of Ust’-Nera, 64.498°N, 143.857°E, 04 Jul 1974, N.N. Vinokurov, 3 ♂♂ (00235046), 2 nymphs (00235131) (ZISP); 06 Jul 1974, Narchuk, 1 ♂ (00235047) (ZISP); 08 Jul 1974, Narchuk, 1 ♀ ( 00235160) (ZISP). Batagay on Yana river, NE Yakutia (80 km E Verkhoyansk), 67.65°N, 134.63333°E, 25 Jul 1974, N.N. Vinokurov, 4 ♀♀ (00235157, 00235158) (ZISP). Icing Bulus on Lena River 100 km upstream Yakutsk, Central Yakutia, 62.83333°N, 129.73333°E, 05 Jul 1996 - 07 Jul 1996, Watabe, 1 ♂ (00235051) (ZISP). Kolyma, Zyryanka, 65.73333°N, 150.91666°E, 05 Jul 1973, N.N. Vinokurov, 4 ♀♀ (00235152 - 00235155) (ZISP); 07 Jul 1973, N.N. Vinokurov, 1 ♀ (00235156) (ZISP). Mouth of Kharayuryakh River, NNW Artyk, 64.33333°N, 145.01666°E, 14 Jul 1974, Narchuk, 1 ♀ (00235159) (ZISP). Nr Olekminsk, 60.38333°N, 120.18333°E, 01 Aug 1974, N.N. Vinokurov, 9 ♀♀ (00235161 - 00235163) (ZISP). **UNITED KINGDOM:** **England:** ***Berkshire Co*.:** Burnham Beeches, 51.54932°N, 0.63311°W, 57 m, 17 Jul 1960, G. G. E. Scudder, 2 ♂♂ (00166990, 00166993), 1 ♀ (00166997) (CNC). ***East Sussex Co*.:** Camber, 05 Sep 1964, G.G.E. Scudder, 1 ♀ (00167064) (CNC). ***Oxfordshire Co*.:** Cothill, 51.69446°N, 1.33529°W, 82 m, 14 Jul 1960, G. G. E. Scudder, 1 ♀ (00166998) (CNC). Kennington, Berks., 51.71877°N, 1.24105°W, 58 m, 15 Jul 1960, G. G. E. Scudder, 7 ♂♂ (00166984, 00166986 - 00166988, 00166994 - 00166996), 1 nymph (00166985) (CNC). Wolvercote, 51.7854°N, 1.2902°W, 60 m, 14 Jul 1960, G. G. E. Scudder, 1 ♂ (00166930), 2 ♀♀ (00166926, 00166927) (CNC). ***Somerset Co*.:** Porlock, 51.2°N, 3.6667°W, 399 m, 11 Jul 1960, G. G. E. Scudder, 21 ♂♂ (00166947 - 00166953, 00166961 - 00166971, 00166981 - 00166983), 16 ♀♀ (00166954 - 00166960, 00166972 - 00166980) (CNC). ***Locality unknown*:** unknown, V. Jakovlev coll., 1 ♂ (00235032), 1 ♀ (00235137) (ZISP). **UNITED STATES:** **Colorado:** ***Garfield Co*.:** Grizzly Creek, 24 Jul 1896, C. F. Baker, 1 nymph (00161848) (USNM). **New Mexico:** ***Sandoval Co*.:** Valles Caldera National Preserve, East Fork Jemez River, 35.8483°N, 106.49048°W, 01 Aug 2008, A. G. Wheeler, Jr., *Carex utriculata* (Cyperaceae), 1 ♀ (00162160) (USNM). **Wyoming:** ***Laramie Co*.:** Laramie, 41.31°N, 105.59°W, 08 Jul 1947 - 09 Jul 1947, D. G. Denning, 1 ♂ (00166946) (CNC).

### Tytthus uniformis

Henry sp. n.

urn:lsid:zoobank.org:act:682B99D0-90E6-45BE-8565-BC96CDBAA1D9

http://species-id.net/wiki/Tytthus_uniformis

Figs 45–47 87 –100 179–182

#### Diagnosis.

This species is distinguished by the contrasting black head and antennal segments I and II and the uniformly pale orange-brown pronotum and legs, and the slightly more smoky orange-brown hemelytra. All males are macropterous and all females examined, except two macropters, are brachpyterous.

*Tytthus uniformis* is most similar in overall appearance to *Tytthus balli* and *Tytthus insperatus*. It can be distinguished from *Tytthus balli* by uniformly brownish-orange pronotum and hemelytra; *Tytthus balli* is usually infuscated on the posterior half of the pronotum, and the inner half of the clavus and apical third of the corium are dark brown. It is distinguished from *Tytthus insperatus* by the uniformly pale orange pronotum and legs; *Tytthus insperatus* has a dark brown pronotum, each femur has a narrow dorsal and lateral red stripe, and the hind tibiae are fuscous.

#### Description.

*Macropterous male* (n= 10, plus holotype in parentheses) (Figs 45, 87, 88): Length to apex of hemelytron 2.45–2.60 mm (2.25 mm); length to base of cuneus 1.70–1.78 mm (1.68 mm); width across hemelytra 0.74–0.77 mm (0.74 mm). *Head*: Length 0.30–0.32 mm (0.30 mm), width across eyes 0.51–0.53 mm (0.51 mm); interocular width 0.29–0.30 mm (0.27 mm). *Labium*: Length 0.94–1.01 mm (0.90 mm). *Antenna*: Segment I, length (0.37 mm (0.34 mm); II, 1.17–1.20 mm (left 0.91 mm, right 1.06 mm); III, 0.67–0.69 mm (0.64 mm); IV, 0.42–0.43 mm (0.40 mm). *Pronotum*: Length 0.27 mm (0.24 mm), basal width 0.64–0.69 mm (0.67 mm).

*Coloration*: *Head* (Figs 89–91): Semishiny fuscous to black, pale spot on either side of vertex obsolete or indistinct. *Labium*: *Antenna*: Uniformly black, segment I very narrowly pale at apex on some specimens. *Pronotum*: Uniformly pale brownish orange, posterior angles sometimes slightly infuscated; collar pale or whitish; mesoscutum and scutellum uniformly pale brownish orange. *Hemelytron*: Uniformly pale, translucent, brownish orange; membrane translucent smoky brown. *Ostiolar evaporative area* (Fig. 92): Brownish orange to orange. *Ventral surface*: Thoracic segments orange to reddish orange; abdomen in males reddish orange dorsally, paler orange ventrally, with genital capsule becoming fuscous or black; abdomen in females infuscated dorsally, gradually fading to pale orange ventrally. *Legs*: Uniformly pale brownish orange; claw (Fig. 94).

*Structure, texture, and vestiture*: Head: Wider than long, interocular width subequal to length, impunctate, semishiny; set with a few, scattered, erect, nearly bristlelike dark setae. *Labium*: Extending beyond metacoxae to base of abbdomen. *Pronotum*: About 2.5 times as wide at base as long, lateral margins weakly concave, posterior margins moderately flared; set with short, semierect and recumbent setae. *Mesoscutum*: Broadly exposed. *Scutellum*: Slightly wider across base than on sides; set with short, semierect and recumbent setae. *Hemelytron*: Entire in males, cuneus nearly three times as long as wide at base, membrane fully developed, extending well beyond apex of abdomen; set with short, semierect and recumbent setae.

**Figures 179–186.**
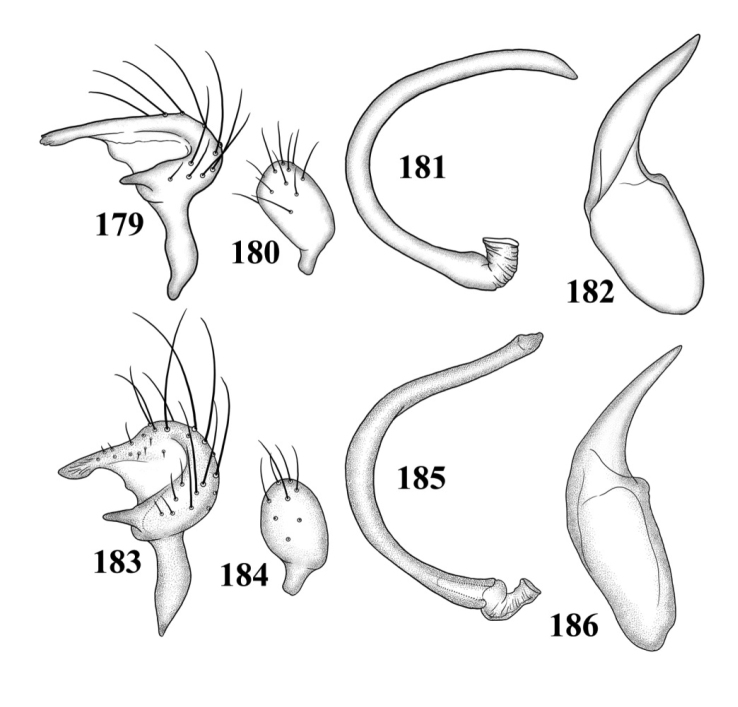
Male genitalia **179–182** *Tytthus uniformis* **179** left paramere **180** right paramere **181** endosoma **182** phallotheca **183–186** *Tytthus vagus* **183** left paramere **184** right paramere **185** endosoma **186**  phallotheca.

*Male genitalia* (Fig. 93): *Left paramere* (Fig. 179): Mitt-shaped; right arm long, slender, apically crenulate; left arm short, apically acute. *Right paramere* (Fig. 180): Oval. *Endosoma* (Fig. 181): Strongly C-shaped, apex bluntly pointed. *Phallotheca* (Fig. 182) slender, apically pointed.

*Macropterous female* (n= 2) (Fig. 46): Length to apex of hemelytron 2.65–2.75 mm, length to base of cuneus 1.88–2.00 mm, width across hemelytra 0.91–1.08 mm. *Head*: Length 0.32–0.34 mm, width across eyes 0.54 mm; interocular width 0.32 mm. *Labium*: Length 0.96–0.98 mm, extending to bases of hind coxae. *Antenna*: Segment I, length 0.34–0.35 mm, II 0.99–1.07 mm; III 0.74–0.77 mm; IV 0.50–0.51 mm. *Pronotum*: Length 0.30 mm, basal width 0.75 mm.

*Brachypterous female* (n= 8) (Figs 47, 95–100): Length to apex of abdomen 1.90–2.23, length to base of cuneus 1.63–1.75 mm, width across hemelytra 0.90–0.93 mm. *Head*: Length 0.34–0.35 mm, width across eyes 0.54–0.56 mm; interocular width 0.34 mm. *Labium*: Length 0.96–0.98 mm, extending to second or third visible abdominal segment. *Antenna*: Segment I, length 0.30–0.32 mm; II, 0.94–1.04 mm; III, 0.40–0.42 mm; IV, 0.40–0.45 mm. *Pronotum*: Length 0.26–0.27 mm, basal width 0.62–0.67 mm.

The hemelytra are abbreviated in nearly all females examined. The cuneal fracture is visible but the cuneus is greatly shortened, with only the narrow basal area of membrane along abbreviated cuneus present.

#### Etymology.

Named for the uniformly pale brownish-orange pronotum, hemelytra, and legs.

**Host.** Big sacaton, *Sporobolus wrightii* Munro ex Scribn. [Poaceae].

#### Distribution.

United States: Arizona and New Mexico.

#### Type material.

**Holotype** ♂ (00162002) (USNM): **UNITED STATES:** **New Mexico:** ***Hidalgo Co*.:** Rt 338, 26.3 km S of Animas, 31.71636°N, 108.82239°W, 1457 m, 15 May 2004, A. G. Wheeler, Jr., *Sporobolus wrightii* (Poaceae). **Paratypes:** **UNITED STATES:** **Arizona:** ***Cochise Co*.:** Huachuca Mountains, 5354 Ash Canyon Road, 0.5 mi W of Hwy 92, 31.38194°N, 110.22444°W, 1554 m, 17 Aug 1992, N. McFarland, 1 ♀ (00162047) (USNM). ***Santa Cruz Co*.:** Audubon Research Ranch, SE of Elgin, 12 - 14 May 2004, A. G. Wheeler, Jr., *Sporobolus wrightii* (Poaceae), 9 ♂♂ (00162021, 00162033 - 00162039, 00162046), 17 ♀♀ (00162022 - 00162032, 00162040 - 00162045) (USNM). **New Mexico:** ***Hidalgo Co*.:** Rt 338, 26.3 km S of Animas, 31.71636°N, 108.82239°W, 1457 m, 15 May 2004, A. G. Wheeler, Jr., *Sporobolus wrightii* (Poaceae), 4 ♂♂ (00162033 - 00162036) (USNM).

### Tytthus vagus

(Knight)

http://species-id.net/wiki/Tytthus_vagus

Figs 48, 49 101–108 183–186

Cyrtorhinus caricis var. *vagus*Knight 1923: 511 (orig. descrip.).
Cyrtorhinus caricis vagus : Blatchley 1926: 853 (descrip., key).
Cyrtorhinus vagus : Knight 1927a: 40 (list).
Tytthus vagus : Carvalho and Southwood 1955 (descrip., n. comb.); Carvalho 1958: 159 (cat.); Henry and Wheeler 1988: 458 (cat.); Schuh 1995: 250 (cat.); Maw et al. 2000: 121 (list).

#### Diagnosis.

This species is distinguished by the black head, pronotum, and scutellum; black antennae; dark translucent smoky brown hemelytra; and pale yellowish brown legs, with the hind femora infuscated on the distal third to one half. All known specimens are macropterous.

*Tytthus vagus* is similar to *Tytthus femoralis* in having dark antennae, pale infuscated hemelytra, and apically infuscated hind femora. It is readily distinguished from *Tytthus femoralis* in lacking distinct fuscous knee spots on the tibiae. It keys out with *Tytthus panamensis* and *Tytthus juturnaiba*, but is separated by the combination of dark antennae and infuscated hind femora.

#### Description.

*Macropterous male* (n = 10, plus holotype in parentheses) (Figs 48, 101, 102): Length to apex of hemelytron 2.56–2.92 mm (2.75 mm), length to base of cuneus 1.18–2.03 mm (2.00 mm), width across hemelytra 0.85–0.96 mm (0.90 mm). *Head*: Length 0.27–0.35 mm (0.30 mm), width across eyes 0.61–0.66 mm (0.59 mm), interocular width 0.30–0.34 mm (0.30 mm). *Labium*: Length 0.88–0.90 mm (0.75 mm). *Antenna*: Segment I length 0.30–0.34 mm (0.34 mm), II 0.83–0.90 mm (0.85 mm), III 0.56–0l.61 mm (missing), IV 0.42–0.50 mm (missing). *Pronotum*: Length 0.30–0.32 mm (0.32 mm), basal width 0.75–0.80 mm (0.80 mm).

*Coloration*: *Head* (Figs 103–105): Uniformly, shiny black, with a distinct, yellow, interocular spot near inner margin of each eye; eyes dark reddish brown; scattered with relatively long, semierect and erect setae. *Labium*: Pale yellow, with apical half of segment IV dark brown. *Antenna*: Segment I fuscous or black, with only apex narrowly pale or yellowish; segments II fuscous to black, with a very narrow pale ring at base; segment III and IV uniformly fuscous. *Pronotum*: Uniformly shiny black. *Mesoscutum*: Fuscous to black. *Scutellum*: Fuscous to black. *Hemelytron*: Dark translucent, smoky brown, becoming darker on clavus. *Ostiolar evaporative area* (Fig. 107): Uniformly fuscous. *Ventral surface*: Thorax and abdomen uniformly fuscous to black. *Legs*: Coxae brown, with only the apices pale; fore and middle coxae uniformly pale yellow, hind coxa yellow with a broad dark brown to fuscous, subapical band; tibiae, tarsi, and claws (Fig. 108) uniformly pale yellow.

*Structure, texture, and vestiture*: *Head*: Uniformly shiny, impunctate, broader than long; buccula relatively narrow, extending posteriorly to near hind margin of eye; set with long, erect and semierect, brown setae. *Labium*: Extending to middle coxae; segment I extending to anterior edge of prosternum. *Antenna*: Segment I with numerous, short, recumbent, brown setae and two, long, erect, black, bristlelike setae subapically; segment II thickly set with short, semierect, brown setae. *Pronotum*: Shiny, impunctate, calli weakly swollen; anterior angles rounded, lateral margins weakly concave, weakly flaring at posterior angles; posterior margin weakly sinuate, almost straight; thickly set with short, semierect, brown setae. *Mesoscutum*: Shiny, impunctate, broadly exposed; with a few scattered, semierect, brown setae. *Scutellum*: Shiny, impunctate, with scattered, semierect, brown setae. *Hemelytron*: Macropterous, subparallel when paired, with fully developed cuneus and membrane, extending well beyond apex of abdomen; evenly clothed with semierect and recumbent, pale brown setae..

*Male genitalia* (Fig. 107): *Left paramere* (Fig. 183): Mitt-shaped, right arm long, broad, and apically rounded; left arm short, slender, and apically acute. *Right paramere* (Fig. 184): Round to weakly oval. *Endosoma* (Fig. 185): C-shaped, bluntly rounded apically. *Phallotheca* (Fig. 186): Slender, apically acute.

*Macropterous female* (n = 10) (Fig. 49): Length to apex of hemelytron 2.80–3.12 mm, length to base of cuneus 2.00–2.28 mm, width across hemelytra 1.07–1.15 mm. *Head*: Length 0.29–0.32 mm, width across eyes 0.59–0.67 mm, interocular width 0.32–0.37 mm. *Labium*: Length 0.93–0.96 mm, extending to bases of middle coxae. *Antenna*: Segment I length 0.27–0.32 mm, II 0.72–0.77 mm, III 0.51–0.56 mm, IV 0.45–0.48 mm. *Pronotum*: Length 0.32–0.35 mm, basal width 0.82–0.90 mm.

#### Hosts.

Taken by A. G. Wheeler on *Spartina alterniflora* and *Spartina bakeri* [Poaceae]. One specimen, collected on trumpet creeper, *Campsis radicans* (L.) Seem ex Bureau [Bignoniaceae], undoubtedly, represents an accidental or sitting record. Denno (in litt, 2005) informed me that *Tytthus vagus* was abundant only on *Spartina alterniflora* in Tuckerton, New Jersey, whereas the sympatric *Tytthus alboornatus* was found only on *Spartina patens*. See Döbel and Denno (1994), Finke and Denno (2003, 2005), Denno et al. (2006) and papers cited therein for in-depth studies on of *Tytthus vagus* and its impact as a predator on delphacid populations on *Spartina alterniflora*.

#### Distribution.

This coastal species is known in Canada from New Brunswick, Newfoundland, and Nova Scotia, and in the United States from Louisiana, Maryland, North Carolina, New Jersey, New York, and Virginia (Knight 1923; Lindberg 1958, Henry and Wheeler 1988, Maw et al. 2000). Polhemus (1994) reported *Tytthus vagus* from Larimer County, Colorado, but this record certainly is an error for another species, probably the Holarctic *Tytthus pygmaeus*, a similar-appearing species with dark antennae.

New state records reported herein are Connecticut, Delaware, Florida, Massachusetts, Mississippi, and South Carolina.

#### Type material examined.

**Holotype**: ♂ (00162339) (USNM): **UNITED STATES:** **New York:** ***Queens Co*.:** Rockaway Beach, Long Island, 40.57138°N, 73.85138°W, 10 Sep 1917, W. A. Hoffman. **Paratypes**: **UNITED STATES: Florida: *Putnam Co*.:** University of Florida Conservation Research Station, 11 May 1946, R. E. Bellamy, 1 ♀ (00166925) (CNC). ***Volusia Co*.:** New Smyrna, Jun 1926, E. D. Ball, 1 ♂ (00162054) (USNM). **New York:** ***Nassau Co*.:** Sea Cliff, Long Island, 40.84889°N, 73.64528°W, Aug 1900, N. Banks, 2 ♀♀ (00162060, 00162061) (USNM); May 1910, collector unknown, 1 ♂ (00162058) (USNM). ***Norfolk Co*.:** Oceanview, 13 Aug 1915, V.A. Roberts, 1 ♂ (00162053) (USNM). ***Queens Co*.:** Rockaway Beach, Long Island, 40.57138°N, 73.85138°W, 10 Sep 1917, W. A. Hoffman, 2 ♂♂ (00167136, 00167137) (CNC), 2 ♂♂ (00162049, 00162050), 2 ♀♀ (00162051, 00162052) (USNM). **New Jersey:** ***Ocean Co*.:** Lakehurst, 40.01444°N, 74.31167°W, Sep 1930, collector unknown, 1 ♀ (00162059) (USNM). **Virginia:** ***Norfolk City*:** Oceanview,12 Aug. 1915, V. A. Roberts, at light (USNM).

#### Other specimens examined.

**CANADA:** **New Brunswick:** Moncton, 46.08333°N, 64.76666°W, 02 Aug 1966, L. A. Kelton, 7 ♂♂ (00167120, 00167129 - 00167134), 3 ♀♀ (00167086 - 00167088) (CNC); 13 Aug 1966, L. A. Kelton, 20 ♂♂ (00167108 - 00167119, 00167121 - 00167128), 24 ♀♀ (00167081 - 00167085, 00167089 - 00167107) (CNC). **Nova Scotia:** Lockeport, 43.6981°N, 65.123°W, 5 m, 21 Jul 1958, J. R. Vockeroth, 6 ♀♀ (00167074 - 00167079) (CNC); 01 Aug 1958, J. R. Vockeroth, 1 ♀ (00167080), 1 ♂ (00167135) (CNC). **UNITED STATES:** **Connecicut: *New Haven Co*.:** Sachem Head, nr. Guilford, 16 Sept. 1966, C. W. O’Brien, 1 ♂, 3 ♀♀ (UCB). **Delaware:** ***Sussex Co*.:** Lewes, 04 Jul 1985, T. J. Henry, *Campsis radicans* (Bignoniaceae), 1 ♂ (00162081) (USNM). **Florida:** ***Duval Co*.:** CR-105, 1 km W. of Dunn Creek, S. of Eastport, 03 Apr 2004, A. G. Wheeler, Jr., *Spartina alterniflora* (Poaceae), 5 ♂♂ (00162063 - 00162067), 8 ♂ (00162087 - 00162093, 00162107) (USNM). Rd. 105, E of Dunn Creek, 30.41663°N, 81.54016°W, 02 Jan 2009, A. G. Wheeler, Jr., *Spartina patens* (Poaceae), 3 ♀♀ (00162094 - 00162096) (USNM). Rd. 105, E of Dunn Creek, 30.41663°N, 81.54016°W, 02 Jan 2009, A. G. Wheeler, Jr., 1 ♂ (00162084) (USNM). ***Levy Co*.:** Cedar Keys, 29.13833°N, 83.03528°W, 03 Aug 1947, R. H. Beamer, 1 ♂ (00162070) (USNM). ***Monroe Co*.:** Everglades National Park, Flamingo Prairie, 08 Apr 1972, R. M. Baranowski, 1 ♀ (00162114) (USNM). ***Osceola Co*.:** CR 532, 1 km E. of CR 545, 3.5 km NNW of Loughman, 05 Apr 2003, A. G. Wheeler, Jr., 2 ♀♀ (00162068, 00162069), 1 ♀ (00162097) (USNM). **Louisiana: *Calcasieu Par*.:** Sulphur, 22 June 1948, H. W. Crowder (KU). ***Orleans Par*.:** New Orleans, 29.9546°N, 90.0751°W, 22 May 2003, APHIS port inspector, 1 ♂ (00162086) (USNM). ***Vermilion Co*.:** Rainey Refuge, 22 Jul 1925, C. C. Sperry, 1 ♀ (00162108), 1 ♂ (00162083) (USNM). **Maryland:** ***St. Marys Co*.:** Piney Point, 38.13535°N, 76.52927°W, 26 Aug 1946, R. I. Sailer, 1 ♂ (00162076), 3 ♀♀ (00162099 - 00162101) (USNM). ***Talbot Co*.:** Wittman, 38.8°N, 76.28333°W, 28 May 2006 - 29 May 2006, W.E. Steiner and J.M. Swearingen, 1 ♂ (00162072) (USNM). **Massachusetts:** ***Essex Co*.:** Gloucester, on beach, 42.62116°N, 70.63135°W, 1 m, 29 Aug 1994, M. D. Schwartz, 2 ♂♂ (00167138, 00167139) (CNC). ***Nantucket Co*.:** Nantucket, 41.28333°N, 70.09944°W, 13 m, 06 Aug 1913, Cushman, 1 ♀ (00162110) (USNM). ***Norfolk Co*.:** Cohasset, 42.24167°N, 70.80417°W, Sep 1908, C. W. Johnson, 1 ♂ (00162082) (USNM). **Mississippi: *Hancock Co*.:** Pearlington, 25 June 1948, H. W. Crowder & R. H. Beamer, 1 ♂, 3 ♀♀ (KU). ***Jackson Co*.:** Ocean Springs, Gulf Coast Research Lab, 05 Jun 1962, D.L. Deonier, 1 ♂ (00162057) (USNM). **New Jersey:** ***Camden Co*.:** Morgan, Jun 1919, Weiss and West, 1 ♂ (00166924) (CNC). ***Cape May Co*.:** Rio Grande, 39.01444°N, 74.88166°W, 6 m, 05 Jul 1985, T. J. Henry, 1 ♀ (00162115) (USNM). **New York:** ***Chenango Co*.:** Earlville, Murphy Farm, Sep 1997, R.A. Byers, 1 ♀ (00162071), 1 ♂ (00162113) (USNM). ***Suffolk Co*.:** Babylon, Long Island, 40.694°N, 73.329°W, Aug 1903, collector unknown, 1 ♀ (00162109) (USNM). Orient, Long Island, 41.13899°N, 72.30342°W, 3 m, 18 Sep 1923, F. M. Schott, 1 ♀ (00162062) (USNM). Locality unknown, 1900, collector unknown, 2 ♂♂ (00162055, 00162056) (USNM). **North Carolina:** ***Camden Co*.:** North River, 17 Oct 1959, L. Davis, 1 ♂ (00162085), 2 ♀♀ (00162111, 00162112) (USNM). ***Carteret Co*.:** Bogue Island, 17 Oct 1974, G. C. Steyskal, 2 ♂♂ (00162074, 00162075) (USNM). **South Carolina:** ***Charleston Co*.:** Charleston, 32.77639°N, 79.93111°W, 10 Jul 1958, D. A. Young, 1 ♂ (00162073) (USNM). ***Colleton Co*.:** CR-26, 1.1 km W. of Bennetts Point, 04 May 2003, A. G. Wheeler, Jr., *Spartina bakeri* (Poaceae), 1 ♀ (00162098) (USNM). **Virginia:** ***Accomack Co*.:** Assateague Island, near Chincoteague, 12 May 1990 - 14 May 1990, W.E. Steiner, J.M. Hill & J.J. Marshall, 3 ♂♂ (00162077 - 00162078, 00162080), 5 ♀♀ (00162102 - 00162106) (USNM). Chincoteague NWR., Assateague Id., Wash Flats, 10 October 1998, SMR, Malaise trap, 1 ♂ (VMNH). ***Norfolk Co*.:** Oceanview, 13 Aug 1915, V.A. Roberts, Paratype, 1 ♂ (00162053) (USNM). **Northampton Co.**: Savage Neck NAP, Custis Pond, 9 July 2004, ACC, UV, 1 ♂, 2 ♀♀ (VMNH).

### Tytthus wheeleri

Henry sp. n.

urn:lsid:zoobank.org:act:1F707E19-3E1C-4A47-BD13-E3027DB5756E

http://species-id.net/wiki/Tytthus_wheeleri

Figs 1 50–52 187–190

#### Diagnosis.

This species, known from brachypterous males and females (Figs 50, 52) and only one macropterous female (Fig. 51), is distinguished by the small size, overall dark brown coloration with only the basal third of the corium and clavus pale or white, the dark brown antennal segment I, and pale yellowish-brown tibiae.

It is similar to *Tytthus montanus* in overall brown coloration with the basal area of the corium and clavus pale, but is distinguished from that species by the much smaller size, dark antennal segment I, and pale tibiae. It is also similar to *Tytthus alboornatus* in overall coloration, but differs in lacking a narrow pale area across the apex of the corium and the dark antennal segment I and pale segment II. All specimens of *Tytthus wheeleri* at hand, except one macropterous female, are strongly brachypterous and lack a cuneus and membrane on each hemelytron.

#### Description.

*Brachypterous male* (n= 10; holotype in parentheses) (Fig. 50): Length to apex of abdomen 1.08–1.28 mm (1.30 mm); length to base of cuneus 0.95–1.05 mm (1.05 mm); width across hemelytra 0.51–0.61 mm (0.56 mm). *Head*: Length 0.24–0.29 mm (0.26 mm), width across eyes 0.43–0.45 mm (0.43 mm); interocular width 0.26–0.29 mm (0.27 mm). *Labium*: Length 0.75–0.77 mm (0.80 mm). *Antenna*: Segment I, length 0.22–0.27 mm (0.26 mm); II, 0.75–0.82 mm (0.83 mm); III, 0.45–0.48 mm (0.51 mm); IV, 0.34–0.40 mm (0.32 mm). *Pronotum*: Length 0.19–0.21 mm (0.21 mm), basal width 0.40–0.48 (0.43 mm).

*Macropterous male*: Unknown.

*Coloration*: Overall coloration dark brown. *Head*: Dark shiny brown, becoming paler yellowish brown ventrally, yellow spot on inner margin of each eye absent; eyes fuscous, often with a reddish tinge. *Antenna*: Segment I dark brown; II, pale yellowish brown, sometimes weakly red tinged at apex; III and IV pale yellowish brown. *Pronotum*: Dark shiny brown; scutellum dark shiny brown with apical half to one third pale yellowish brown. *Hemelytron*: Dark brown pale translucent yellowish brown to whitish on basal one third to half of corium. *Ventral surface*: Thorax dark brown, usually red tinged; ostiolar auricle brown, often red tinged; abdomen dark brown laterally, becoming paler ventrally, often tinged with red at base. *Legs*: Uniformly pale yellowish brown, except for dark brown hind femora with basal third and narrow apex pale yellowish brown.

*Structure, texture, and vestiture*: *Head*: Wider than long, impunctate, eyes prominent, frons swollen, interocular width much greater than combined dorsal diameter of eyes. *Labium*: Extending just past metacoxae to base of abdomen. *Pronotum*: Impunctate, at most with only a few fine punctures across middle; rectangular, wider than long, lateral margins nearly straight, posterior margin weakly sinuate in brachypterous forms; lateral margins concave, posterior angles widely flared in macropterous form; calli weakly developed; scutellum impunctate, equilateral. *Hemelytron*: Strongly brachypterous in all but one specimen; corium and clavus fused (claval suture absent), membrane and cuneus absent, remaining corium weakly rounded laterally, truncate posteriorly. Hemelytron of only known macropter with fully developed clavus, cuneus, and membrane having two closed cells. Pubescence short, sparse, with several erect, bristlelike setae on head along basal margin, inner margin of eyes, and on frons.

**Figures 187–193.**
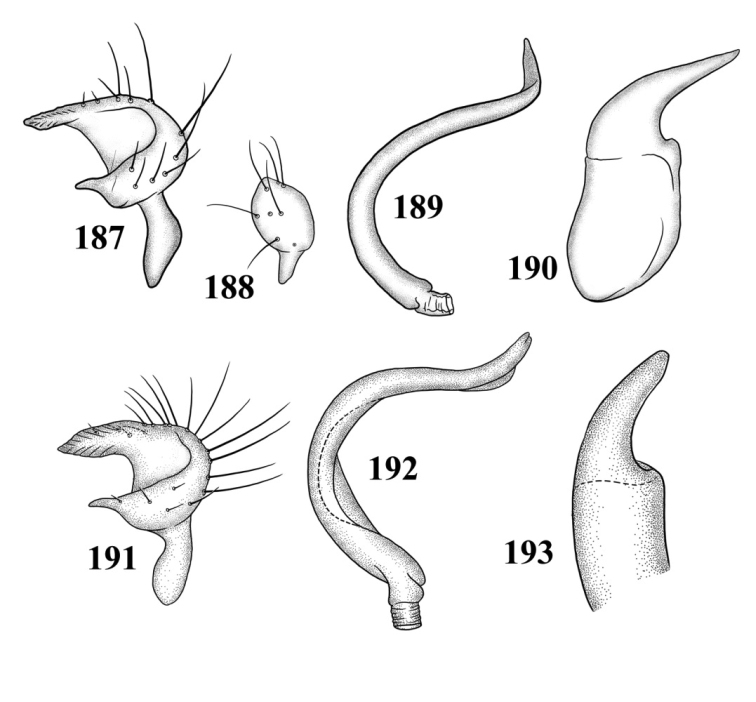
Male genitalia **187–190** *Tytthus wheeleri* **187** left paramere **188** right paramere **189** endosoma **190** phallotheca **191–193** *Tytthus zwaluwenburgi* **191** left paramere **192** endosoma **193** phallotheca.

*Male genitalia*: *Left paramere* (Fig. 187): Mitt-shaped; right arm long, stout, apically bluntly rounded; left arm short, apically acute. *Right paramere* (Fig. 188): Rounded. *Endosoma* (Fig. 189): S-shaped, apically acute. *Phallotheca* (Fig. 190): Slender, apically acute.

*Macropterous female* (n= 1) (Fig. 51): Length to apex of hemelytron 1.80 mm, length to base of cuneus 1.27 mm, width across hemelytra 0.62 mm. *Head*: Length 0.26 mm, width across eyes 0.42 mm; interocular width 0.27 mm. *Labium*: Length 0.83. *Antenna*: Segment I, length 0.22 mm; II, 0.62 mm; III, 0.43 mm; IV, 0.27 mm. *Pronotum*: Length 0.24 mm, basal width 0.59 mm.

*Brachypterous female* (n= 10) (Fig. 52): Length to apex of abdomen 1.48–1.68 mm, length to apex of hemelytron 1.23–1.30 mm, width across hemelytra 0.70–0.74 mm. *Head*: Length 0.26–0.34 mm, width across eyes 0.46 mm; interocular width 0.29–0.30 mm. *Labium*: Length 0.93–0.96 mm. *Antenna*: Segment I, length 0.22–0.26 mm; II, 0.72–0.77 mm; III, 0.48–0.54 mm; IV, 0.37–0.40 mm. *Pronotum*: Length 0.21–0.22 mm, basal width 0.48–0.51 mm.

#### Etymology.

This species is named after my good friend and colleague, Dr. Alfred G. Wheeler, Jr. (Department of Entomology, Clemson University, Clemson, South Carolina), who discovered and collected all but a few specimens of this very tiny, attractive new species. Dr. Wheeler’s collections from grasses having well-developed crowns have yielded numerous new and interesting Heteroptera.

#### Hosts.

Collected by A. G. Wheeler on the following hosts: big bluestem, *Andropogon gerardii* Vitman [Poaceae]; bushy bluestem, *Andropogon glomeratus* (Walt.) B. S. P.; bushy bluestem, *Andropogon tenuispatheus* Nash [sometimes considered a synonym of *Andropogon glomeratus* (Walt.) B. S. P. var. *pumilus* Vasey ex. L. H. Dewey]; Virginia beard grass, *Andropogon viriginicus* L.; weeping love grass, *Eragrostis curvula* (Schrad.) Nees; gulf cordgrass, *Spartina spartinae* (Trin. Merr. ex Hitchc.; and paragrass, *Urochloa mutica* (Forssk.) T. Q. Nguyen [Poaceae].

#### Distribution.

United States:Arkansas, Florida, Georgia, Kansas, Nebraska, South Carolina, Texas, and Virginia.

#### Discussion.

All specimens of this new species are brachypterous, except for one macropterous female collected in Highlands Co., Florida (March). That a macropterous female was discovered indicates that macropterous males eventually should be found. Given the small size and considering how difficult it is to collect this species from the crowns of certain bunch grasses, it is not surprising that few undetermined specimens of this species were found in collections.

The tiny brachypterous males (length 1.08–1.30 mm) rank *Tytthus wheeleri* as possibly the smallest known species of Miridae.

#### Type material.

**Holotype**: ♂ (00162128)(USNM): **UNITED STATES: South Carolina:** ***Richland Co*.:** Spears Creek Church Road, 3.8 km S. of Pontiac, 06 May 2000, A. G. Wheeler, Jr., *Eragrostis curvula* (Poaceae). **Paratypes**: **UNITED STATES:** **Arkansas:** ***Ashley Co*.:** Rt. 82, 13.5 km W. of Crossett, 19 May 2000, A. G. Wheeler, Jr., *Eragrostis curvula* (Poaceae), 1 ♀ (00162145) (USNM). **Florida: *Miami-Dade Co*.**: Homestead, 14 Mar 1947, R. H. Beamer (UK). Brevard Co., CR-520, 0.5 km E of Orange Co., nr St. Johns River, W of Cocoa, 11 April 2004, A. G. Wheeler, Jr., ex *Urochloa mutica*, 1 ♂ (macropterous) (USNM). ***Highlands Co*.:** Archbold Biological Station, Hufty Tract, 17 Apr 1998, T. J. Henry, A. G. Wheeler, and T. & M. Yasunaga, *Andropogon glomerata* (Poaceae), 3 ♀♀ (00162151 - 00162153) (USNM). Rt. 70 at Lake Annie, SW of Lake Placid, 17 Mar 1999, A. G. Wheeler, Jr., *Andropogon glomerata* (Poaceae), 2 ♂♂ (00162116, 00162117), 3 ♀♀ (00162118 - 00162120) (USNM). ***Levy Co*.:** Manatee Springs State Park, 6 mi. W. of Chiefland, 16 Mar 1999, A. G. Wheeler, Jr., *Eragrostis virginicus* (Poaceae), 2 ♂♂ (00162134, 00162135) *Andropogon virginicus* (Poaceae), 1 ♂ (00162127) (USNM). ***Polk Co*.:** Rt. 27, 2.4 km N. of jct. CR-54, NW of Loughman, 26 Feb 2002, A. G. Wheeler, Jr., *Eragrostis curvula* (Poaceae), 4 ♂♂ (and 1 nymph) (00162121 - 00162123, 00162125), 1 ♀ (00162126) (USNM); 13 Apr 2002, A. G. Wheeler, Jr., *Eragrostis curvula* (Poaceae), 1 ♀ (00162133) (USNM). **Georgia:** ***Columbia Co*.:** Dixie Mountain, Serpentine Barren, near Pollards Corner, 06 May 1996, A. G. Wheeler, Jr., *Andropogon gerardii* (Poaceae), 1 ♂ (00162137) (USNM). ***Peach Co*.:** Rt. 49, SW of Byron, 25 Sep 1996, A. G. Wheeler, Jr., *Andropogon virginicus* (Poaceae), 1 ♂ (and 1 nymph), (00162139) *Andropogon* spp. (Poaceae), 1 ♂ (00162138) (USNM). **Nebraska:** ***Keith Co*.:** Rt. 61 at Kingsley Drive, 8 mi. N. of Ogallala, 15 Jun 1998, A. G. Wheeler, Jr., 2 ♂♂ (and 2 nymphs) (00162141, 00162144) (USNM). **Kansas: *Jefferson Co*.:** Rockefeller Tract, ca 5 mi N of Laurence, 9-15 June 1976, A. Slater, 1 ♂, 3 ♀♀ (AMNH). **South Carolina:** ***Aiken Co*.:** Rt. 19, 3 mi. N. of Aiken, 18 Aug 1996, A. G. Wheeler, Jr., *Eragrostis curvula* (Poaceae), 1 ♂ (00162131) (USNM). ***Lancaster Co*.:** Forty Acre Rock, 04 May 1996, A. G. Wheeler, Jr., *Andropogon gerardii* (Poaceae), 1 ♂ (00162150) (USNM). ***Laurens Co*.:** Rt. 25, 3.3 km N. of Saluda River, NNE of Ware Shoals, 21 Oct 2001, A. G. Wheeler, Jr., *Eragrostis curvula* (Poaceae), 1 ♂ (00162129) (USNM). ***Pickens Co*.:** Glassy Mountain East of Pickens, 10 May 1997, A. G. Wheeler, Jr., *Eragrostis curvula* (Poaceae), 1 ♀ (00162136) (USNM). Glassy Mountain Heritage Preserve, E. of Pickens, 24 May 2003, A. G. Wheeler, Jr., *Eragrostis curvula* (Poaceae), 1 ♂, 1 ♀ (USNM). S-39-143, 2 km W of Eastatoe Creek, 34.96833°N, 82.865°W, 05 Oct 2001, A. G. Wheeler, Jr., *Eragrostis curvula* (Poaceae), 1 ♀ (00162130) (USNM), 1 ♀ (00162148) (USNM). **Texas:** ***Burnet Co*.:** Rt. 71, 2 km SW of Spicewood, 07 Jun 2003, A. G. Wheeler, Jr., *Muhlenbergia lindheimeri* (Poaceae), 1 ♂ (00162149), plus 1 ♀ (USNM). ***Gillespie Co*.:** Rt. 16, 15 km NE of Fredericksburg, 30.36°N, 98.75°W, 27 May 2001, A. G. Wheeler, Jr., *Muhlenbergia lindheimeri* (Poaceae), 1 ♂ (00162157) (USNM). Farm Rd 1888, 2.4 km NW of Kendall Co. line, SE of Luckenbach, 28 May 2001, A. G. Wheeler, Jr., ex *Muhlenbergia lindheimeri* (Poaceae), 1 ♀. ***Gonzales Co*.**: Palmetto St. Park, Ottine, 29E35.63'N, 97E35.12'W, 15 May 2006, A. G. Wheeler, Jr., ex *Spartina spartinae*, 3 ♂♂, 1 ♀ (USNM). **Virginia:** ***Mecklenburg Co*.:** Elm Hill SGM at Clyde’s Pond, 11 May 1995 - 29 May 1995, VMNH Survey, 2 ♀♀ (00162155, 00162156) (USNM). ***Sussex Co*.:** SW of Littleton, chub sandhill, 01 Jun 1996, A. G. Wheeler, Jr., *Andropogon* sp. (Poaceae), 1 ♂ (00162132) (USNM).

### Tytthus zwaluwenburgi

Usinger

http://species-id.net/wiki/Tytthus_zwaluwenburgi

Figs 53, 54 191–193

Cyrtorhinus zwaluwenburgi Usinger 1944: 148 (orig. descrip.), 1951: 5 (key).
Tytthus zwaluwenburgi : Carvalho and Southwood 1955: 19 (descrip.); Carvalho 1958: 159 (cat.); Schuh 1984: 192 (diag., host), 1995: 250 (cat.).

#### Diagnosis.

This species is readily distinguished by the uniformly yellow to testaceous coloration, except for the dark brown eyes and a vague brown area at the middle of the head.

#### Description.

*Male* (n = 1 paratype) (Fig. 53): Length to apex of hemelytron 2.30 mm, length to base of cuneus 1.66 mm, width across hemelytra 0.77 mm. *Head*: Length 0.29 mm, width across eyes 0.56 mm, interocular width 0.30 mm. *Labium*: Length 0.74 mm. *Antenna* (missing; after Schuh 1984): Segment I length (not given), II 0.61 mm, III (not given), IV (not given). *Pronotum*: Length 0.27 mm, basal width 0.75 mm.

*Coloration*: Uniformly yellow to testaceous, except for the dark brown eyes and a vague brown area on the middle of the head.

*Structure, texture, and vestiture* (after Usinger 1944): Head half again as broad as long, 11.5; 8, smooth, shining, and strongly convex above. Eyes slightly less than half as wide as interocular space, 2.75: 6. First antennal segment shorter than interocular space, 5: 6, second segment three times as long as first, third and fourth segments broken off. Rostrum nearly reaching apices of middle coxae. Pronotum somewhat duller than head, clothed with short, sparse, decumbent hairs; broader across humeri than width of head, 15: 11.5, and less than half as long as broad, 6.5: 15; front margin shallowly concave, lateral margins feebly sinuate, and hind margin slightly concave. Scutellum longer than pronotum at middle, 7: 6, subdepressed, the disk very sparsely clothed with appressed hairs. Hemelytron simple, distinctly but sparsely clothed with appressed hairs; costal margin slightly, evenly arcuate. Legs slender, clothed with short, inconspicuous, pale hairs. Claws with simple hairlike setae rather than arolia.

*Male genitalia* (after Schuh 1984): *Left paramere* (Fig. 191): Mitt-shaped; right arm stout, broad, apically acute; left arm slender, apically acute. *Right paramere*: Not illustrated. *Endosoma* (Fig. 192): S-shaped. *Phallotheca* (Fig. 193): Slender, bluntly rounded apically.

*Female*: (n = 1) (Fig. 54): Length to apex of hemelytron 2.53 mm, length to base of cuneus 1.80 mm, width across hemelytra 0.96 mm. *Head*: Length 0.29 mm, width across eyes 0.59 mm, interocular width 0.34 mm. *Labium*: Length 0.85 mm. *Antenna*: Segment I length 0.24 mm, II 0.61 mm, III and IV missing. *Pronotum*: Length 0.32 mm, basal width 0.80 mm.

#### Hosts.

Recorded from *Boerhavia* sp. (Nyctaginaceae) by Usinger (1944) and Schuh (1984). Usinger (1944) noted that the various species of the genus prey on delphacid eggs, but since “delphacids have not been reported on Canton Island and since both *Cyrtorhinus* and the cicadellid *Nesaloha cantonis* Oman were collected on *Boerhaavia*, it is possible that this new mirid is a predator on *Nesaloha*.”

#### Distribution.

This species has been reported from Baker Island, Howland Island, and the Phoenix Islands (Canton Island) in the central Pacific (Usinger 1944, Schuh 1984).

#### Discussion.

*Tytthus zwaluwenburgi* was described from only three specimens (holotype, paratype, and one teneral specimen). Schuh (1984) studied the holotype and illustrated the male genitalia.

#### Type material examined.

**Canton Island**: 20 Nov 1940, R. Danner, 1 ♂ paratype (BPBM).

#### Other specimen examined.

**Baker Island**: 18 Apr 1935, E. H. Bryan, Jr., 1 ♀ (BPBM). Phoenix Islands: Canton Island, 16 Mar 1924, E. H. Bryan, Jr., 1 ♀ (BPBM).

## Phylogeny

Menard (2011) and Menard and Schuh (2011) have shown that *Tytthus* does not belong in Leucophoropterini based on both molecular sequence data and morphology and, thus, they transferred *Tytthus* to nominate tribe Phylini where it shows a relationship with several New World genera, including *Criocoris* Fieber, *Semium* Reuter, and *Spanagonicus* Berg. These finding are supported by Kelton’s (1959) work that showed similarities in the male genitalia among these genera. The relationship of *Tytthus* to New World genera, the restriction to the Western Hemisphere of 17 of its 24 species, and the Holarctic distribution of two other species suggest a New World origin. Only the far-eastern *Tytthus chinensis*, the Indo-Pacific *Tytthus mundulus*, and *Tytthus zwaluwenbergi* found on a few central Pacific islands are restricted to the Old World.

The morphology-based phylogeny of *Tytthus* presented here should be considered tentative because of the limited number of informative characters available to infer relationships. Although most species possess distinctions that allow relatively easy separation, characters, such as antennal, hemelytral, and leg coloration, are very homoplastic and did not offer much resolution. The male genitalia are relatively simple structures (e.g., Figs 109–116) and lack an apparent secondary gonopore, which is shared with several outgroup taxa, including species of the genera *Criocoris* Fieber and *Semium* Reuter (Kelton 1955, Menard 2011, Henry personal observ.). As a consequence, the matrix has fewer characters than taxa.

**Figures 194–197.**
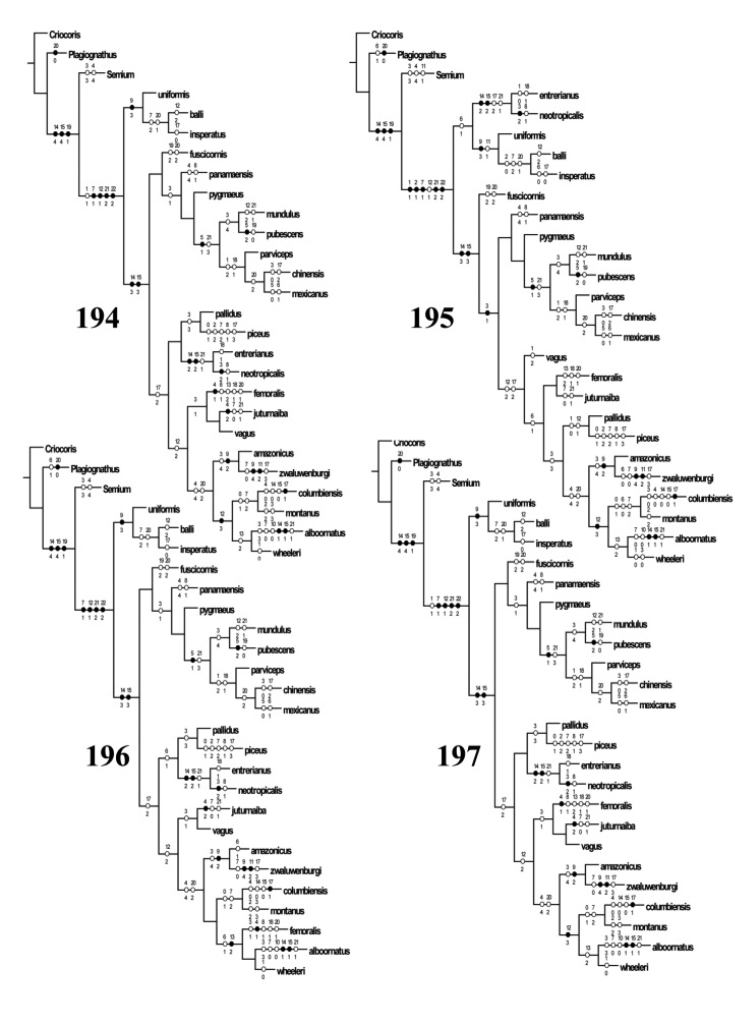
Four resulting cladograms reflecting inferred *Tytthus* phylogeny generated by Winclada, running NONA, based on 27 taxa and 23 characters (Table 1), with character 17 deactivated. Black circles indicate nonhomoplasious characters; white circles indicate homoplasies; character number above branch, character state below.

**Figures 198–200.**
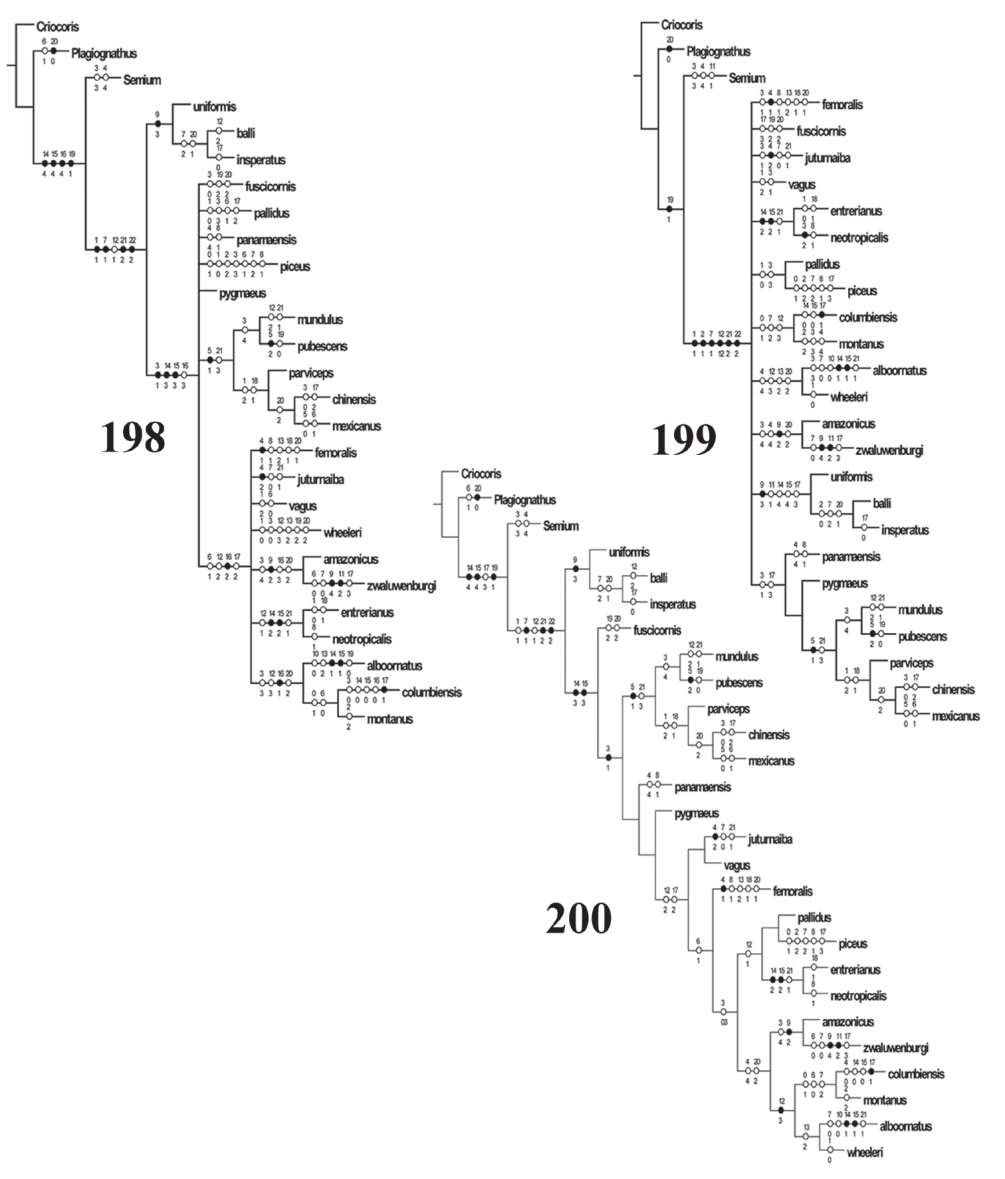
Consensus trees generated by NONA and single cladogram, by TNT, based on 27 taxa and 23 characters (Table 1) **198** strict consensus tree based on nine cladograms generated by Winclada, running NONA, with all characters activated **199** strict consensus tree based on nine cladograms generated by Winclada, running NONA, with character 17 deactivated **200** single cladogram generated by Winclada, running the random addition sequence analysis by TNT, with character 17 deactivated. Black circles indicate nonhomoplasious characters; white circles indicate homoplasies; character number above branch, character state below.

For this analysis, the matrix (Table 1) contained 27 species-group taxa and 23 characters, 17 of which were multistate. Characters (Table 2) were first processed using WinClada (Nixon 1999–2002) to run NONA (Goboloff 1999), using the default settings for the ratchet function. Three taxa (*Tytthus chinensis*, *Tytthus parviceps*, and *Tytthus pygmaeus*) were scored polymorphic for pronotal coloration. Four characters (6, antennal segment II setae; 10, pronotal color; 13, hemelytral color; 19, knee spots) were scored additive because they reflected clear relationships among certain taxa, whereas homologies for all remaining characters were uncertain and, thus, scored as nonadditive. The genera *Criocoris*, *Plagiognathus* Fieber, and *Semium* were used as outgroups. The analysis with all characters activated resulted in nine most parsimonius cladograms, with a length of 117 steps, a ci of 47, and an ri of 60. Generation of a strict consensus (Fig. 198) tree collapsed 10 nodes, reflecting the considerable homoplasy in the data. Manipulation of certain characters with very low consistencies, led to the conclusion that character 17 (hind femur color) was very uniformative, which significantly skewed the results. By deactivating, character 17, only four trees (Figs 194–197) were generated, with a length of 110, a ci of 46, and and ri of 59 The resulting consensus tree (Fig. 199) collapsed 9 nodes and resulted in a topology not too dissimilar to the previous analysis.

**Table 1.** Matrix of 27 taxa and 23 (0-22) characters used for phylogenetic analysis of the genus *Tytthus*. Missing data are indicated by a question mark; polymorphic characters are indicated by a dollar sign.<br/>

**Taxon**	**1111111111222<br/> 01234567890123456789012**
*Criocoris*	00000000000000000000300
*Plagiognathus*	00000010001000000000000
*Semium*	00034000001100444401300
*alboornatus*	01134010000032111200212
*amazonicus*	02144011021020333201222
*balli*	01000012031120444301122
*chinensis*	021001010$1010333221232
*columbiensis*	11100002001030000101??2
*entrerianus*	001000?1001010222211312
*femoralis*	01111011101022332221122
*fuscicornis*	01100001001010333302222
*insperatus*	01000002031110444001122
*juturnaiba*	0?1120?0?010203??20?312
*mexicanus*	02110011001010333311232
*montanus*	11234002001030331201232
*mundulus*	01140101001020333301312
*neotropicalis*	01120011101010222201312
*pallidus*	00130011001010333201?22
*panamaensis*	01114001101010333301??2
*parviceps*	021101010$1010333311332
*piceus*	10230012101010333301322
*pubescens*	01140201001010333300332
*pygmaeus*	011100010$1010333301322
*uniformis*	01100011031110444301322
*vagus*	02110001001020332201322
*wheeleri*	00104011001032332202222
*zwaluwenburgi*	0?14400004122033330122?

**Table 2.** Characters and character states used in analysis of the genus *Tytthus*. The four left columns represent **1** character number **2** number of steps **3** consistency index, and **4** retention index. Character statistics are from one of nine trees (Fig. 194) generated by WinClada to run NONA using the ratchet function.<br/>

0.	2	50	50	Head shape: much broader than long (0); strongly rounded, bulbous (1).
1.	7	28	50	Interocular area adjacent to inner margin of each eye: unmarked (0); with a small yellow spot (1); with a large yellow spot (2).
2.	4	50	60	Clypeus: strongly protruding anteriorly (0); weakly protruding anteriorly, visible dorsally (1); rounded, not visible dorsally (2).
3.	12	33	38	Antennal segment I: uniformly dark brown or fuscous (0); dark brown or fuscous, with apical fourth to one third pale (1); pale at apex and base, with a broad fuscous band through middle (2); pale brownish yellow, with only base narrowly fuscous (3); uniformly pale (4).
4.	6	50	50	Antennal segment II: uniformly dark brown or fuscous (0); pale brownish yellow, with base fuscous (1); pale brownish yellow, with base and apex fuscous (2); uniformly pale brownish yellow (3).
5.	3	50	50	Antennal segment II: without specialized setae (0); males with erect, brushlike setae most prevalent along entire undersurface (1); male and females with long, erect setae as long as diameter of segment (2).
6.	8	12	30	Labium length: Extending to middle coxae or based of hind coxae (0); extending slightly beyond hind coxae on (1).
7.	6	33	55	Pronotal shape of macropters: trapeziform (0); lateral margins weakly concave, posterior angles moderately widened (1); lateral margin strongly concave, posterior angles strongly flared, companulate (2).
8.	4	25	0	Pronotal surface: not modified (0); with a distinct glaucous sheen on anterior lobe (1).
9.	7	57	72	Pronotum: Uniformly dark brown or fuscous (0); dark brown with anterior area around calli yellow (1); pale yellowish brown invaded with darker brown (2); pale orange, sometimes tinged with brown (3) uniformly pale yellow (4).
10.	2	50	0	Hemelytra: weakly to strongly convex laterally (0); straight or subparallel (1).
11.	3	66	66	Scutellum: Uniformly dark brown to fuscous (0); uniformly orange, sometimes with apex pale (1); uniformly pale yellowish green (2).
12.	6	50	80	Hemelytral color: Mostly dark, opaque (0); uniformly translucent white or yellowish to brownish white (1); translucent white, tinged with brown or darker areas on corium and clavus (2); largely dark brown, with base of corium and clavus and, sometimes, apex of corium pale (3).
13.	2	50	50	Hemelytral development in males: Fully macropterous (0); submacropterous, with membrane and cuneus abbreviated (1); staphylinoid or brachypterous, with membrane and cuneus absent, not extending to apex of abdomen (2).
14.	5	80	83	Fore femoral color: Uniformly dark brown or fuscous (0); dark brown with base and apex pale (1); pale (2); largely pale, with apex of infuscated (3); uniformly pale yellowish (4); uniformly pale orange (5).
15.	5	80	83	Middle femoral color: Uniformly dark brown or fuscous (0); dark brown with base and apex pale (1) pale (2); largely pale, with apex infuscated (3); uniformly pale yellowish (4); uniformly pale orange (5).
16.	6	66	80	Hind femoral color: Uniformly dark brown or fuscous (0); dark brown with base and apex pale (1); pale, invaded with infuscous (2); largely pale, with apices of all femora infuscated (3); uniformly pale yellowish (4); uniformly pale orange (5).
17.	7	57	75	Tibial color: Uniformly dark brown (0); uniformly pale yellowish brown (1); uniformly orange, sometimes with a short, narrow red basal stripe (2).
18.	5	40	40	Tibial knees: without fuscous knee spots (0); with fuscous knee spots (1) .
19.	5	40	25	Hind tibial spines: strong, dark (0); slender, reduced, usually pale (1); absent or indistinct, resembling surrounding setae (2).
20.	7	42	55	Endosoma shape: variably complex, not as in state 1, secondary gonopore distinct (0); weakly S-shaped, secondary gonopore absent (1); C-shaped, secondary gonopore absent (2).
21.	6	50	70	Left paramere: left arm slender (0); thickened, apically pointed (1); thickened, apically blunt (2); left arm greatly broadened, swordlike (3)
22.	1	100	100	Habits: species phytophagous, associated with specific host plants (0); species predatory, associated with specific Delphacidae (Auchenorrhyncha) (1)

The genus *Tytthus* is defined by a combination of the yellow spots on interocular area, bell-shaped or campanulate pronotum, simple S- or C-shaped endosoma, and host specificity (eggs of Delphacidae). In all trees (Figs 194–197), three monophyletic species groups were hypothesized: the *balli* group (*Tytthus balli, T. insperatus, and T. uniformis*), the *chinensis* group (*T. pymaeus*, *Tytthus mundulus*, *Tytthus pubescens*, *Tytthus parviceps*, *Tytthus chinensis*, and *Tytthus mexicanus*), and the *alboornatus* group (*alboornatus, columbiensis, montanus, and wheeleri*). The *balli* group was defined by one synapomorphy, the pale orange pronotum (character 9, state 3). These species are also share a broad black head, a strongly campanulate pronotum, and orange or largely orange scutellum, hemelytra, and legs. The *chinensis* group was defined by one synapomorphy, the brushlike setae on antennal segment II (character 5, state 1), and the broad swordlike left arm of the left paramere (character 21, state 3). Within this group, *Tytthus chinensis*, T.* mexicanus*, and *Tytthus parviceps* shared the fuscous knee spots (character 18, state 1), found elsewhere only in *Tytthus entrerianus* and *Tytthus femoralis*. The *alboornatus* group was defined by one synapomorphy, the largely dark brown hemelytra with pale areas at the base and apex of the corium (character 12, state 3). Also, within this group, *Tytthus alboornatus* and *Tytthus wheeleri* possess the most extreme hemelytral brachyptery in the genus; brachyptery is found to a lesser extent in two other members of the group, *Tytthus montanus*, and *Tytthus piceus*, the latter of which falls outside the group. *Tytthus entrerianus* and *Tytthus neotropicalis* always came out as sister species, based on two synapomorphies, the pale fore and middle femora (characters 14, state 2, ♀ character 15, state 2), as did *Tytthus pallidus* and *Tytthus piceus*, based on the color of the hemelytra (character 12, state 1) and the pale antennal segment I (character 3, state 3). *Tytthus zwaluwenburgi* is the most problematic species of the genus. Although it came out as the sister species to *Tytthus amazonicus*, this relationship, supported only by color characters (character 3, state 4 ♀ character 9, state 2), is unlikely to be correct. Its pale coloration and extreme isolation on islands in the central Pacific preclude any meaningful hypothesis about its relationship to other species. The characters for most remaining taxa exhibited considerably more homoplasy and, thus, their hypothesized position among the species was less stable.

The same character matrix (Table 1) as above, with 27 species-group taxa and 23 characters (with the same four characters scored as additive and character 17 deactivated), also was run using TNT (Goloboff et al. 2010). A first pass using the traditional analysis setting resulted in 29 trees with a length of 116, a ci of 46, and an ri of 60. An additional run using the random addition sequence function resulted in only one most parsimonius tree (Fig. 200), with a length of 109, a ci of 45, and an ri of 59. This tree was far more resolved than either of the above strict consensus trees (Fig. 198, 199) generated by Nona and most closely resembled tree number one (Fig. 194) of the four generated by Nona with character 17 deactivated. *Tytthus uniformis*, *Tytthus balli*, and *Tytthus insperatus* were hypothesized as a monophyletic group, as were *Tytthus mundulus*, *Tytthus pubescens*, *Tytthus parviceps*, *Tytthus chinensis*, and *Tytthus mexicanus*. In sequence, *Tytthus panamaensis* was hypothesized as sister to the remainder of the taxa, followed by the holoarctic *Tytthus pygmaeus*. *Tytthus juturnaiba* and *Tytthus vagus* formed a sister pair, as did *Tytthus pallidus* and *Tytthus piceus* and *Tytthus entrerianus* and *Tytthus neotropicalis*. Also, as in all of the previous analyses, *Tytthus columbiensis*, *Tytthus montanus*, *Tytthus alboornatus*, and *Tytthus wheeleri* formed a monophyletic group. The problematic *Tytthus zwaluwenburgi* still grouped with *Tytthus amazonicus*, a doubtful relationship.

As noted in my discussion of *Tytthus chinensis* and *Tytthus parviceps*, molecular data likely will help resolve some of the unclear relationships among the species of this intriguing genus of egg predators. If *Tytthus juturnaiba* proves to be a junior synonym of *Tytthus neotropicalis*, the removal of that species and its conflicting character information should help reduce the level of homoplasy in certain characters and likely will yield a better hypothesis of relationships.

## Supplementary Material

XML Treatment for Tytthus

XML Treatment for Tytthus alboornatus

XML Treatment for Tytthus amazonicus

XML Treatment for Tytthus balli

XML Treatment for Tytthus chinensis

XML Treatment for Tytthus columbiensis

XML Treatment for Tytthus entrerianus

XML Treatment for Tytthus femoralis

XML Treatment for Tytthus fuscicornis

XML Treatment for Tytthus insperatus

XML Treatment for Tytthus juturnaiba

XML Treatment for Tytthus mexicanus

XML Treatment for Tytthus montanus

XML Treatment for Tytthus mundulus

XML Treatment for Tytthus neotropicalis

XML Treatment for Tytthus pallidus

XML Treatment for Tytthus panamensis

XML Treatment for Tytthus parviceps

XML Treatment for Tytthus piceus

XML Treatment for Tytthus pubescens

XML Treatment for Tytthus pygmaeus

XML Treatment for Tytthus uniformis

XML Treatment for Tytthus vagus

XML Treatment for Tytthus wheeleri

XML Treatment for Tytthus zwaluwenburgi
